# Emergent neural dynamics and geometry for generalization in a transitive inference task

**DOI:** 10.1371/journal.pcbi.1011954

**Published:** 2024-04-25

**Authors:** Kenneth Kay, Natalie Biderman, Ramin Khajeh, Manuel Beiran, Christopher J. Cueva, Daphna Shohamy, Greg Jensen, Xue-Xin Wei, Vincent P. Ferrera, LF Abbott

**Affiliations:** 1 Mortimer B. Zuckerman Mind Brain Behavior Institute, Columbia University, New York, New York, United States of America; 2 Center for Theoretical Neuroscience, Columbia University, New York, New York, United States of America; 3 Grossman Center for the Statistics of Mind, Columbia University, New York, New York, United States of America; 4 Department of Psychology, Columbia University, New York, New York, United States of America; 5 Department of Brain and Cognitive Sciences, MIT, Cambridge, Massachusetts, United States of America; 6 The Kavli Institute for Brain Science, Columbia University, New York, New York, United States of America; 7 Department of Neuroscience, Columbia University Medical Center, New York, New York, United States of America; 8 Department of Psychology at Reed College, Portland, Oregon, United States of America; 9 Departments of Neuroscience and Psychology, The University of Texas at Austin, Austin, Texas, United States of America; 10 Department of Psychiatry, Columbia University Medical Center, New York, New York, United States of America; Brown University, UNITED STATES

## Abstract

Relational cognition—the ability to infer relationships that generalize to novel combinations of objects—is fundamental to human and animal intelligence. Despite this importance, it remains unclear how relational cognition is implemented in the brain due in part to a lack of hypotheses and predictions at the levels of collective neural activity and behavior. Here we discovered, analyzed, and experimentally tested neural networks (NNs) that perform transitive inference (TI), a classic relational task (if A > B and B > C, then A > C). We found NNs that (i) generalized perfectly, despite lacking overt transitive structure prior to training, (ii) generalized when the task required working memory (WM), a capacity thought to be essential to inference in the brain, (iii) emergently expressed behaviors long observed in living subjects, in addition to a novel order-dependent behavior, and (iv) expressed different task solutions yielding alternative behavioral and neural predictions. Further, in a large-scale experiment, we found that human subjects performing WM-based TI showed behavior inconsistent with a class of NNs that characteristically expressed an intuitive task solution. These findings provide neural insights into a classical relational ability, with wider implications for how the brain realizes relational cognition.

## Author summary

The ability to infer underlying relationships in the world—or “relational cognition”—is fundamental to both human and animal intelligence. Despite this importance, it remains generally unknown how relational abilities are realized in the brain. Addressing this lack of knowledge requires generating testable hypotheses regarding the activity of neurons when relationships are inferred. In this study, we discovered artificial neural networks that perform transitive inference (TI), a classic relational task that has the form “if A > B and B > C, then A > C.” We found that these neural networks show behavioral patterns that match those seen in humans and animals performing TI, and, further, performed TI in different ways, thereby providing different testable hypotheses regarding neural activity. We also conducted a large-scale behavioral study in humans, which suggested systematic differences in biological accuracy across the neural networks. These findings provide new hypotheses and insights regarding how TI, and relational abilities more generally, can be realized in the brain.

## Introduction

Cognitive faculties such as logical reasoning, mathematics, and language have long been recognized as characteristic of human-level intelligence. Common to these faculties is abstraction: the ability to generalize prior knowledge and experience to novel circumstances. Importantly, abstraction typically entails understanding particular relationships, e.g. “adjacent to”, “same as”, “relevant to”) between items (e.g. stimuli, objects, behaviors, words, variables), which can then be used to infer equivalent relationships between items not previously observed together, i.e. *novel combinations of items*. Such relational inferences—which can also be understood as systematic generalizations to novel compositions of inputs—are the basis of a structured form of knowledge, often termed a “schema,” that is thought to enable humans to generalize in systematic and meaningful ways [1–6], and thus has been posited as essential to advanced cognition.

Intriguingly, landmark work in animals [7–12] indicates that cognition based on relations is more prevalent, and thus possibly more essential, than previously thought. This prevalence is evidenced by the observation that cognitive abilities that entail relational inference—such as navigation [13–15], learning-to-learn [9, 16, 17], and concept/structure learning [11, 12, 18]—are in fact widespread across the animal kingdom. Further, these abilities have been linked to memory systems in the brain—variously termed “relational memory”, “cognitive maps”, “learning sets”, among others—that enable humans and animals alike to make systematic inferences [5, 19–21], generalize across different domains [16, 22, 23], learn rapidly [24–28], and plan and envision new experiences [29–36]. These findings and insights extend the scope of relational inference to a wide range of species and cognitive abilities, and, further, imply that there exists a deep interrelationship between relational inference and memory.

Despite this unifying importance, it remains an open question how relational inference is implemented in neural systems, whether in artificial networks (e.g. those performing linguistic [37–39] or symbolic [40, 41] tasks) or in the brain. Toward answering this question, a fundamental scientific aim is to identify or generate putative neural implementations that can be used to derive empirically testable hypotheses. In particular, hypotheses at the level of behavior and of collective (population-level) neural activity may be crucial given that these levels have proved decisive in clarifying whether and how neural systems in the brain implement various cognitive functions (e.g. vision, movement, timing, decision-making [42–46]). Notably, in both neurobiology and machine intelligence, relational inference is often studied in relatively complex cases (e.g. spatial [16, 21, 24, 47–49] and linguistic [37, 38, 50] knowledge), for which it may be relatively difficult to formulate or generate hypotheses derived from neural implementations, especially at the level of neural populations and of behavior. In this way, studying simpler cases of relational inference may be advantageous or even crucial towards understanding relational inference in the brain. We therefore took a two-part approach intended to yield such hypotheses: first, we stipulated a task paradigm that distills relational inference into a simple yet essential form, and second, we adopted a methodology suited to discover possible population-level and behaviorally relevant neural implementations thereof.

## Results

### Transitive inference: A classic cognitive task

We first sought to operationalize relational inference in a task that is (i) reduced to a single abstract relation, and, further, (ii) implementable with stimuli (e.g. images) that can be presented with temporal precision. We reasoned that each of these properties might be critically important in that (i) reduces complexity, which could ultimately enable discovering neural implementations, and (ii) delimits periods of sensory-driven neural activity, thus facilitating subsequent interpretation of neural activity.

**Fig 1:**
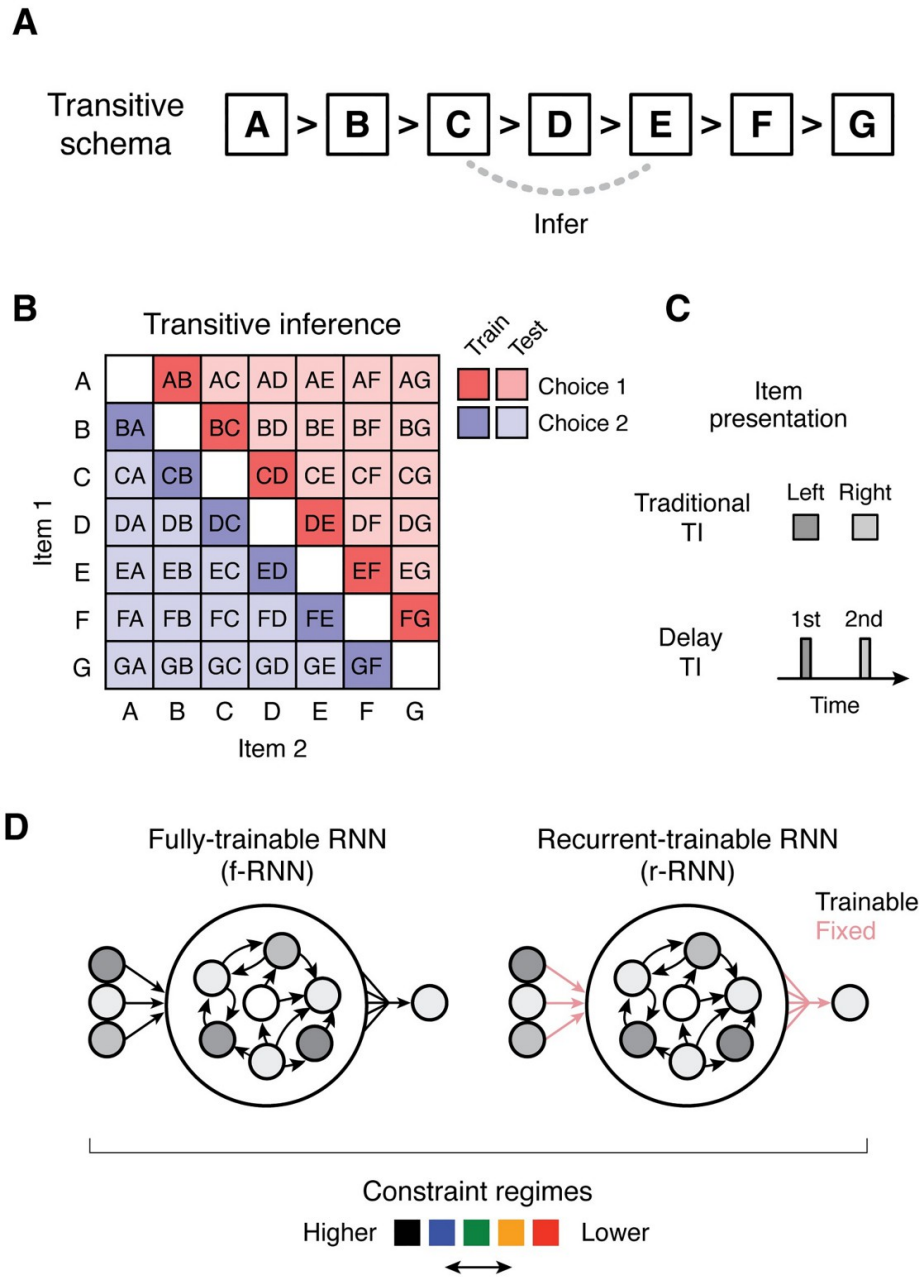
Cognitive task and neural approach. **A**, Diagram of the relational structure (schema) underlying transitive inference (TI). Subjects learn correct responses to premise pairs (A vs. B, B vs. C, C. vs. D, etc.; training trials), and must infer correct responses in previously unobserved (novel) pairs (C vs. E, etc.; testing trials). For every possible pair, the “higher” item should be chosen. Items (A, B, etc.) can correspond to arbitrary stimuli. **B**, Trial types and their correct responses in 7-item TI. Each trial type consists of a permutation of two items (item 1 and item 2). **C**, Item presentation formats: traditional TI vs. delay TI. In traditional TI, items are presented simultaneously and are chosen on the basis of their presented position (e.g. left vs. right). The present study proposes “delay TI”, in which items are presented with an intervening delay and are chosen on the basis of presentation order (1st vs. 2nd). This task format explicitly requires working memory (WM). **D**, Neural models in the present study. Among neural network (NN) architectures, recurrent neural networks (RNNs) are able to implement WM. Two variants of RNNs were studied: fully-trainable RNNs (f-RNNs), for which both feedforward and recurrent synaptic weights were modifiable in training, and recurrent-trainable RNNs (r-RNNs), for which only recurrent synaptic weights were modifiable in training (feedforward weights randomly generated and fixed; trainable vs. fixed weights diagramed as black vs. pink arrows, respectively). In conjunction, RNNs were trained with different levels of regularization and initial synaptic strengths (‘constraint regime’, indicated with colors shown; parameters in Table 1). All networks had 100 recurrent units, which all had the same non-linear activation function (tanh). See Methods for additional details.

A classic task paradigm capturing these properties is **transitive inference**(**TI**) [10, 52, 57, 83, 84], which tests a subject’s ability to use premises A > B and B > C to infer A > C, i.e. to respond on the basis of an underlying relation (choose ‘higher’ items, based on the transitive ‘>’ relation). TI operationalizes a simple yet powerful schema (Fig 1A) that enables generalization from N premises (training cases) to order *N*^2^ probes (test cases) in accordance with a relation-based rule (i.e. choose the ‘higher’ item). In contrast to generalization based on interpolation or extrapolation, TI tests generalization that is expressly compositional and based on an underlying schema (‘schematization’), manifesting behaviorally in the pattern of correct responses in the task (Fig 1B). Transitivity (‘>’) is itself characteristic of many types of relations, and is of fundamental importance in symbolic reasoning—together suggesting that TI is not essentially based on specific stimuli or stimulus features in isolation.

At a broader level, TI can be understood as a simplified alternative to other paradigms testing relational inference but involving multiple relations and rules (e.g. linguistic syntax and semantics) and/or task stimuli that are relatively challenging to isolate (e.g. spatial paradigms [16, 21, 24, 47]). Further, in contrast to approaches that focus mainly on lower-level neurobiological phenomena (e.g. neural firing that has abstract correlates [14, 22, 85]), TI requires direct behavioral report of successful inference, thus affording identification of potentially important relationships between behavior and underlying neural implementations.

Remarkably, though TI is a classic task in behavioral psychology [10, 52, 83, 86, 87] and a cornerstone of symbolic reasoning, the neural basis of TI remains unclear [52, 55, 88, 89], potentially due to a lack of putative neural implementations and testable hypotheses.

### A neural approach to TI

Despite the long history of TI as a cognitive task, investigation of its neural basis in the brain is comparatively recent [57, 90–94]. Prior work implies that an approach seeking to identify biologically accurate neural implementations would benefit from two criteria:

First, an approach that imposes only **minimal architectural constraints**. TI is observed in an extremely broad range of species, including primates, birds, rodents, and insects [52, 55, 89, 95–97] (possibly the result of convergent evolution [98]), a striking ubiquity that implies that highly specialized neural architecture may not be essential.

Second, an approach that explicitly requires memory across time, particularly **working memory (WM)** [99–102]. In living subjects, relational inferences such as TI typically rely on memory since subjects must assess relationships between events not experienced simultaneously (e.g. sensory stimuli separated in time)—memory enables such events to be in effect brought together. WM in particular is thought to be essential to relational inference [53, 54, 103, 104], not only because WM is generally required in real-world cases of relational inference (e.g. language comprehension, spatial navigation), but also because prior work suggests that relational inference is accomplished in the brain by a neural system that intrinsically supports and/or relies upon WM (e.g. prefrontal cortex, possibly via a process akin to deliberation or reasoning [99, 101, 105]). Surprisingly, though TI exemplifies relational inference, prior work has only evaluated indirect relationships between TI and WM (either by having separate WM vs. TI tasks [106], or by linking each to a common brain region [90, 107]), with almost no study directly testing WM by imposing an intervening delay between presented items, a task format we here call “delay TI” (item 1—delay—item 2; Fig 1C and S1A Fig; see [108] which imposes a WM delay in a probabilistic version of TI). Notably, previously implemented tests of TI often have items in separate environmental locations or containers, thus implicitly requiring WM (e.g. [57, 96, 97]).

Importantly, these two criteria are suited for the methodology of generating and analyzing task-trained **recurrent neural networks** (**RNNs**), an approach that has been successful in discovering neural implementations of other cognitive abilities, and, further, in generating testable predictions at the level of collective neural activity [80, 109, 110]. We therefore adopted this approach, and, further, expanded upon it in two ways. First, in conjunction with RNNs, we also assessed whether and how trained models that cannot implement WM, yet have neurally relevant feedforward structure, might transitively generalize. To do so, we investigated two archetypal models: logistic regression (LR [111]) and multi-layer perceptron (MLP [112]) (schematic in S2A Fig), each tested on the “traditional TI” format having no delay (Fig 1C). Second, to identify (where possible) multiple solutions to TI, we investigated two neurobiologically relevant types of RNN variants:

***Learnable connectivity.*** Neural architecture in the brain is comprised of feedforward and recurrent connectivity, for which respective roles in learning cognitive tasks remain generally unclear (e.g. [113–115]). Indeed this is the case for TI, a task paradigm that entails learning correct responses to otherwise arbitrary stimuli (items A, B, C, etc)—it is not known whether the learned connectivity is feedforward, recurrent, or some configuration of both. Distinguishing between these possibilities is fundamentally important since they correspond to different kinds of neural substrates and solutions. Moreover, the question of learned connectivity is particularly important to relational inference tasks, since in these tasks the significance of stimuli (i.e. the arbitrary items A, B, C, etc.) has, by design, no *a priori* relationship to stimulus features (unlike tasks that are based on stimulus features known to be encoded in feedforward inputs from upstream sensory brain regions, e.g. tactile frequency [116, 117], visual frequency and orientation [118], object categories [119]).

We therefore trained RNN variants having different configurations of trainable connectivity: namely, either having both feedforward and recurrent connectivity be trainable—which we termed “fully-trainable” RNNs (**f-RNNs**)—or having only recurrent connectivity be trainable—which we termed “recurrent-trainable” RNNs (**r-RNNs**) (Fig 1D).

***Constraint regime.*** The accuracy of trained NNs in matching experimentally recorded neural responses has been found to depend on efficiency constraints; these constraints are implemented as training penalties (regularization) that limit excess neural activity and/or the strength of connectivity [48, 67, 68, 78, 115, 120]. Previous studies have also found that differences in the initial strength of connectivity (the magnitude of connection weights prior to training [121, 122]; a “soft” constraint) yield trained NNs that have substantial differences in internal representation and neurobiological accuracy [48, 67, 68, 72, 123]. Yet beyond the tasks studied in this prior work, it remains generally unknown whether and in what ways these two types of constraints—efficiency (regularization) and initial connectivity strength—yield NNs having different task solutions or degrees of neurobiological accuracy.

We therefore trained RNN variants using hyperparameter sets that varied both factors, referring to each hyperparameter set as a “constraint regime”. We defined five regimes, which we termed “highest”, “high”, “intermediate”, “low”, and “lowest”, where the hyperparameter values between regimes enabled comparing either efficiency or initial connectivity strength (Table 1; for example, “highest” vs. “high” regimes differ only in regularization; further explanation in Methods); hyperparameter values were also chosen to be similar to that in previous work [48, 67, 72].

**Table 1:** Constraint regime parameters. *h*_0_, input gain; *g*_0_, recurrent gain; *α*, weight regularization; *β*, metabolic regularization.

	*h*_0_	*g*_0_	*α*	*β*
Highest	1.0	0.5	1.0	1.0
High	1.0	0.5	0.01	0.01
Intermediate	1.0	1.0	0.01	0.01
Low	1.0	2.0	0	0
Lowest	1.5	4.0	0	0

### A variety of neural models perform TI

We first sought to determine whether trained RNNs could successfully perform TI. Unlike perceptual tasks or tasks that solely test memory, TI expressly requires generalization to novel combinations of inputs. Such generalization requires some form of additional knowledge regarding the underlying relationship between inputs (the transitive schema, Fig 1A). Thus TI is a task that requires an *a priori* inductive bias—here, for transitivity—the implementation of which is not generally known in relatively unstructured models such as trained RNNs [80, 124, 125].

Mirroring TI as presented to living subjects (7-item TI with items A to G; each item represented as a random high-dimensional (100-D) input), we trained RNNs (100 recurrent units, tanh nonlinearity) exclusively on premise (training) trials (consisting of ‘adjacent’ item pairs A vs. B, B vs. C, C vs. D, etc.) and evaluated whether RNNs generalized to test trials (B vs. D, etc.; all trial types shown in Fig 1B). Training was conducted using gradient descent optimization and backpropagation-through-time; further, all trials required working memory (WM) to relate items separated by a stimulus-free delay (delay TI; Fig 1C and S1 Fig; delay lasting 2 to 6 unit-level time constants (*τ*) and either of fixed or variable length, see Methods). Response choice was defined by which of two output units (linear readouts corresponding to choice 1 vs. 2) met a fixed activity threshold (85% of maximum value), with response time (RT) defined as the time this threshold was reached. For an initial assessment of whether the networks could generalize, a simulation of all trial types was performed under noiseless conditions.

**Fig 2:**
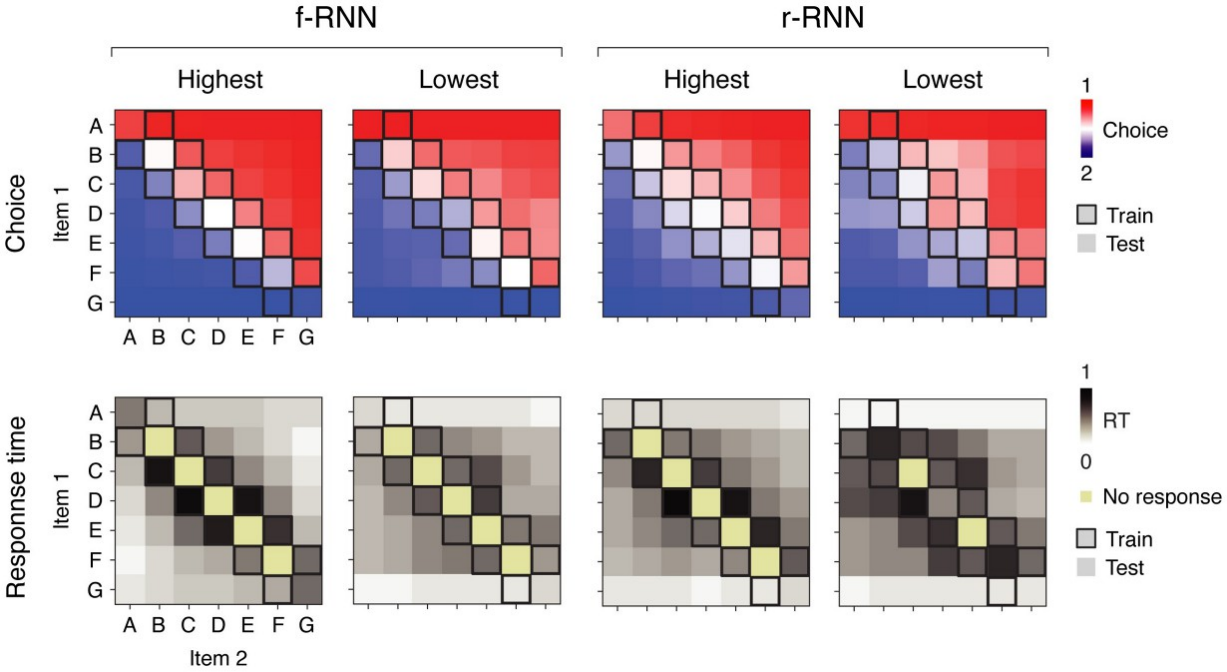
A collection of recurrent neural networks (RNNs) perform transitive inference (TI). Four example RNNs that perform delay TI. Each example RNN is plotted in a column. At left are fully-trainable RNNs (f-RNNs); at right are recurrent-trainable RNNs (r-RNNs); for each, one RNN from each of two constraint regimes (highest and lowest) are plotted. Top row, network output (choice) by trial type. Plotted for each trial type (in squares, defined by items 1 and 2; e.g. AB, BA, AC) is the value of output unit 1 minus that of output unit 2, averaged across the second half of the choice period (Choice, red and blue shades, corresponding to choice 1 and 2, respectively; compare to Fig 1B). Note RNNs were trained (optimized) only on training trial types (boxed squares). Bottom row, response time (RT) by trial type. In each trial, the choice response of the RNN was defined by the identity of the output unit (linear readout; one for each of two choices) that was first to reach a fixed activity threshold (85%); RT was defined as the time taken for the output unit to reach fixed activity threshold (85%) in the choice period (see S1A Fig), measured as a proportion of the full duration of choice period.

We found that trained RNNs often generalized perfectly, i.e. responded correctly to all test trials (example RNNs in Fig 2; summary in Table 2; additional RNNs trained on extended and variable delays in S1 Table and S3C Fig). Interestingly, it was also common for trained RNNs to fail to generalize despite responding correctly on all training trials (examples in S3A Fig), whereas feedforward models trained on the traditional task format never failed to generalize (examples of outputs in S2B Fig; LR: 100 out of 100 instances; MLP: 100 out of 100 instances; see also [56, 62] for additional results in MLPs).

**Table 2:** Number of RNNs that fully generalized out of 200 trained instances. Each entry corresponds to a different RNN variant (columns: learnable connectivity (f-RNN or r-RNN); rows: constraint regime (higher to lower), see main text for explanation and Table 1 for parameter values). All trained instances responded correctly on all training trials, regardless of generalization performance.

	f-RNN	r-RNN
Highest	200	200
High	200	181
Intermediate	198	165
Low	192	110
Lowest	162	65

Notably, we also observed that, among RNN variants, fully-trainable RNNs (f-RNN) and higher-constraint regimes more frequently yielded RNNs that generalized (Tables 1 and 3), providing an initial hint that RNN variants might have functionally important differences.

**Table 3:** Main predictions of representative models. ••, a prediction unique to this model. •, two predictions that jointly are unique to this model. “End order” effect: Fig 4. Collinearity: Fig 7A. Mean angle change: Fig 7B. Oscillation in delay period: Fig 5C and S6 and S7A–S7C Figs. Choice axis encoding: Fig 7C. All neural predictions refer to the top PCs of population activity.

	Behavior	Neural activity				Implication
	“End order” effect	Collinearity	Mean angle change	Oscillation in delay period	Choice axis encoding	
f-RNN highest	••2nd-faster	•Early: >0.5 •Late: >0.5 Change: >0	••<0	∼0.5 cycles/delay Choice aligned XCM orthogonal	••2nd-dominant	Feedforward input learned, “subtractive” solution
f-RNN lowest	1st-faster	Early: >random Late: >random ••Change: <0	>0	n/a	1st-dominant	Feedforward input learned
r-RNN highest	1st-faster	•Early: random •Late: >0.5 Change: >0	>0	∼0.5 cycles/delay Choice aligned XCM orthogonal	1st-dominant	Recurrent dynamics learned, single oscillation sufficient
r-RNN lowest	1st-faster	•Early: random •Late: <0.5 Change: >0	>0	n/a	1st-dominant	Recurrent dynamics learned

### RNNs performing TI show multiple emergent behaviors

Decades of work have established that subjects performing TI widely show striking patterns of behavior, manifesting both in performance and response times (RT) [52, 55]. These behaviors are based on trial type, with each trial type defined by items (e.g. AB, BC, AC) and where each item is defined by its position, or “rank”, in the transitive schema (Fig 1A; rank of A is highest, while G is lowest). As recognized previously [55], these empirically observed behaviors are not only important constraints on explanatory accounts of TI, but also potential sources of insight into underlying implementations.

**Fig 3:**
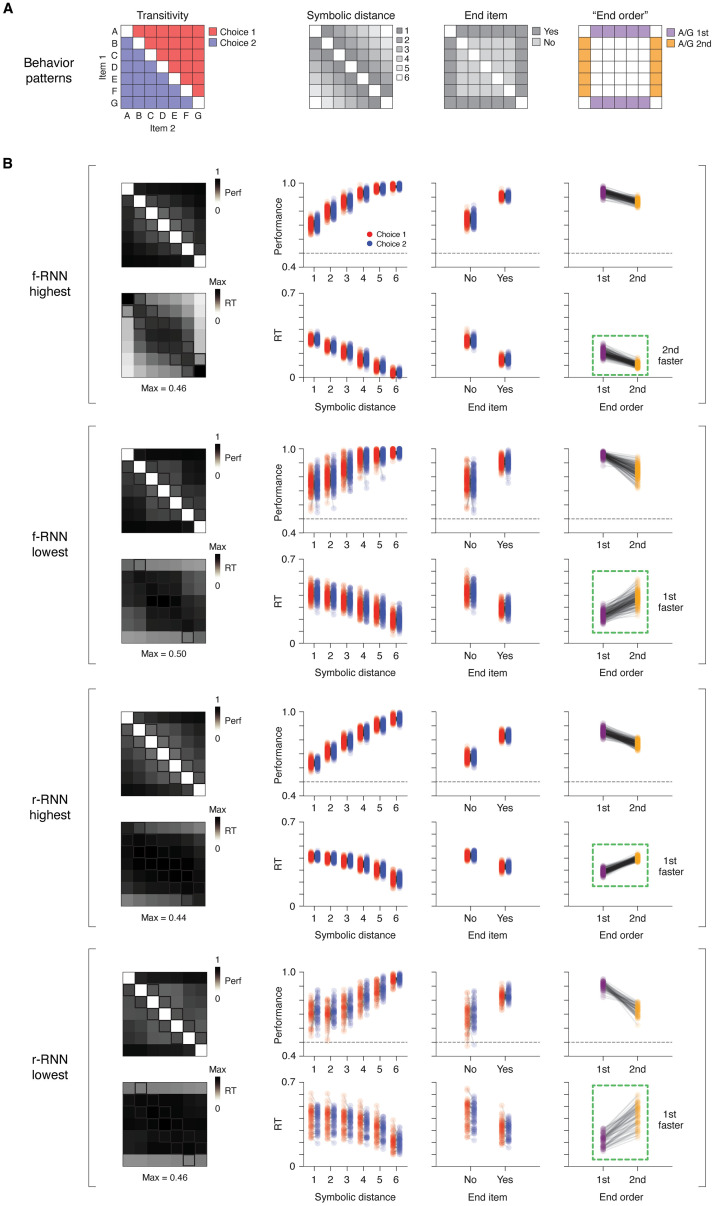
RNNs performing TI show multiple emergent (untrained) behaviors. **A**, Schematic of behavior patterns. Each schematic shows the trial types (squares; specified by item 1 and 2) defining the behavioral pattern. **Transitivity**: the correct choice (item 1 or 2) for transitive inference (i.e. the item ‘higher’ on a transitive schema, Fig 1A). **Symbolic distance**: the size of the difference in rank between item 1 and 2; rank is an item’s discrete position in the transitive schema (A: 1, B: 2, C: 3, etc.). **End item**: whether the trial type contains an item at either extreme of the transitive schema (‘end item’; here A or G). **End order**: whether the end item (A or G) is the 1st item (item 1) or 2nd item (item 2) presented in the trial; defined only for delay TI (see Fig 1C and S1A Fig). **B**, Behavioral results across four RNN variants (n = 65–200 instances / variant; see Table 2 for counts). The four RNN variants were defined by different learnable connectivity (f-RNN vs. r-RNN) and constraint regime (highest and lowest). Results from each RNN variant are presented in a block of two rows (top row: average performance (proportion correct); bottom row: average RT (proportion of the duration of the choice period; in trial-type matrix (leftmost plot), values are normalized to the maximum value observed across trial types (Max, value reported at bottom)). Column 1: Averages across RNN instances by trial type. Columns 2–4: Averages across trials by trial type, for all RNN instances (500 simulated trials / trial type; each point corresponds to an RNN instance). Trial types follow those in panel A (column 2: symbolic distance; column 3: end item; column 4: “end order”) and distinguish between choice 1 vs. choice 2 trial types (red vs. blue, respectively; diagramed in panel A). Two versions of the “end order” pattern are highlighted (1st- vs. 2nd-faster; dotted green boxes).

We therefore investigated whether these behaviors (described below), and possibly others, were expressed by RNNs that successfully performed TI (Fig 3). Importantly, expression of these behaviors would effectively be emergent, since the RNNs were neither pre-configured nor trained to express particular behaviors beyond that of responding correctly on training trials. Further, prior work on TI has focused on the traditional TI format (Fig 1C; lacking a WM delay), leaving unclear whether there exist behavioral patterns that stem from imposing a WM delay.

Given these aims, we took the following approach to characterize task behavior across RNNs. First, we focused on RNNs that generalized fully (correct responses for every test trial type). Next, for each individual RNN, we simulated the networks with progressively increasing levels of intrinsic noise until the average performance of the RNN was >50% on training trials and <96% on testing trials (i.e. sub-asymptotic performance, the level of performance for which behavioral patterns have been observed). Lastly, we ran simulations (500 runs, all trial types) at this noise level, from which we then measured performance (% correct) and RTs. RTs were measured using a standard criterion (time to a fixed threshold in output units [66]).

We found that RNNs exhibited not only previously established behavioral patterns, but also a novel behavioral pattern not previously studied (Fig 3; additional RNN variants in S4A Fig; analogous analysis in feedforward models in S2C and S2D Fig). Importantly, behavior was qualitatively and quantitatively comparable to that of living subjects (S5 Fig). We address each behavioral pattern in turn.

#### The symbolic distance effect

A standard observation across behavioral studies of TI is the “symbolic distance” effect: the larger the difference in rank between items (e.g. AD vs. AB), the higher performance and lower RT [52, 55, 83, 89]. We found that RNNs performing TI invariably exhibited the symbolic distance effect (Fig 3, second column; see also S5 Fig).

#### The end item effect

Along with the symbolic distance effect, a standard behavioral observation in subjects is the “end item” (or “terminal item”) effect: trials containing either the highest- or lowest-rank item (“end items”; here A and G) are associated with higher performance and lower RTs [52, 55, 126]. We found that RNNs performing TI invariably exhibited the end item effect (Fig 3, third column; see also S5 Fig).

#### The “end order” effect: A novel behavior with two distinct versions

 Traditional TI presents items without an explicit intervening delay, prompting subjects to choose on the basis of item position (e.g. left vs. right designated as item 1 vs. 2, respectively; Fig 1B). In contrast, delay TI prompts subjects to choose on the basis of item order, i.e. whether an item is presented 1st vs. 2nd (before vs. after delay, designated item 1 vs. 2, respectively). In a subject performing delay TI, this difference in task paradigm may incur an order-dependence in how items are evaluated, i.e. XY and YX trials may differ, neurally and/or behaviorally (apart from their different correspondent correct responses). Any such order-dependent effect would be observable in a matrix of trial types (i.e. items 1 and 2) as an asymmetry across the main diagonal.

Unexpectedly, we observed an order-dependent effect that was widespread and also markedly varied across RNNs: in trials containing an end item (A or G), response times (RTs) were often highly order-dependent (e.g. RTs were faster in AX trials than XA trials). This effect was apparent as an asymmetry in trial-type matrices of RTs (‘stripes’ in the first/last column or first/last row), both in individual RNNs (e.g. Fig 2, bottom row) and in averages across RNNs for a given variant (Fig 3B, first column).

Strikingly, the pattern occurred in two qualitatively different versions. Some RNNs showed lower RTs if an end item (A or G) was presented first rather than second (Fig 3, fourth column, f-RNN highest; also Fig 2, first example), while other RNNs showed the opposite behavioral pattern, namely, lower RTs if an end item (A or G) was presented 2nd (rather than 1st) (Fig 3, fourth column, f-RNN lowest, and both r-RNN highest and r-RNN lowest; also Fig 2, second through fourth examples).

**Fig 4:**
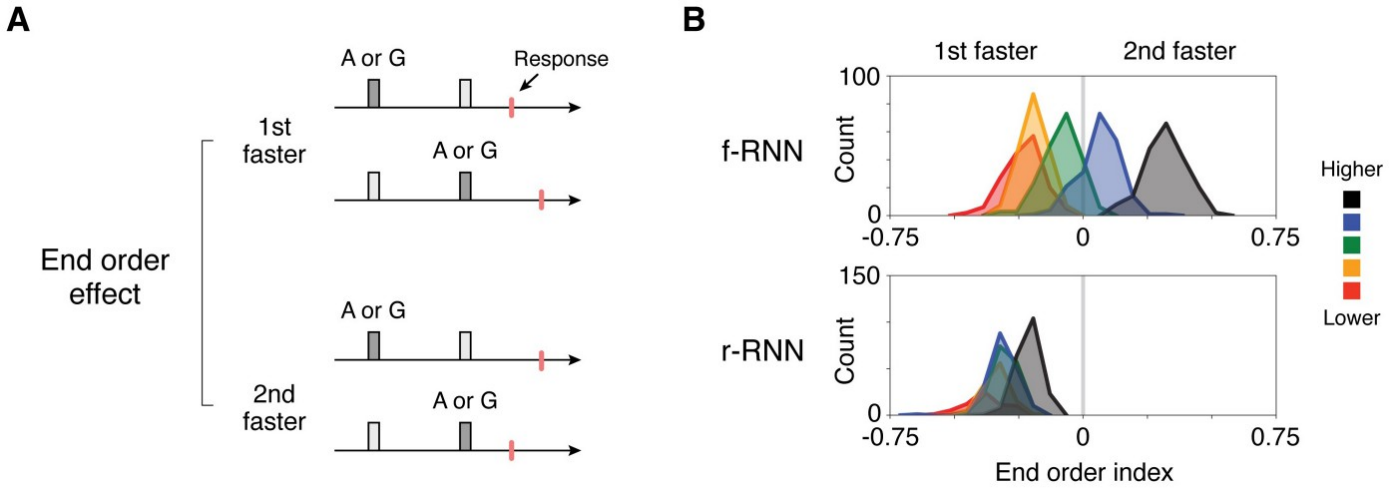
RNNs show a novel order-dependent behavior having two distinct versions. **A**, Schematic of the end order effect, a response-time (RT) behavioral pattern. The behavioral pattern is observed in RNNs in two qualitatively different versions: 1st-faster vs. 2nd-faster. A schematic showing the trial types used to quantify the effect is in Fig 3A (fourth column). **B**, Histograms of end-order behavior across RNNs (counts: RNN instances). The behavior was quantified as the difference of RTs divided by their sum (end order index; RTs calculated for trials where end items (A and G) occurred either 1st vs. 2nd). RNN variants (f-RNN vs. r-RNN and higher to lower constraint regimes) follow that diagramed in Fig 1D. These results summarize those in the last column of Fig 3.

We termed the basic pattern the “end order” effect (“end-item order”; quantified separately in the last column of Fig 3), and its former and latter manifestations as “1st-faster” and “2nd-faster” versions, respectively. In examining this behavior across networks using a quantitative index (end order index, ranging from -1 (1st-faster) to 1 (2nd-faster); see Methods) (Fig 4, additional RNN variants in S4B Fig), we found that the two versions of the behavior systematically differed with respect to both types of RNN variants, whether learnable connectivity (f-RNN vs. r-RNN) or constraint regime (higher to lower). First, we found that r-RNNs virtually always showed the 1st-faster pattern (Fig 4B, bottom row), whereas f-RNNs showed both 1st- and 2nd-faster patterns (Fig 4B, top row). Second, we found that lowest-constraint RNNs nearly always showed the 1st-faster pattern (Fig 4B, red histograms in top and bottom rows). In contrast, higher-constraint f-RNNs consistently showed the 2nd-faster pattern (Fig 4B, both highest and high regimes, black and blue histograms, respectively, in top row).

The finding that RNNs performing TI show qualitatively different patterns of behavior (Fig 3, fourth column, Fig 4) furthermore suggested that the networks expressed different underlying neural solutions, motivating direct investigation.

### A simple neural solution to delay TI

Neural recordings in living subjects performing TI indicate that single neurons in the brain can encode variables relevant to TI, including symbolic distance [91, 92]. It remains unclear what collective neural process or activity pattern implements the comparison operation—akin to a ‘>’ operator—that generalizes transitivity to novel combinations of items.

For initial insight, we analyzed how purely feedforward models generalized transitively when WM was not required (logistic regressions and multi-layer perceptrons trained on the traditional TI task format, S2 Fig). Examination of unit activations in these models indicated that transitive comparison was implemented using a “subtractive” solution, where the rank (A, B, C, etc.) and position (left vs. right) of each item was mapped to the magnitude and sign, respectively, of unit activation (S2E Fig). It was further notable that in either feedforward model, this solution was sufficiently realized by (trained) feedforward weights operating directly on the input [56], clarifying that a direct operation on otherwise arbitrary inputs (items A, B, C, etc. encoded as high-dimensional (100-D) random vectors in input space) is sufficient to perform TI when feedforward input can be learned. This raised the question of how TI is performed when modifiable feedforward connectivity is not sufficient (e.g. when WM is required) and/or not available (e.g. r-RNNs or a putative neural system in the brain responsible for performing the task).

**Fig 5:**
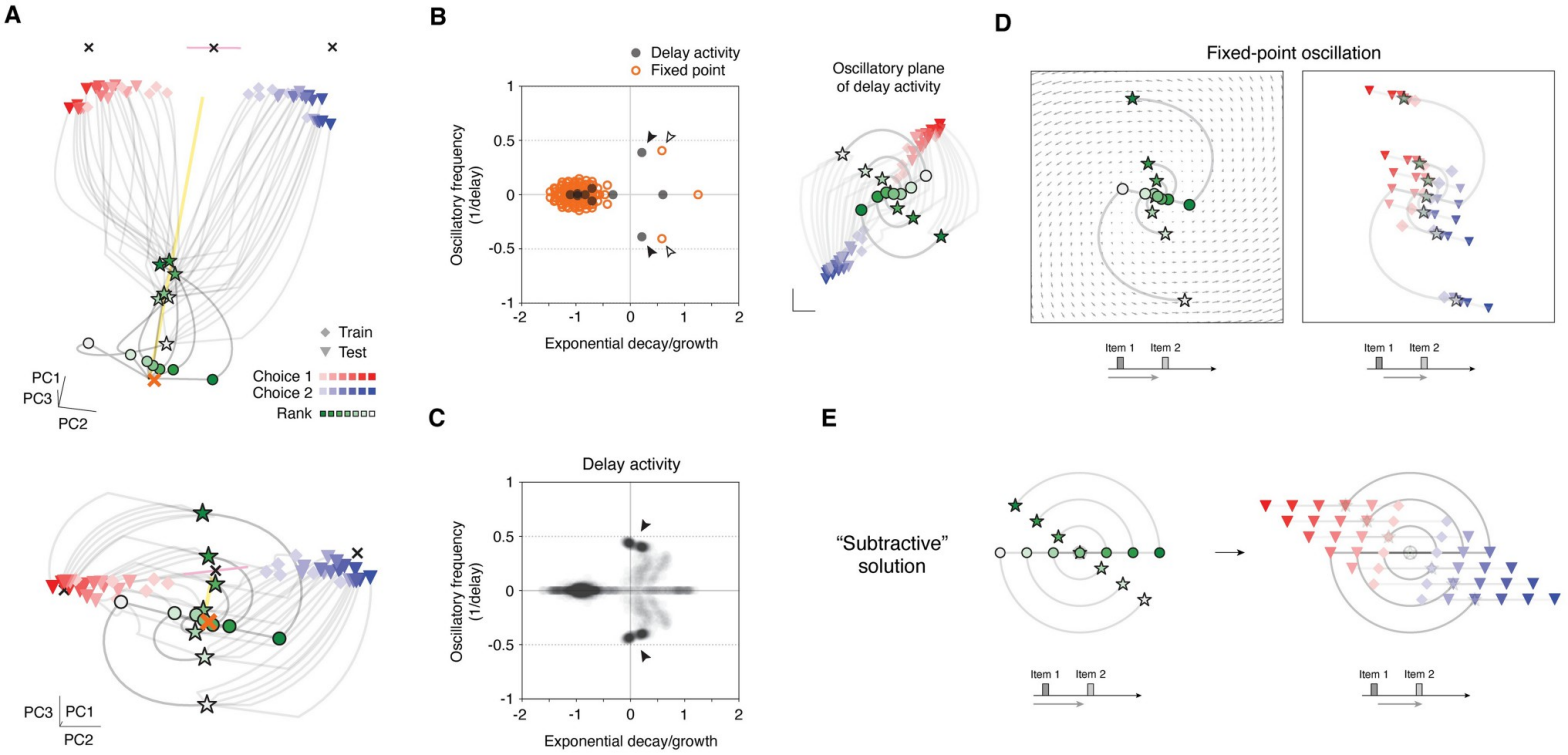
A simple neural solution to delay TI. **A**, Population activity trajectories in an example RNN (high-constraint f-RNN) that performs TI. Top and bottom plots show two different views. Shown are trajectories from all 42 trial types (see Fig 1B). To clarify the operation of the network, three trial times are highlighted: (i) presentation of item 1 (green circles; shade indicating item rank: A (dark green) to G (white)), (ii) the last time point of the delay period (green stars; same color convention), (iii) last time point of the trial (red/blue symbols; red: choice 1 trials, blue: choice 2 trials, light to dark shading indicating symbolic distance (1 to 6); diamonds: training trials, triangles: test trials). Also shown: cross-condition mean (XCM; the average trajectory across all trial types) (yellow line) and fixed points (FPs) (crosses). Two FPs were attractors (black crosses), one FP was a saddle point (black cross with pink line indicating axis of unstable mode), and one FP (orange cross) was located near trajectories during the delay period (‘early-trial’ FP). Note two prominent activity patterns: linearly arranged rank-ordered activity upon presentation of item 1 (green circles) and the oscillatory evolution of trajectories in the delay period (circles to stars). **B**, Linear dynamics of RNN in panel A. Left, eigenvalue spectra of the RNN. The spectra were calculated in two ways: first, from delay-period neural activity (black points; inferred via least-squares linear fit, *R*^2^ = 0.65) and second, from linearization of the network with respect to the early-trial FP (orange circles; FP shown as orange cross in panel A). Right, population activity trajectories of the RNN plotted in the plane of the oscillation inferred from delay period activity (filled arrowheads in spectra plot). **C**, Linear dynamics of higher-constraint f-RNNs (n = 400 instances; 200 highest and 200 high). Eigenvalue spectra of delay-period neural activity (grey translucent points; inferred via least-squares linear fit, *R*^2^ ∼0.6–0.9; see S6A Fig). Note the density of oscillatory modes with frequency ∼0.5 cycles / delay (filled arrowhead). **D**, Activity trajectories in the oscillatory mode of the linearized RNN. The oscillatory mode is that of the linearization of the early-trial FP (open arrowheads in panel B). Plotted in background are flow field vectors (not to scale; shown to indicate motion direction). To clarify how the activity evolves, trajectories are plotted for two successive trial periods (left and right panels; schematic at bottom of each): early trial (left) and presentation of item 2 (right). Three trial times are highlighted: (i) presentation of item 1 (circles; color indicating item rank: A (dark green) to G (white)), (ii) the last time point of the delay period (stars; same color convention), (iii) presentation of item 2 (red/blue symbols; red: choice 1 trials, blue: choice 2 trials; diamonds: training trials, triangles: test trials). Activity states for (iii) solely reflect the application of (feedforward) item 2 input. Note the separation of choice 1 vs. 2 trials (red vs. blue symbols), indicating that correct responses were evolved in the activity space. **E**, Diagram of solution expressed in networks: population-level “subtraction”. Plotted are activity trajectories generated by simulating a 2D linear dynamical system defined by an oscillation of frequency ∼0.5 cycles / delay, with initial condition at the origin and input vectors encoding task items (A, B, C, etc.) in ordered collinear arrangement in state space (compare to panels a and d). Trial-based input (item 1—delay—item 2, see S1A Fig) was applied to the system. Plotting conventions are the same as in panel D. For further detail of the solution, see S1 Appendix.

We therefore sought to clarify neural implementations in RNNs performing delay TI. We began by investigating fully-trainable RNNs (f-RNNs) trained in the higher constraint regimes (Fig 5; examples trained on extended and variable delay formats in S4C and S4D Fig), as we thought these networks might be the most similar to the purely feedforward models (and thus most tractable to analyze), and since these networks are most commonly adopted RNN variant in modeling neural systems in the brain.

We found that population activity in these networks was consistently low-dimensional (variance explained by top 3 PCs: 98.1% ± 0.4 (highest) and 97.1 ± 1.4 (high), mean ± s.d., 200 instances each; S3B Fig). Next, in examining activity trajectories (population activity across time) (Fig 5A), we observed two patterns: (1) **a linearly-arranged rank-ordered** response to item presentation (Fig 5A, green-shaded circles, corresponding to the network response to item 1 presentation), and (2) a prominent **rotation** during the delay period (Fig 5A, bottom), suggesting an oscillatory dynamic.

Pattern 1 suggested a similarity to the subtractive solution, as the pattern was characterized by an intrinsically 1D structure across trial types (observed in single units in feedforward models, whereas here in population activity space) and was due to trained feedforward connectivity.

Pattern 2 suggested that a low-dimensional dynamical process—potentially a single (2D) oscillation—could account for how these networks implemented transitive comparison across time. Indeed, we found that RNN activity during the delay period was effectively described by linear dynamics (ordinary least-squares fit) consistently characterized by an oscillatory mode of frequency ∼0.5 cycles/delay and correspondent with pattern 2 (Fig 5B and 5C, highlighted with filled arrowhead; *R*^2^ ∼0.6–0.9 in higher-constraint f-RNNs, S6A Fig, first row, left). This oscillatory mode moreover appeared characteristic of relatively higher constraint f-RNNs, as lowest-constraint f-RNNs did not predominantly express this mode (S6A Fig, first row; see also S6B Fig for networks trained on extended and variable delay formats, showing a similar difference). For additional comparison, we also assessed RNNs trained on delay TI while being constrained to modify only their feedforward weights (“feedforward-trainable” RNNs (ff-RNN), see Methods); these networks were able to perform TI (S1 Table for number of instances), yet, unlike f-RNNs, lacked the characteristic oscillatory mode (S6A Fig, second row).

Given these clues, we then sought to identify the underlying solution explicitly. Importantly, prior work has found that analyzing population-level neural dynamics in trained RNNs with respect to fixed points (FPs) can identify dynamical components that have specific task functions [77, 79, 80, 127] and that are jointly sufficient to perform cognitive tasks.

Taking this approach, we found that higher-constraint f-RNNs had a fixed point near activity trajectories at the beginning of trials, which we refer to as an “early-trial” fixed point (Fig 5A, orange cross). Linearization analysis revealed that this FP had an oscillatory mode of frequency ∼0.5 cycles/delay (highlighted by open arrowheads in Fig 5B). In activity space, this oscillatory mode was orthogonal to the mean trajectory across trial types (i.e. the cross-condition mean (XCM), a population activity component consistently observed in trial-based tasks [74–76]; plotted as yellow line in Fig 5A; analysis of orthogonality between oscillation and XCM in S7A and S7B Fig).

In some cases, higher-constraint RNNs also showed additional FPs that were associated with other task functions (Fig 5A, black crosses): “choice” FPs that were stable FPs (attractors) toward which trajectories corresponding to each of the two choices travelled, and a saddle FP, located between the choice FPs, that was stable except for a single unstable axis oriented toward each choice FP (Fig 5A, choice FPs: black crosses near end of choice 1 and choice 2 trajectories; saddle FP: center black cross, unstable axis in pink line; stability of FPs determined by linearization analysis, not shown). These dynamical components suggest that such networks implement a simple binary dynamical ‘readout’ of choice following item 2 presentation (in the choice period), consistent with the activity trajectories visualized during this time period (Fig 5A, seen as the lack of re-arrangement between choice 1 vs. choice 2 activity trajectories, which correspond to trajectories ending with red vs. blue symbols, respectively; see also S3D Fig for output/readout activity in example networks).

Taken together, the above dynamical components suggested that a comparison (between items 1 and 2) enabling transitive generalization could be performed within the 2D subspace (plane) of an appropriately aligned oscillatory mode, such as that associated with the early-trial FP. To test this possibility, we evaluated whether TI could be performed solely by activity and dynamics within the subspace of the oscillatory mode (constituting a linear approximation of the oscillation seen in the RNN; see Methods for identification procedure). We found that this oscillation, when presented with trial-structured input (item 1—delay—item 2), yielded activity trajectories for which correct choice was linearly separable in activity space (Fig 5D).

This clarified the implementation of transitive comparison over time in these networks (Fig 5E): namely, a single oscillation that re-orients the activity states encoding item 1 (A, B, C, etc.), doing so via a common angular displacement. This re-orientation serves to shift activity states in the direction *opposite* to the direction of activity displacement due to item presentation. Note that the activity displacement due to item presentation is rectilinear—the consequence of its implementation as feedforward input—whereas the dynamics-driven activity displacement in the delay period is angular. The re-oriented activity state is thereby “subtractive” with respect to the activity shift subsequently elicited by the presentation of item 2.

An angular shift of ∼0.5 cycles (resulting from an oscillatory frequency of ∼0.5 cycles/delay) re-orients the activity state to be opposite (diametric) to that imposed by the presentation of the item prior to the delay. This can be likened to a change-of-sign along a “subtraction” axis in population activity space (Fig 5E, horizontal axis), and is analogous to the mapping of signs (- vs. +) to item position (left vs. right) in the subtractive solution seen in the feedforward models (S2E and S2F Fig); this solution can be thought of as subtraction at the population level (Fig 5E and S1 Appendix). The resulting activity along the subtraction axis can account for transitive comparison and the symbolic distance effect, as long as the network output (readout weights of output units) is aligned with the subtraction axis. This predicts that the oscillatory mode is aligned to the choice axis (the direction in activity space from choice 1 to choice 2 trials; see Methods), which we found consistently to be the case in relatively higher constraint f-RNNs, as compared to relatively lower constraint f-RNNs (S7B Fig, first row and column; see S7C Fig for networks trained on extended and variable delay formats).

In summary, we observed that higher-constraint f-RNNs consistently expressed a “subtractive solution”, consisting of three activity components (see S1 Appendix): (1) a linearly-arranged rank-ordered response to item presentation, (2) an oscillation of frequency ∼0.5 cycles/delay (the subspace of which has component (1)) and (3) a choice axis aligned with the oscillation. These components constituted testable neural activity predictions that we subsequently investigated across RNN variants.

### Geometric signatures of networks performing delay TI

The above findings identify a neural solution to delay TI (Fig 5), which we refer to as the “subtractive” solution, but do not address other possible neural solutions. Given a lack of neural data on delay TI, additional solutions may be biologically relevant and thus important to clarify.

We therefore investigated the remaining RNNs that performed delay TI, doing so by evaluating whether these networks met essential neural activity predictions of the subtractive solution. In the subtractive solution (Fig 5 and S1 Appendix), two essential activity predictions are (1) the linearly-arranged rank-ordered arrangement of activity states (pattern 1), which here refer to as “ordered collinearity,” and (2) the oscillation associated with transitive comparison (pattern 2). We assessed these two patterns in turn.

Surprisingly, we found that pattern 2 was not uniquely expressed in the subtractive solution. A striking indication that this was the case came from analyzing r-RNNs (recurrent-trainable RNNs), an RNN variant that necessarily adopts a different solution, as the subtractive solution depends on the ordered collinear activity made possible by learned feedforward connectivity, which is not available in r-RNNs by definition. Despite this difference, neural activity in r-RNNs appeared similar to that of higher-constraint f-RNNs (S8A Fig, compare to Fig 5A), showing an oscillatory mode consistent with pattern 2 (frequency ∼0.5 cycles/delay; identified either from fixed-point linearization or from linear dynamics inferred from delay period activity, S8B and S8C Fig). Further, as with higher-constraint f-RNNs seen earlier (Fig 5D), analysis of the linearized dynamics revealed that this single oscillation, when combined with the task inputs (item 1—delay—item 2), could accomplish transitive comparison across time (S8D Fig). Interestingly, this was only apparent when the oscillation was applied to full-dimensional activity (S8D Fig, bottom row; further explanation in Methods) and not activity reduced to the 2D subspace of the oscillation (S8D Fig, top row), an indication that the task solution implemented in these networks also depends on higher-dimensional activity components. These results indicate that though r-RNNs cannot adopt the subtractive solution (Fig 5E), the implementation in r-RNNs may nonetheless also rely on a single oscillation. Consistent with this possibility, the oscillatory mode in these networks was consistently aligned with the choice axis (S7B Fig; see also S7C Fig for r-RNNs trained on extended and variable delay formats).

**Fig 6:**
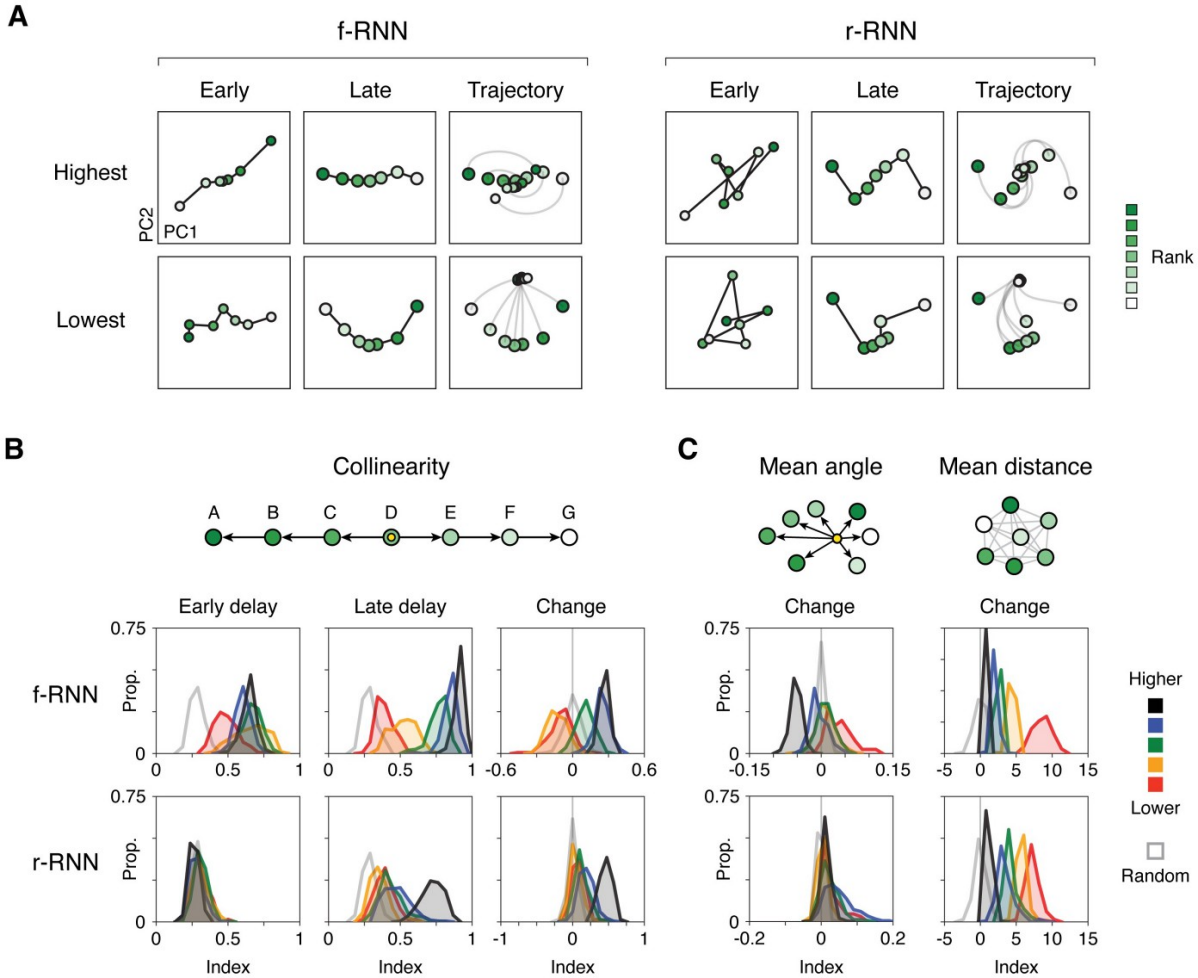
Geometric signatures of networks performing delay TI. **A,** Delay period population activity in four example RNNs. In each example, the seven trial types corresponding to different identities of item 1 are shown (item rank indicated by green shade). PCs were calculated from population activity at the end of the delay. Axes units differ between plots to show the geometric arrangement at each time point. Small vs. large circles indicate early vs. late delay activity, respectively. **B**, Collinearity: schematic (top) and quantification across RNNs performing delay TI (bottom). Top, schematic of geometric arrangement in population activity space. The corresponding index (collinearity index; see Methods) quantifies whether neural activity during the delay matches this geometric arrangement. Angles were measured relative to the cross-condition mean (XCM, yellow circle; shown in example RNNs in Figs 5A and 6A). Bottom, histograms of index values across RNNs. Index values were measured in three respects (columns): early delay, late delay, and change across delay (late—early). Early and late delay were defined here as the first and last timesteps of the delay, respectively; similar results were obtained when using averages from the first and last quarter of the delay. **C**, Mean angle and mean distance: schematics of each measure (top) and histograms of index values across RNNs (bottom). For both mean angle and mean distance, all pairwise angles and pairwise distances, respectively, were calculated in population activity space (between trial types based on item 1, i.e. A, B, C, etc.; green-shaded circles) and averaged. Angles were measured relative to the cross-condition mean (XCM; yellow circle). For both activity geometries, the change during delay (late—early) is plotted. All plots show histograms of instances for each RNN variant (n = 65–200 instances / variant; see Table 2), in addition to randomly generated data (open grey histograms); all quantifications were performed in the top 10 PCs of neural activity during the delay (quantification in top 2 PCs presented in S9B Fig).

By contrast, pattern 1 (ordered collinearity) definitively differed across networks. For example, in r-RNNs, activity at the start of the delay period is not collinear (example r-RNNs in Fig 6A and S9A Fig), as would be expected given the lack of learnable feedforward connectivity in these networks. To assess all networks, we defined quantitative indices for both collinearity and ordered collinearity (ranging from 0 to 1 for collinearity and -1 to +1 for ordered collinearity, schematic in Fig 6B; ordered collinearity presented in S9B Fig. Each index is measured in the full population activity space of individual networks (ambient N-D space, reduced to the top 10 PCs to aid comparison to predominant responses in neural data).

As expected from previous observations (Fig 5), higher-constraint f-RNNs expressed collinearity index values that were relatively high (>0.5) or near 1 (Fig 6B, black and blue histograms in upper row, first and second columns; collinearity, early delay: highest: 0.65 ± 0.05, high: 0.60 ± 0.05; collinearity, late delay: highest: 0.91 ± 0.03, high: 0.85 ± 0.05; mean ± s.d., n = 65–200 for each variant, see Table 2) and that were virtually always higher than that of r-RNNs at the start of the delay (Fig 6B, first column; r-RNN: 0.29 ± 0.05, n = 921 instances) or of randomly generated activity vectors (random vectors: 0.28 ± 0.05, n = 200 draws; equivalent to r-RNNs at the start of the delay).

Interestingly, lowest-constraint f-RNNs expressed significant collinearity at the start of the delay (early delay). In these networks, index values were lower than that higher-constraint f-RNNs, yet consistently higher than that of random activity (Fig 6B, red histograms in upper row, first and second columns; collinearity, early delay: lowest: 0.49 ± 0.09, vs. highest and high, p < 10^−49^, vs. random p < 10^−57^; collinearity, late delay: lowest: 0.39 ± 0.06, vs. highest, p < 10^−76^, vs. random, vs. random p < 10^−42^; rank-sum tests for comparisons; see Table 2 for counts). The expression of significant collinearity at the start of the delay indicates that feedforward connectivity in these networks was modified in training.

Lastly, we found that, as in f-RNNs, different constraint regimes in r-RNNs yielded consistently different activity geometries, thus making it possible to distinguish these RNN variants on the basis of neural activity. In particular, highest- vs. lowest-constraint r-RNNs expressed overtly different degrees of collinearity at the end of the delay period (>0.5 vs. <0.5 in highest vs. lowest, respectively; Fig 6B, black vs. red histograms in bottom row, second column; highest: 0.73 ± 0.08, lowest: 0.39 ± 0.07, p < 10^−32^).

These several results establish that collinearity can be used to distinguish between neural models; the predictions across RNN variants are summarized in Table 2.

Measuring changes in collinearity across the delay clarified additional consistent differences across networks. We noticed that collinearity index values could increase dramatically across the delay period (Fig 6B, early and late delay, left and middle columns; e.g. ∼0.3 to ∼0.8 for r-RNNs trained in the highest regime, Fig 6B, black histograms in bottom row; observable as an “unfolding” of activity trajectories during the delay period in S8A Fig; examples of evolution of angles in S9D Fig). This observation led us to examine how collinearity changes across the delay (Fig 6B, right column). Notably, f-RNNs exhibited systematic differences depending on constraint regime: higher-constraint networks invariably increased collinearity during the delay (Fig 6B, black and blue histograms in top row; collinearity change: highest: 0.27 ± 0.04, high: 0.25 ± 0.06; mean ± s.d., see Table 2 for counts), whereas lower-constraint networks consistently decreased collinearity during the delay (Fig 6B, both lowest and low, red and orange histograms, respectively, in top row; collinearity change: lowest: -0.10 ± 0.10, low: -0.13 ± 0.08; mean ± s.d.).

These findings suggested that different networks implemented different task-relevant dynamics during the delay, in contrast to the previous observation that higher-constraint RNNs generally expressed a similar dynamical pattern (a single oscillation implementing transitive comparison over time, Fig 5 and S8 Fig. We thus conjectured that a single oscillation was potentially one case among a broader set of dynamical patterns enabling transitive comparison over time, and, further, that the essential operation of this wider set of dynamics might be rotation. If so, investigating activity geometry based on changes in angular relationships (i.e. angles between activity states in A vs. B trials, A vs. C, B vs. C, etc.) could help distinguish networks. We therefore evaluated a more general measure of changing angular relationships, which we termed mean angle change (see Methods).

We found that mean angle change distinguished RNNs in a manner correspondent with a previously observed behavior that had two alternative versions across RNNs (Fig 6C, left column; >0 and <0 mean angle changes corresponded to 1st- vs. 2nd-faster end order behavior, respectively, S10A and S10B Fig). In contrast, an analogous measure based on distances in activity space did not (mean distance change; Fig 6B, right column).

Lastly, we assessed whether RNNs performing delay TI showed any particular notable activity geometries differing from ordered collinearity. While activity geometries across networks appeared to vary widely (examples in Fig 6 and S9A Fig, networks often expressed a “V” shape, where activity states corresponding to the items along the transitive schema is “folded” or curved in activity space (f-RNN lowest in Fig 6A; additional examples highlighted in red in S9A Fig). This pattern is similar to that observed in recent studies that consider neural responses in tasks involving transitive judgments between stimuli, though the transitive relation is conveyed explicitly to subjects in these studies [62, 128]. Interestingly, various RNN variants could exhibit this “V” activity pattern (see S9C Fig).

In summary, we found three population activity patterns whose properties differentiated RNN variants (delay-period oscillation, collinearity, and mean angle change), with each pattern constituting testable neural activity predictions (summarized in Table 3).

### A neural basis for order-dependent behavior

To gain further understanding of how various RNNs performed the task, we next sought to clarify the neural basis of the end order behavior, which we earlier found to differ systematically across RNN variants (Fig 4).

We hypothesized that the two versions of the end order behavior, i.e. the 1st-faster vs. 2nd-faster versions (Fig 4), could be due to a simple difference in how networks internally represent (encode) item 1 vs. item 2. We examined encodings along two axes in activity space directly relevant to behavior: (i) the readout axis (linear readout weights of output units), the axis along which activity directly generates a behavioral resonse, and (ii) the choice axis (the direction in activity space from choice 1 to choice 2 trials; see Methods). In preliminary analyses, we found that the choice axis effectively approximates the readout axis across networks (S7D Fig).

**Fig 7:**
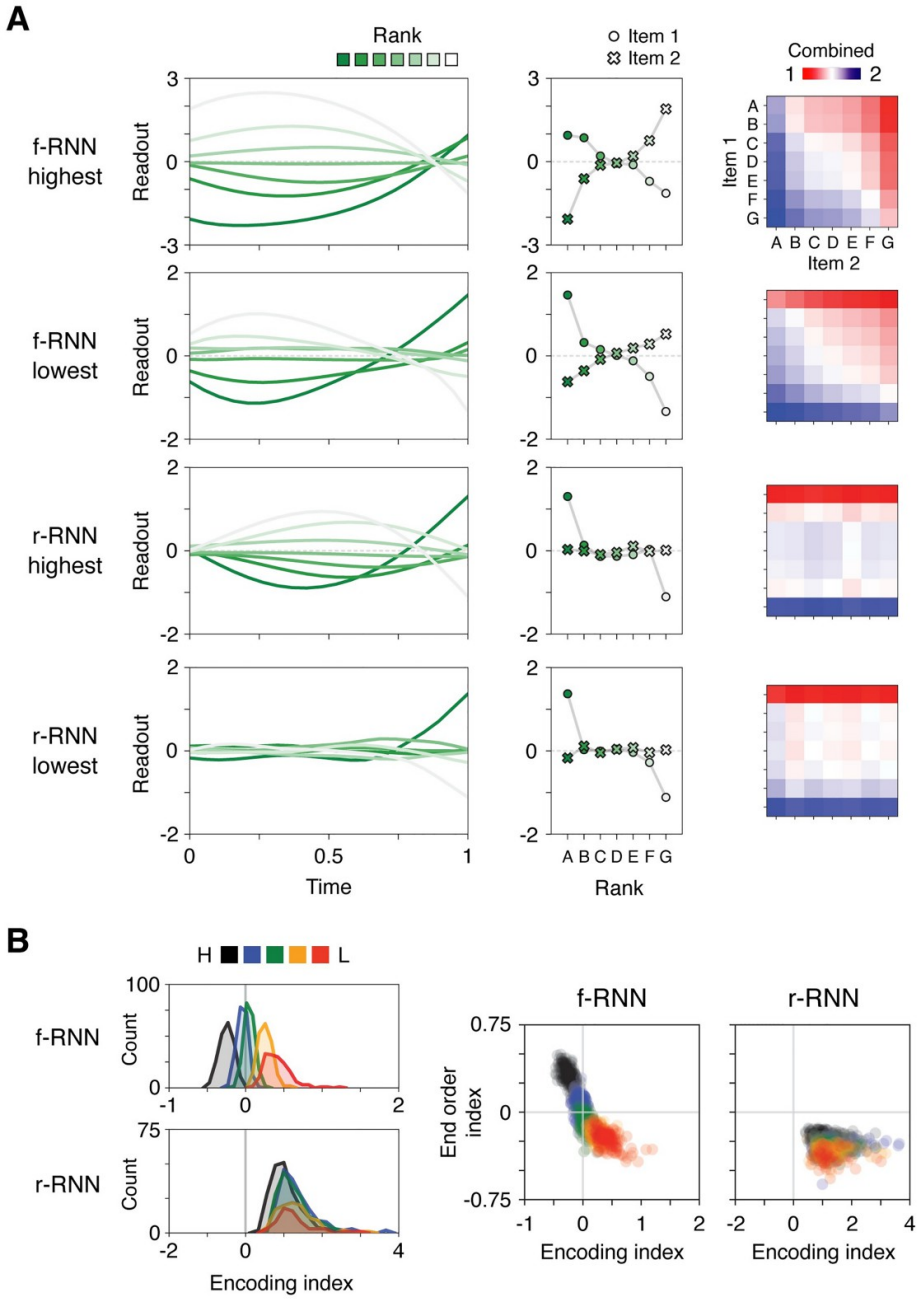
A neural basis for order-dependent behavior. **A,** Neural encodings (axis-projected activity) in four example RNNs (rows). Left column, projection of neural activity along the readout axis (output unit weights). Middle column, item 1 vs. 2 encoding, corresponding to projected neural activity at the end and beginning of the delay, respectively. Right column, sum of item 1 and item 2 encodings. Note that the sum yields large values for trials containing end items, either when the end item is item 1 (rows; examples 2–4) or item 2 (columns; example 1). **B**, Left, histograms of the encoding index across RNNs. The encoding index was defined as the multiplicative gain in the magnitude of end items’ neural encodings (>0: item 1 encoding larger than item 2, ‘1st-dominant’; <0: item 2 encoding larger than item 1, ‘2nd-dominant’). Right, end order index vs. encoding index across RNNs. Note that 1st- vs. 2nd-dominant encodings correspond to 1st- vs. 2nd-faster behavior (end order index <0 vs. >0, respectively). The analogous analyses for the choice axis are presented in S10C Fig.

Examining neural activity projected along either axis revealed two different patterns of encoding across RNNs (Fig 7A), with each pattern varying systematically across RNN variants (Fig 7B). Projections of activity onto the readout axis are shown in example networks in Fig 7A. In a subset of networks, when item 1 is an end item (A or G), it is re-encoded during the delay period such that its encoding magnitude is reduced (Fig 7A, upper row). Conversely, in other networks, recoding in the delay period instead increased the encoding magnitude of end items (Fig 7A, second to fourth rows). These two encoding patterns imply that, for trials containing an end item, either item 1 or item 2 can be dominant in generating behavior. The difference between the former (item 1 dominant, or “1st-dominant”) versus latter (item 2 dominant, or “2nd-dominant”) was illustrated by directly summing the encoding values of item 1 and 2 (Fig 7A, right panels). These encoding patterns can be quantified in any neural system performing delay TI by evaluating the choice-axis projection of neural activity taken from the end vs. beginning of the delay period, which provide estimates of the encoding value of item 1 (due to recurrent input) and item 2 (due to feedforward input), respectively. We accordingly quantified the encoding values of item 1 and item 2, which we then compared as a ratio (encoding ratio, item 2: item 1; using the readout axis in Fig 7B, and the choice axis in S10C Fig, each approach yielded qualitatively similar results).

We found that the encoding ratio predicted end-order behavior across all RNNs (Fig 7B, right panels). Thus these two qualitatively different encoding strategies (1st vs. 2nd-dominant) suggest an empirically testable neural basis of the end order behavior, and moreover constitute testable neural predictions distinguishing RNN variants (summarized in Table 3).

### Delay TI in human subjects

The above investigation indicates that there are different neural solutions to TI when WM is required, a finding that stemmed from imposing an intervening delay between item presentations (Fig 1C and S1 Fig). Yet despite the extensive literature on TI [52, 88], prior studies generally do not impose such a delay (though see [108] for a probabilistic version of TI): rather, items are presented either simultaneously (no delay, Fig 1C; e.g. left and right images on a screen) or as encountered at the discretion of the subject (e.g. odors in separate containers, conspecifics in separate chambers [57, 96, 97]). There is thus a lack of experimental data for testing predictions pertaining to the WM delay (e.g. the end order pattern, Fig 4); experimental data is also needed to evaluate whether standard TI behaviors are expressed when WM is explicitly required.

**Fig 8:**
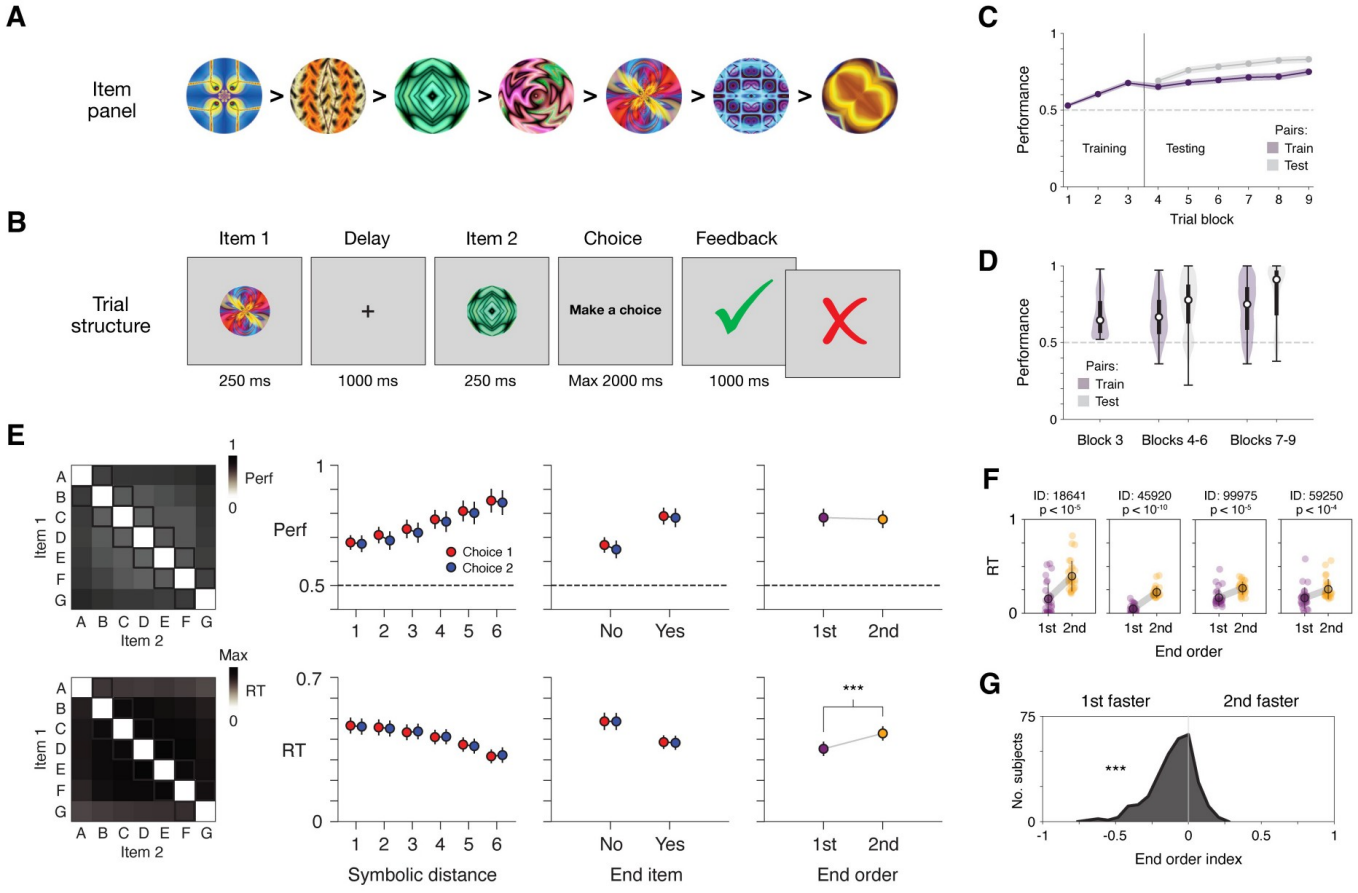
Delay TI in human subjects. **A**, Item panel. Each item is a fractal image. Shown is an example transitive ordering of images; for each subject, the order was randomly generated. **B**, Schematic of trial structure. **C**, Performance over the course of task. Training phase: blocks 1–3. Testing phase: blocks 4–9. Shaded region indicates ± 2 s.e.m. **D**, Variability in performance across subjects. Plotted are distributions of performance at three task phases: late training phase (block 3), early testing phase (blocks 4–6), and late testing phase (blocks 7–9). Each distribution is shown as a violin plot (Gaussian kernel, s.d. = 0.3) and box plot (median, IQR, and range). **E**, Behavioral patterns across subjects. Data from early testing (blocks 4–6); behaviors and plotting conventions follow those in Fig 3. Results are presented in two rows (top row: performance (proportion correct); bottom row: RT (proportion of maximum value (Max; 522 ms) in trial type matrix (leftmost plot); in sec otherwise)). Column 1: averages across subjects by trial type. Columns 2–4: mean ± 3 s.e.m. across subjects. Trial types follow those in Fig 3A (column 2: symbolic distance; column 3: end item; column 4: “end order”, ***p < 10^−23^, signed-rank test), in addition to distinguishing between choice 1 vs. choice 2 trial types (red vs. blue, respectively). **F**, End order behavior in four example subjects. Each plot shows RTs (in sec) from an individual subject (colored circles: individual trials; dark circle and error bars: mean ± s.d.), separately plotting trials in which the end item (A or G) occurred either 1st or 2nd (p-values from rank-sum tests comparing RTs of 1st vs. 2nd trials). Of 292 total subjects, 189 (65%) vs. 103 (35%) showed the 1st vs. 2nd faster pattern; of these subjects, 48% (1st-faster) vs. 6% (2nd-faster) showed significance at the p < 0.01 level (rank-sum tests). **G**, Histogram of end-order behavior across individual subjects. The behavior was quantified as the difference of average RTs divided by their sum (end order index; average RTs calculated for trials where end items (A and G) occurred 1st vs. 2nd; ***p < 10^−24^, signed-rank test). All data from n = 292 subjects, with panels e-g presenting data from early testing (blocks 4–6).

We therefore tested delay TI in humans in a large-scale experimental study (392 subjects) using Amazon Mechanical Turk, an online platform enabling testing human subjects on cognitive tasks. As with the neural models, a panel of 7 arbitrary items (fractal images, corresponding to items A to G; Fig 8A) was used; further, within each trial, an intervening delay period (1 sec) was imposed between item presentations (Fig 8B). In each trial, subjects chose either item 1 or 2 via key press (“D” or “K” key, corresponding either to item 1 or 2), and were informed of trial outcome (correct/incorrect) immediately upon choosing. To promote task engagement, subjects were incentivized to select correctly so they could earn performance-based bonus money. Training was performed for a fixed number of trials (training phase; 144 trials consisting solely of training trial types, i.e. A vs. B, B vs. C, etc., divided into 3 blocks of trials), after which testing was conducted (testing phase; 252 trials consisting of both training and testing trial types, divided into 6 blocks of trials). Upon completion of testing, subjects were asked to describe how they performed the task (example responses in S2 Table; word count tabulation in S12 Fig).

To assess the task paradigm, we first evaluated whether subjects successfully performed inference. We analyzed two groups of subjects: (i) those showing above chance (>50%) performance on training trials in the final training phase block (292 of 392 (74%) subjects), the minimal degree of task ability prerequisite to testing inference, and (ii) those proficient in training trials (>80% performance in final training phase block; 65 of 392 (17%) subjects), similar to previous studies that initially establish TI task paradigms [82, 83]. On the first presentation of ‘critical’ trial types (testing trials not containing end items; e.g. B vs. D), either group of subjects performed well above chance: for (i), 60% ± 1.2% (s.e.m.) (vs. 50%, p < 10^−14^, signed-rank); for (ii), 75% ± 2.4% (vs. 50%, p < 10^−9^, signed-rank); subsequent analyses were carried out in the more inclusive group (i), as previous work on TI indicates that behavioral patterns can manifest even when subjects show relatively low performance on training trials (e.g. [129]). Across the testing phase, these subjects performed consistently above chance (65–85% mean performance, Fig 8C), while also showing considerable variability in performance (Fig 8D), as is generally observed in TI studies (e.g. [130]).

Importantly, subjects doing delay TI showed the classical behavioral patterns seen in previous TI studies, a result that was apparent when trial types were considered (Fig 8E, and S5 Fig). In particular, both symbolic distance and end-item effects were expressed, each in both performance and RTs (symbolic distance: Fig 8E, 2nd column; end-item: Fig 8E, 3rd column).

The above findings—indicating both successful inference and classical TI behaviors—introduce delay TI as a viable experimental paradigm, and, further, suggest that subjects’ performance of delay TI may share an underlying basis with that of traditional TI task paradigms.

We lastly evaluated whether the behavioral data were better aligned with particular neural models. To do so, we focused on the end order effect, the novel behavioral prediction that had two different versions depending on the RNN variant (Fig 4)—we reasoned that analyzing this behavioral pattern would thus provide particularly clear grounds by which to adjudicate between the neural models. We found that subjects widely expressed the end order effect, initially observable in plots of RTs by trial types (Fig 8E, trial-type matrix of RTs showing lower values in first and last rows; S4B and S5D–S5F Figs, manifesting as an “X” pattern in average RTs), both of which specifically indicated that subjects responded faster when the end item (A or G) was the 1st item (1st-faster version); plots of RTs averaged across trial types (Fig 8E, last column) and RTs in individual subjects (Fig 8F, additional quantification in caption) confirmed the effect. As with separately trained instances of neural models (Fig 4), we measured the end order effect across individual subjects using a quantitative index, finding that 1st-faster behavior occurred on a distribution that was wide yet skewed to values <0 (vs. 0, p < 10^−24^, signed-rank) (Fig 8G). Strikingly, this distribution was overtly inconsistent with that of higher-constraint f-RNNs, which consistently show values >0 (Fig 4), with quantitative comparison of index values underscoring the mismatch (S11 Fig). This result suggests that higher-constraint f-RNNs, though characteristically expressing an intuitive solution (“subtractive” solution; Fig 5E and S1 Appendix), may not accurately reflect the underlying system responsible for performing the task.

## Discussion

In this study, we generated, analyzed, and experimentally tested a collection of neural models of transitive inference (TI), a classical cognitive task that distills relational inference into a simple yet essential form. Motivated by the naturalistic and potentially intrinsic interrelationship between relational inference and working memory (WM) [53, 54, 103, 104], our study introduces a new task paradigm—“delay TI”—that imposes an explicit WM delay between presented items. We found that trained recurrent neural networks (RNNs) not only performed delay TI, i.e. generalized to all novel combinations of inputs (Fig 2), but also expressed behavioral patterns long documented in living subjects performing TI (Fig 3). Investigating delay TI also disclosed a previously undescribed order-dependent behavior, the “end order” effect, which was expressed in RNNs in either of two distinct versions (1st- vs. 2nd-faster versions, Fig 4).

We subsequently identified a neural solution to delay TI characterized by simple collective dynamics and geometry (population-level “subtraction,” Fig 5 and S1 Appendix). This solution, which was expressed in a subset of RNNs optimized for efficiency and having modifiable feedforward connectivity (higher-constraint f-RNNs), led us to identify a set of testable activity predictions unique to these models, among other models that successfully performed the task (summary of main predictions in Table 3).

Lastly, in a large-scale experimental study of delay TI, we found that human subjects successfully performed the task and also showed classic behavioral patterns seen in traditional TI tasks (Fig 8). Further, subjects expressed the end order effect, doing so in mainly one of two alternative versions (1st- rather than 2nd-faster), thus providing grounds for ruling out neural models uniformly expressing the alternative version (2nd-faster).

Prior work on relational inference in the brain has often focused on task paradigms that rely on stimuli that are challenging to isolate (e.g. spatial tasks [21, 24, 47, 131, 132]), test multiple relations at once (e.g. tasks with linguistic responses and/or episodic elaboration, e.g. [133–136]), or do not require behavioral report of inference. Possibly as a result, there are relatively few hypotheses and available models that clarify or explain how neural systems accomplish relational inference (generalize in accordance with a relation) at the explanatory levels of population-level neural activity and behavior. In the present study, we developed a task paradigm (delay TI) and a neural approach (task-trained neural networks) suited to meet these challenges. It is also worth emphasizing that the TI paradigm presently studied is implicit, i.e. does not provide semantic or isolated perceptual cues regarding the underlying transitive relation, thereby minimizing the role of linguistic ability and affording a bridge to the extensive literature on TI in animals [52, 88].

We initially found that RNNs trained via standard optimization procedures commonly performed TI perfectly (transitive generalization; Fig 2, Table 2 and S1 Table). By itself, this finding is notable since it is not generally known whether largely unstructured learning models, which trained RNNs and other NNs exemplify, implement the inductive bias required for transitive generalization (examples of feedforward models in S2 Fig and [56, 62, 63]). Interestingly, this finding raises the question of what exact components of learning systems implement such ‘relational’ inductive biases [5], for which transitivity is an archetype. Indeed, in TI, existing reinforcement learning (RL) models, which represent the most behaviorally relevant models to date [55], have sidestepped this question. On the one hand, RL models that cannot acquire internal representations fail to perform TI or show behavior that is qualitatively non-naturalistic, thus disqualifying these models (e.g. Q-learning and value transfer models [55]). On the other hand, other RL models used to study TI have been pre-configured to have transitive or otherwise ordered internal representations (e.g. a score or set of ordered lists [55]). By contrast, our observation that TI was often expressed in trained RNNs, learning systems that are not pre-configured as such, invokes the possibility that relational inference can emerge in a wide range of learning systems, by virtue of more general principles (see [63] for recent work focusing on TI in feedforward models). We highlight this matter as an important direction for future work: in our investigation, we instead focused on how models generalized after having acquired the ability to respond correctly on training trials (AB, BC, CD, etc).

Crucial to our approach was to identify, where possible, multiple solutions to TI. To do so, we investigated whether and how RNNs performed delay TI when two neurobiologically relevant factors varied—learnable connectivity (i.e. fully-trainable RNNs (f-RNNs) vs. recurrent-trainable (r-RNNs)) and constraint regime (regularization and initial connectivity strength; higher vs. lower constraint regimes) (Fig 1D, Table 1). We found that each of these RNN variants could perform TI, and moreover identified a set of behavioral and neural predictions that distinguish between four representative variants (Table 3). It was further notable that different RNN variants expressed different versions of the end order behavior (Fig 4, Table 3, second column).

At the same time, the neural activity expressed in the various networks performing TI suggested a common dynamical principle: namely, rotation. This was initially suggested by the “subtractive” solution (Fig 5), in which a single oscillation rotates activity states in activity space during the delay. Beyond this solution, we observed that a single oscillation was also sufficient dynamics to perform TI even in networks that could not express an orderly arrangement of activity states from feedforward input (due to lack of modifiable feedforward connectivity; higher-constraint r-RNNs, compare Fig 5 with S8 Fig); this indicates that a single rotational transformation can be used to perform delay TI for additional activity geometries elicited by feedforward input. More broadly, a single oscillation could be a simple case of a wider set of dynamical patterns enabling transitive generalization, with rotation as the essential operation. The idea of generalized rotational transformations also led us to identify an angle-based activity pattern that distinguished all RNN variants in accordance with task behavior (mean angle change, Fig 6C; behavior prediction in S10B Fig).

Geometric analyses of activity (Fig 6), in addition to the ability of RNNs lacking modifiable feedforward input (r-RNNs) to perform the task, indicated that there exist a variety of activity geometries capable of supporting transitive generalization. One intriguing example is the “V”-shaped arrangement of activity states seen in lower-constraint RNNs (the “V” manifesting at the end of the delay and differing from the more strictly collinear geometry seen in higher-constraint f-RNNs; examples in Fig 6A and S9A Fig; see also S9C Fig), similar to that reported in recent studies testing human subjects on a transitive hierarchy of items [62, 128]. These results highlight an unexpected connection between lower-constraint RNNs and experimentally observed activity geometry in related tasks; these results raise the possibility that the observed “V” activity geometry was generated by learned recurrent dynamics (rather than particular input encodings or other mechanisms [62]).

In trials containing end items (A and G), RNNs showed systematically different representational strategies (Fig 7). In lower-constraint RNNs and r-RNNs, the encoding of end items was amplified during the delay (‘1st-dominant’ encoding), whereas higher-constraint f-RNNs showed reduction during the delay (‘2nd-dominant’). The 1st-dominant strategy appears advantageous as it facilitates faster responses when item 1 is an end item—that is, when item 1 already sufficiently indicates the correct response irrespective of item 2. However, the 2nd-dominant strategy is plausible, or even advantageous, under conditions of uncertainty regarding item 1, e.g. due to forgetting across the delay or incomplete understanding of the underlying relationship across items. For example, in a scenario where all trial types are equally likely, a subject with an optimal response strategy but no memory of item 1 can still respond predominantly correctly (>75% of trials on 7-item TI); such a response strategy effectively treats item 1 (irrespective of whether A, B, etc.) as the middle item, as is consistent with the lower magnitude of item 1 encoding (of the end items) in the 2nd-dominant strategy. In our behavioral study, it was not clear how often and when subjects were aware of the significance of the end items (see example debriefing responses in S2 Table), though prior work indicates that TI can be performed independently of awareness [137]. In addition, it is relevant that both primacy and recency biases—analogous to the 1st- vs. 2nd-dominant strategies—have been extensively (and variably) documented in memory tasks [138–140], though whether and how such biases are expressed in inferential tasks remains generally unknown. These various observations suggest that either the 1st- or 2nd-dominant strategy are plausible in delay TI, and should both be considered in further investigation of the task (such as in additional species or task variants).

Across the RNNs studied, it is worth highlighting that 1st vs. 2nd-dominant encoding of end items (Fig 7) correspond to the relative magnitudes of recurrent vs. feedforward input; in this regard, higher-constraint f-RNNs were exceptional among RNN variants in expressing stronger feedforward-based encoding. Thus, in these neural systems, constraint regime determined the relative contributions of feedforward vs. recurrent input to a behaviorally relevant representation. This implementational difference implies different underlying neural substrates, and is relevant to the basis of not only TI, but any number of other tasks for which different implementations in the brain are plausible [80, 127].

More directly, our experimental study of delay TI in human subjects provided empirical grounds for discriminating between neural models. Besides finding that subjects showed well-established TI behaviors (Fig 8E), we found that subjects showed the 1st-faster version of the end order behavior, both individually and in aggregate (Fig 8E and 8F). This finding excludes highest-constraint f-RNNs, as these models consistently show the 2nd-faster behavior, and instead suggests that neural models expressing the 1st-faster version may be more accurate. RNNs expressing the 1st-faster version (Table 3) have three unorthodox properties worth highlighting: (1) non-learnable feedforward connectivity (r-RNNs), (2) relatively larger scale of initial connectivity weights (intermediate- and lower-constraint), and (3) weaker or no regularization (intermediate- and lower-constraint). (It is also worth noting that, among RNN variants, intermediate-regime f-RNNs provided the best quantitative match to human data (S11 Fig).) Though we do not here interpret RNN training as a model of task learning in the brain, these three properties may nonetheless be understood as high-level constraints on the neural system in the brain responsible for acquiring and expressing delay TI, and potentially relational abilities more generally.

These properties invoke several further considerations regarding underlying neurobiology and behavior. Property (1) implies that the neural system in the brain responsible for performance of the task may rely upon learned recurrent dynamics rather than on learned feedforward input—two fundamentally different neural implementations of the underlying relational ability. Indeed much prior work on neural substrates of relational abilities (whether construed as schemas, relational memory, semantic knowledge, etc.) has focused on feedforward operations, despite the fact that the relevant neural systems are invariably extremely recurrent. In addition, the empirically suggested plausibility of properties (2) and (3) is unusual in that these properties run counter to prior work indicating that task-trained RNNs best matching neural responses in the brain have the opposite properties, i.e. relatively smaller scale of initial connectivity weights and strong regularization [48, 67, 72, 141].

Further experimental data is required to distinguish between neural models. Importantly, four representative RNN variants in our study were distinguished by neural activity predictions (at the population level; summarized in Table 3); it is thus in principle possible to discriminate between these models with experimentally collected neural data. With respect to brain regions, any number of brain structures receiving appropriate sensory input and capable of supporting WM and the learning of training trials are relevant; TI has moreover been observed in a wide variety of animal species [52, 88]. In the mammalian brain, the leading candidates are brain regions linked to relational inference and/or WM, including prefrontal cortex (PFC) and hippocampus [142–148], both known to be required for TI performance [90, 149]. Discriminating between neural models of delay TI has the potential to provide insight beyond how these brain regions enable TI, but also how these brain regions contribute to performance of other cognitive tasks. Such insights could arise both from the structure of specific solutions and from identifying high-level constraints (e.g. learnable connectivity).

The findings in the present study may also relate to the neural basis of other cognitive functions. Of direct interest are neural activity patterns relevant to abstraction—whether at the level of single cells (e.g. place and grid cell firing [14, 85] and other firing having abstract correlates [150, 151]) or neural populations (e.g. activity geometries [45, 72, 152–154], dimensionalities [73, 155–157], and re-activation patterns [158–161] suitable for generalization). In our approach to TI, we deliberately chose not to seek to fit or capture these neural activity patterns, instead stipulating relatively unconstrained neural models. Indeed there may exist important relationships between such activity patterns and those expressed in the neural models here presented. It is also worth emphasizing that our findings do not directly address learning processes, for which prior studies have proposed various models and mechanisms [55, 108, 162–166] (including for explicit variants of TI, where human subjects are informed of the transitive hierarchy [62, 167, 168]). Further, our analyses and neural activity predictions focus on delay period activity, leaving open the question of whether and how neural activity following presentation of both items may contribute to transitive generalization. More broadly, it is worth pointing out that there exist any number of other types of relational inferences (e.g. spatial navigation), tasks, and scenarios that incorporate considerably higher, and potentially important, complexity. In conjunction, in the brain, the relevant regions linked to TI and relational inference also support or pertain to cognitive capacities such as structure learning, episodic and semantic memory, and imagination. This convergence of diverse cognitive functions indicates that, toward understanding their biological basis, there is a major need to synthesize approaches.

## Materials and methods

### Ethics statement

#### Animal data

For comparison to models (S5 Fig), we present data from a previous behavioral study of traditional TI in Rhesus macaques (originally presented in [51, 52]). The research was approved by the Institutional Animal Care and Use Committee of Columbia University (AAAI1488).

#### Human study

The human study was approved by the Institutional Review Board (IRB) at Columbia University through Columbia IRB Protocol #AAAI1488. All participants provided written informed consent for their participation in the experiment.

### Task

Transitive inference (TI) is a classic cognitive task that requires subjects to infer an abstract relation—here, transitivity (Fig 1A)—between items not previously observed together, i.e. using A > B and B > C to infer A > C (Fig 1B). TI defines test cases expressly as novel recombinations of training inputs, thus primarily testing relational rather than statistical inference. We focused on a 7-item version of TI, in which there are 12 training trials and 30 test trials (training: A vs. B, B vs. C, etc.; test: A vs. C, B vs. D, etc.; see Fig 1B for diagram of trial types and correct responses), though in pilot work we found that our approach could be generalized to fewer or more items with qualitatively similar results.

Given the interrelationship between relational inference (exemplified by TI) and working memory (WM) [53, 54], we investigated TI in a task format that explicitly imposes a delay that necessitates WM (S1A Fig, delay format). Further, for comparison to previous modeling work [55, 56] and for potential insight, we also studied the traditional task format, for which the presentation of task items (A, B, C, etc.) is simultaneous (traditional TI, diagram in Fig 1C). It is worth noting that in some prior TI studies, a delay between stimuli is implicit in the free exploration afforded to subjects (e.g. [57]).

Besides WM, an important difference between the delay vs. traditional format is that the traditional format requires twice as many input parameters as the delay version (e.g. twice as many input connections in a neural system). This difference may make the delay version not only more difficult to perform, but also more neurobiologically accurate with respect to neural systems underlying abstract cognition: these systems look to receive extremely diverse inputs, implying that the extent of input connectivity is relatively constrained [58].

### Input stimuli (items)

A panel of input stimuli corresponding to items A, B, C, etc. was constructed for each model instance. In TI in living subjects, an item is an arbitrary sensory object (e.g. image, odor) with no features that are significant *a priori*. To capture this property, items were represented as randomly generated input vectors **u** (**u**^*A*^, **u**^*B*^, etc.), modeling sensory-driven activity in upstream neurons. For simplicity, each **u** was drawn from a multivariate standard normal distribution of dimension *N*^*in*^.

The identity of items 1 and 2 varied by trial type (e.g. AB, BA, AC, BC, etc.; see Fig 1B for all trial types). *N*^*in*^ was chosen to be 100, matching the size of the hidden layer of the neural models (MLP and RNN), which was set to *N* = 100 (see below). This ensured that input corresponding to each item presentation elicited patterns of activation that would be uncorrelated in the activity space of the neural models (at least prior to training) thereby simulating arbitrary sensory stimuli. Note that TI (and relational inference more generally) is not defined in terms of particular stimulus features, and indeed is most rigorously tested in the absence of any stimulus features indicating items’ rank in the transitive hierarchy [59].

### Model architectures

Three model architectures were studied: a recurrent neural network (RNN), logistic regression (LR), and a multi-layer perceptron (MLP). Each was implemented in Python using the NumPy and PyTorch [60] packages, in addition to custom code for RNNs and all subsequent analyses. A repository of trained models used in this study is available on DataDryad [61].

#### Recurrent neural network (RNN)

To investigate population-level neural dynamics, we studied the standard continuous-time RNN:

$$\begin{array}{c} \tau \dot{x_{i}}\left(t\right)=-x_{i}\left(t\right)+\sum_{k=1}^{N}J_{ik}r_{k}\left(t\right)+\sum_{k=1}^{N^{in}}B_{ik}u_{k}\left(t\right)+b_{i}+\eta_{i}\left(t\right) \end{array}$$

where *x*_*i*_ are activity of the recurrent units, *r*_*i*_ are the corresponding rates, *N* is the number of recurrent units (100 for all networks), *N*^*in*^ is the number of input units, and *τ* is the unit time constant. As we sought to develop models applicable to various neural systems that perform TI (e.g. across animal species), we did not set a unit for *τ*; rather, times were expressed in units of *τ*. The rates *r*_*i*_ derive from the activations *x*_*i*_ via a tanh nonlinearity, *r*_*i*_ = tanh(*x*_*i*_).

The tanh non-linearity was chosen because we found it to be the most effective for generating and analyzing network dynamics; further, in pilot work we found that other non-linearities (e.g. rectified tanh) yielded RNNs that exhibited unrealistic behavioral patterns when simulated (see below for description of behavioral simulations; rectified-tanh RNNs exhibited an unrealistic response bias favoring choice 1 over 2, or *vice versa*). We note that RNNs in the present study are intended to model neural activity (and generate testable predictions) at the neural population level.

The network units interact via the recurrent synaptic weight matrix **J**. The input to the system is **u**, the activity of the set of *N*^*in*^ input units that influence the network through input weights **B**. The output of the system is **z**, the activity of a set of *N*^*out*^ output units, each defined to be a linear readout of activity in the recurrent units:

$$\begin{array}{c} z_{i}\left(t\right)=\sum_{k=1}^{N}W_{ik}r_{k}\left(t\right)+b_{i} \end{array}$$

Each output unit *z*_*i*_ is a weighted sum of network rates with weights, **W**_**i**_, with a constant bias, *b*_*i*_. In all models, three output units were implemented (*N*^*out*^ = 3), corresponding to three alternative behavioral actions (see below, Model output). All analyses of neural activity were performed on *x*_*i*_; analyses of *r*_*i*_ yielded similar predictions.

Network simulations were performed using Euler’s method with discrete time step Δ*t* = *τ*/10. Intrinsic single-unit noise *η*_*i*_(*t*) was generated at each time step by drawing values from a Gaussian random variable with zero mean and s.d. of 0.2.

Prior to training, the parameters of the model were initialized as follows. The entries of **J** were initialized as draws from a normal distribution with zero mean and variance $g_{0}^{2}/N$. The entries of **B** were initialized as draws from a normal distribution with zero mean and variance $h_{0}^{2}/N^{in}$. The elements of **W**, and all bias terms, were initialized to 0. Both *h*_0_ and *g*_0_ were hyperparameters that were systematically varied across RNNs (see Table 1 and RNN variants below).

#### Logistic regression (LR)

The LR model (schematic in S2A Fig) was studied to clarify the possible role of feedforward connectivity in performing TI. Each LR consisted of two linear readouts (corresponding to choice 1 and 2) each of which had coefficients for every input dimension *N*^*in*^ for each of the two items presented. In each simulation of the model, Gaussian noise (zero mean, s.d. of 0.2) was added to each of the readouts.

#### Multi-layer perceptron (MLP)

In addition to the LR model, single-layer MLPs (schematic in S2A Fig; N = 100 hidden units, fully connected) were studied to clarify the possible role of feedforward connectivity in TI. Entries of the input weight matrix were initialized as draws from a normal distribution with 0 mean and variance $h_{0}^{2}/N^{in}$, with *h*_0_ = 1; entries of the output weight matrix were initialized to 0; all biases were initialized to 0. In each simulation of the model, Gaussian noise (zero mean, s.d. of 0.2) was added to each of the hidden units. Relevant results in MLPs are also reported in several previous studies (see [56] for MLPs with three hidden units solving five-item TI, and [62] for MLPs constrained to have symmetric input weights; see also [63] for a range of feedforward models, including MLPs).

### Model input

#### RNN

The input to the RNN in trial *m*, **u**(*t*, *m*), consisted of the presentation of items 1 and 2 with an intervening delay, dividing three periods in each trial: rest, delay, and choice (S1A Fig). Item presentations were modeled as instantaneous pulses (one timestep) as TI (and relational inference more generally) is not dependent on sensory input of a particular duration.

RNNs were trained on one of three input formats in which the duration of the delay period differed (delay variants): (i) basic, (ii) extended, and (iii) variable. In (i), the rest, delay, and choice periods lasted 0.5*τ*, 2*τ*, and 2*τ*, respectively. A delay duration of 2*τ* was sufficiently long to yield different neural implementations of TI in trained networks, and is the minimal delay duration relevant to working memory [64, 65]. In (ii), the trial periods lasted 0.5*τ*, 6*τ*, and 6*τ*, respectively. In (iii), the rest period and total trial duration were the same as (ii), but the delay duration was varied randomly from 2*τ* to 6*τ* (uniformly across individual trials by shifting the time of item 2 presentation); subsequent testing and simulations of RNNs trained on (iii) were performed using the maximum delay duration (6*τ*). All delay variants yielded RNNs that performed TI (S1 Table) and made qualitatively similar behavioral predictions (S4 Fig); unless indicated otherwise, results from RNNs trained on (i) are presented throughout the study.

#### MLP and LR

The input to the feedforward models (LR and MLP) in trial *m*, **u**(*m*), consisted of the joint (simultaneous) presentation of items 1 and 2, requiring twice the input dimensionality of RNNs (i.e. given a fixed dimensionality for individual input stimuli, i.e. items); thus the feedforward models had *N*^*in*^ = 200 rather than *N*^*in*^ = 100 as in the RNNs (diagramed in S2A Fig).

### Model output

#### RNN

The output from the RNN was composed of three output units *z*_1_, *z*_2_, *z*_3_ corresponding to three behaviors: choice 1, choice 2, and rest, respectively. In training, the target output $\hat{z}\left(t,m\right)$ was defined for every time point *t* and for each trial type *m* such that the correct output unit was activated above resting levels during the choice period (target values: resting: 0, activated: 5; diagramed in S1B Fig).

In example outputs (Fig 2, top row, and S3A Fig), the choice value plotted was the difference in the readout values for *z*_1_ and *z*_2_ averaged over the last half of the choice period and normalized to the magnitude of the largest such difference value across all trial types.

#### RNN behavior

The behavior (i.e. the choice response and response time (RT)) of an RNN in a trial was defined using an established criterion [66]. The *z*_1_ (choice 1) and *z*_2_ (choice 2) output units were passed through a simple monotonic saturating function ranging in value from 0 to 1:

$$\begin{array}{c} {\tilde{z}}_{i}=\frac{1}{2}\text{tanh}\left(z_{i}-\frac{{\hat{z}}_{i}}{2}\right)+\frac{1}{2} \end{array}$$

where ${\hat{z}}_{i}$ is the target value of the output unit.

The **response** (choice in trial) was defined by the identity of the output unit (choice 1 vs. choice 2, see above) that first reached a fixed threshold value of 85% in the choice period. Under certain conditions (i.e. when additional noise was added to RNNs (see further below), or cases when the RNN was presented with same-item stimuli, e.g. AA, BB, CC, etc., Fig 2; these trial types were not evaluated), the threshold was not reached for output unit. These trials are shown in plots as ‘no response’ trials (Fig 2).

The **RT** was defined as the time of the response, measured as the time elapsed from *t*_2_ (the time of presentation of item 2), normalized to the maximum duration of the choice period (0 to 1). In a subset of plots, the RT was normalized to the maximum RT observed across trial types (e.g. trial-type matrices of RTs in the first column of Fig 3), and described accordingly.

#### MLP and LR

For both feedforward models, the output was composed of two output units *z*_1_, *z*_2_, corresponding to choice 1 and choice 2, respectively. In training, the target output $\hat{z}\left(m\right)$ was defined for each trial type *m* such that the correct output unit for each trial type (choice 1 vs. 2, Fig 1B) was activated (value for active: 1, value for not active: 0). The response for a given trial was defined by the identity of the output unit which had the higher activity value. In example outputs (S2B Fig), the choice value plotted was the difference in the readout values for *z*_1_ and *z*_2_ normalized to the magnitude of the largest such difference value across all trial types.

### Model training

Models were optimized (trained) solely on training trials and not test (inference) trials. The ability of trained models to respond correctly to inference trials thereby mirrors that of living subjects that have only experienced or learned from training trials. In this way, analysis of models that respond correctly on test trials (i.e. perform inference) can be studied to identify putative neural implementations. Parameter updates were performed for batches of training trials, where each batch consisted of 128 trials randomly sampled from the training trial types defined by the task (diagramed in Fig 1B).

#### RNN

RNNs were trained to minimize *E*_*task*_, the average squared difference between **z**(*t*, *m*), the readout of the network on trial m, and $\hat{z}\left(t,m\right),$ the target output for that trial:

$$\begin{array}{c} E_{task}=\frac{1}{MTN^{out}}\sum_{m,t,j=1}^{M,T,N^{out}}{\left(z_{j}\left(t,m\right)-{\hat{z}}_{j}\left(t,m\right)\right)}^{2} \end{array}$$

where m corresponds to different training trials, T corresponds to the length of the trial (in time steps), and *N*^*out*^ is the number of readout units. *E*_*task*_ stipulates that the optimization procedure generate networks that respond correctly in training trials.

The overall error function *E* was comprised of *E*_*task*_ and two additional terms that implement regularization, which has been found to promote neurobiologically accurate solutions in trained RNNs [48, 67, 68]. The two terms were *R*_*L*2_, a standard L2 regularization on input and output synaptic weights, and *R*_*FR*_, a regularization on the network rates.

The overall error function was

$$\begin{array}{c} E=E_{task}+\alpha R_{L2}+\beta R_{FR} \end{array}$$

where the *α* and *β* hyperparameters set the strength of each type of regularization.

The first regularization term is a standard L2 penalty on input and output synaptic weights:

$$\begin{array}{c} R_{L2}=\sum_{i,j=1}^{N,N^{in}}B_{ij}^{2}+\sum_{i,j=1}^{N^{out},N}W_{ij}^{2} \end{array}$$

The second regularization term is a metabolic penalty on rates in the network:

$$\begin{array}{c} R_{FR}=\frac{1}{MTN}\sum_{m,t,i=1}^{M,T,N}r_{i}{\left(c,t\right)}^{2}\Delta t \end{array}$$

Both terms have been found to promote neurobiologically realistic responses in trained RNNs (e.g. [48, 67, 68]).

The objective of training was to minimize E by modifying the network parameters **J**, **B**, **W**, **x**(t = 0), and constant bias terms.

Training was implemented with the Adam optimizer [69], with updates to the network parameters calculated using backpropagation through time [70, 71]. Parameter updates were performed for training sets (batches) of 128 trials, where the trials in each batch were randomly sampled from the task-defined training trials (diagramed in Fig 1B). Training was stopped when the RNN responded correctly on all trial types (training and test; see above for response criteria) in the absence of noise (*η*_*i*_ set to 0), or when *E*_*task*_ fell below 0.1; for RNNs trained on the variable delay input format, correct responses were further required when the time of item 2 was advanced earlier in time by 67% of the longest delay duration. Up to 30,000 training epochs were run.

#### LR and MLP

Each feedforward model was trained using the Adam optimizer with cross-entropy loss. For the LR models, training was performed to convergence (i.e. until loss did not improve for 1000 training epochs); for MLPs, training was performed until the network responded correctly on all trial types (training and test; see above for response criteria) in the absence of noise (*η*_*i*_ set to 0). For both models, a weight-decay term (given by the L2-norm of all parameters) scaled by hyperparameter *α* was included in the loss function, with qualitatively equivalent results across a range of values, including 0 (presented are 0.1 for LR and 0.001 for MLP). When trained to minimize mean squared error (as in RNNs) rather than cross-entropy, either model yielded qualitatively equivalent task behavior and internal representation (the subtractive solution, S2E and S2F Fig).

### RNN variants

To identify multiple biologically relevant neural implementations of TI, two classes of RNN variants were studied:

First, RNN variants that differed in learnable connectivity: f-RNN and r-RNN. f-RNNs (fully-trainable RNNs) were RNNs where all connection weights (feedforward and recurrent) were trainable; r-RNNs (recurrent-trainable RNNs) were RNNs for which only recurrent weights (**J**), not feedforward weights (**B** and **W**), were allowed to be modified from their initial random values (Gaussian draws) in training; ff-RNNs (feedforward-trainable RNNs) were RNNs for which only feedforward weights (**B** and **W**), not recurrent weights (**J**), were modifiable in training. In addition, we found that r-RNNs for which feedforward output weights (**W**), but not feedforward input weights (**B**), were modifiable showed similar results to r-RNNs; since these networks were a relatively closer point of comparison to f-RNNs, these networks were analyzed and presented in S6A Fig (last row; r-RNNs with trainable output weights) and S8 Fig.

Second, RNN variants that differed with respect to initial connectivity strength (prior to training) and regularization—termed “constraint regime” and defined for five classes: “highest”, “high”, “intermediate”, “low”, and “lowest” (hyperparameter values in Table 1). The hyperparameter values were chosen to enable comparisons between networks differing in either initial connectivity strength (*h*_0_ and *g*_0_) or regularization (*α* and *β*) (e.g. low vs. lowest differ in initial connectivity strength); values were also chosen to be similar to those in previous work [48, 67, 72]. In pilot work, we also observed that hyperparameter values beyond the ranges presently used tended to yield models showing unrealistic behavior (e.g. failures to generalize).

In reporting results, we use the terms “higher-regime” to refer to both highest and high constraint regimes, and “lower-regime” to refer to both low and lowest constraint regimes. For the main study findings, ten RNN variants were evaluated: two types of learnable connectivity variants (f-RNN and r-RNN) by five types of constraint regime variants.

For each RNN variant (e.g. highest-constraint f-RNN), a collection of separately trained individual instances (different random initializations) were studied. In particular, we trained 200 instances of each RNN variant, subsequently studying only those models that performed TI perfectly (correct responses to all trial types) under noise-free conditions (*η*_*i*_ set to 0). This subset of instances were then subject to behavioral and neural analyses.

### Behavior simulation and behavioral patterns

To investigate behavioral patterns across models (Figs 3, 4 and 7, and S2, S3, S4, S5, S10 and S11 Figs), models were simulated on all 42 trial types (12 training and 30 test, Fig 1B). To simulate average levels of performance that were realistic to living subjects performing TI and showing characteristic behavioral patterns (>50% performance in training trials and <100% performance on trial types with large symbolic distance; see example monkey data in S5A Fig), we took the following approach.

#### Simulation approach

For each model instance that performed perfectly (correct responses on all trial types) under noise-free conditions, we added progressively larger amounts of intrinsic noise (*η*_*i*_) to model units (LR: readout units, MLP: hidden-layer units, RNN: hidden-layer (recurrent) units; s.d. of *η*_*i*_, increased from 0.5 to 5 in increments of 0.05 for RNNs, 1 to 128 in powers of 2 for LR/MLP) until the performance of the model (averaged over 500 simulations across all trial types) satisfied these basic performance criteria: >50% training performance for both choice 1 and 2 training trials and <96% performance on the largest symbolic distance trials (AG and GA). All RNN instances meeting these basic performance criteria were subsequently analyzed; for subsequent behavioral analysis, 500 simulations (of all trial types) at the identified noise level were run.

With the addition of noise, a subset of simulated trials (∼10%) did not meet the output activity threshold criterion (fixed at 85%) for a response (no response trials, see above). Though it is possible to use additional response criteria to estimate network responses in these trials [73], the following approach was taken for simplicity of interpretation: choice in these trials was randomly designated as either 1 or 2, and these trials were excluded from analyses of RT.

#### Symbolic distance effect

Trial types differing by the magnitude of the difference in rank between items (i.e. distance 1: AB, BA, BC, CB, CD, DC, DE, ED, EF, FE, FG, GF; 2: AC, CA, BD, DB, CE, EC, DF, FD, EG, GE; 3: AD, DA, BE, EB, CG, GC; 4: AE, EA, BF, FB, CG, GC, 5: AF, FA, BG, GB, 6: AG, GA). Schematic of trial types in Fig 3A (second column).

#### End item effect

Trial types differing by whether or not they contain end items A or G. Schematic of trial types in Fig 3A (third column).

#### “End order” effect

 Trial types for which end items (A or G) were presented 1st (item 1) vs. 2nd (item 2). Schematic of trial types in Fig 3A, last column. Note that trial types containing both end items (i.e. AG and GA) were not included. The end order effect refers the (sequential) order of item presentation, and is therefore specific to delay TI.

The end order index (Figs 4 and 8) was defined as:

$$\begin{array}{c} Index=\frac{RT_{1st}-RT_{2nd}}{RT_{1st}+RT_{2nd}} \end{array}$$

where *RT*_1*st*_ is the average RT over trials where end items were presented first (item 1), and *RT*_2*nd*_ is the average RT over trials where end items were presented second (item 2).

### Visualization of population activity

To clarify the implementation of TI in neural models, population-level activity was visualized by performing PCA and plotting activity in the top PCs. For each model, PCA was performed for two sets of activity: (1) a comprehensive set (presented in Fig 5A and S4C, S4D and S8A Figs), comprised of activity across all time points and trial types (all 12 training and 30 testing types; % variance explained in S3B Fig) or (2) at the end of the delay (presented in Fig 6, S9A and S9C Fig), comprised of activity from the final time step of the delay period (7 trial types corresponding to the rank of item 1, i.e. A, B, C, etc). Activity set (2) was visualized to clarify geometric relationships between trial types in the delay, when differences in activity are solely determined by the identity of item 1 (i.e. rank of item 1). All activity vectors were taken under noise-free simulations (*η*_*i*_ set to 0).

### Activity axes

Visualization of population activity in RNNs performing TI (Figs 5 and 6) suggested that the underlying neural implementations were characterized by specific arrangements (in activity space) of activity states with respect to several directions (axes), each defined on the basis of different trial types in the task (visual schematic in S7A Fig).

The first was the **choice axis**, defined as the direction pointing from the activity states of Choice 1 vs. Choice 2 trial types (21 trial types each; red vs. blue, respectively, in Fig 1B) during the choice period (period following the delay, S1A Fig). The choice axis was calculated as a unit-normalized vector pointing from the mean of activity vectors across Choice 1 trial types to the mean of activity vectors in Choice 2 trial types, both for activity averaged over the first quarter of the choice period.

The **cross-condition mean** (**XCM**; yellow line in Fig 5A and S4C, S4D and S8A Figs; [74–76]), was defined and calculated as the average trajectory across all trial types. The XCM was moreover calculated in two ways: for visualizations, the XCM was calculated for every time point; for quantifications, the XCM was calculated by taking the mean neural activity across a given time window (e.g. first or last quarter of the delay period). The **XCM axis**, defined as the direction pointing from the activity states at the beginning of the delay period to the end of the delay period, was calculated as a unit-normalized vector pointing from the XCM of the first quarter of the delay period to the XCM of the last quarter of the delay period.

The **readout axis** was defined as the unit-normalized vector of weights from the recurrent units to the choice 1 output unit (**W**_**1**_, see above).

### Inference of linear dynamics

To identify dynamical components expressed in RNNs performing TI, we fit neural activity from the delay period to an unconstrained linear dynamics model ($\dot{X}=XA$; least-squares fit) from noise-free simulations (*η*_*i*_ set to 0) across all time points during the delay and across all trial types. In the delay period there are 7 trial types corresponding to each possible item 1 (A through G). The fit was performed for the top 10 PCs of delay-period activity. *R*^2^ values of the fit were relatively high (0.5–0.9; see S6A and S6B Fig, first column, for values across networks). Eigenvalues of the A matrix were subsequently plotted for each type of RNN variant (S6A and S6B Fig, columns 2–6).

### Fixed point analysis and linearization

To identify dynamical components of RNNs performing TI, we used fixed-point analysis and linear approximation methods [77–79].

Fixed-point finding was implemented using custom code in PyTorch, following an established method [77, 78]. Optimization via gradient descent (Adam optimizer) was used to identify activity states in which the speed of RNN dynamics was minimized (mean-squared error loss). The optimization was seeded using activity states from noise-free simulations of trial types in the TI task (specifically at these time points: 0, time of item 1 presentation, time of item 2 presentation, halfway through delay, the last time point, and the last timestep when trials were simulated with 100 additional timesteps), in addition to 5 batches of 50 activity state seeds, each of which were drawn randomly from activity states of trials in which item 1 and item 2 were randomly jittered in time across the trial. Each batch was optimized for 50000 epochs and stopped after 5000 epochs with no improvement in loss. Candidate FPs were those activity states for which the speed was lower than 10^−5^. Redundant candidate FPs were eliminated by requiring that, between candidate FPs, the activity of every recurrent unit differed by more than 10^−5^.

To obtain the Jacobian matrix A of linearization, two methods were used. The first was analytic (based on the weight matrix [77]); the second was numerical [80], using the function grad() in the PyTorch autograd library: at each fixed point, the function grad() was used to calculate the entries of A for the trained (and frozen) RNN (with no external input). Each approach yielded equivalent results.

### Oscillation of transitive comparison

To identify the putative oscillation associated with transitive comparison (comparison oscillation), for each RNN performing TI we detected the oscillatory mode (mode with eigenvalue having a non-zero imaginary component) having an eigenvalue nearest 0±0.5*i* on the real-imaginary plane (imaginary component in units of cycles/delay); this mode corresponds to an approximately stable oscillation of frequency 0.5 cycles/delay, and was the mode associated with transitive comparison in higher-constraint RNNs (Fig 5B–5D and S4C, S4D and S8B–S8D Figs). This detection was performed from the inferred linear dynamics matrix (with eigenvalue spectra across RNNs in S6 Fig), as this can also be performed for experimental neural data. The 2D linear subspace (plane) of the identified oscillatory mode was defined by the eigenvectors, which were orthogonalized and unit-normalized prior to being used to visualize population activity (Fig 5B, right) and to quantify model predictions regarding activity geometry (angles of task-relevant activity axes with respect to the oscillation, S7A Fig).

### Activity geometry

To quantify patterns of population-level neural activity characteristic of different neural implementations of TI, we calculated the following geometric measures (indices). These indices were calculated for delay period activity, and were based on the following groupings of task items: *S* = 7 (all items: A, B, C, D, E, F, G), *S*_*outer*_ = 6 (all items except D: A, B, C, E, F, G), *S*_*high*_ = 3 (high-rank items: A, B, C), *S*_*low*_ = 3 (low-rank items: E, F, G). Note that each item defines a trial type during the delay period; thus for delay period activity there were 7 different trial types. All indices were calculated in the top 10 PCs of delay period activity; geometric indices were measured in the full activity space of networks (N-D ambient space, reduced to top 10 PCs) to avoid assumptions or biases incurred from an intermediate step of estimating activity subspaces (such as the subspace of an oscillation). Index values were compared between networks (e.g. RNN variants); for comparison to minimally structured activity, Gaussian random vectors were substituted for every activity vector (across timesteps and trial types) and index values re-calculated 1000 times.

#### Collinearity index

To measure the degree to which population neural activity is linearly arranged in activity space (schematic in Fig 6A; characteristic of the “subtractive” solution, Fig 5F), we defined the following index

$$\begin{array}{c} Index=\frac{1}{\left(\begin{array}{c} S\\ 2 \end{array}\right)}\sum_{i=1}^{S_{outer}}\sum_{j\neq i}^{S_{outer}}\frac{|v_{i}\cdot v_{j}|}{∥v_{i}∥∥v_{j}∥} \end{array}$$

Activity state vectors (*v*) were network activity states (x) measured with respect to the cross-condition mean (XCM; yellow circle in Fig 6B schematic; see also Fig 5A). This index was measured at two time points: at the first timestep of delay (early; Fig 6B, left column) and at the last timestep of the delay (late; Fig 6B, middle column). The change over the delay (change; Fig 6B, right column) was defined as late index—early index. Similar results were obtained when index values were quantified by averaging activity over time windows (i.e. first and last quarters of the delay).

#### Ordered collinearity index

To measure the degree to which population neural activity is both rank-ordered and linearly arranged in activity space (S9B Fig), we defined the following index

$$\begin{array}{c} Index=-\frac{1}{S_{high}S_{low}}\sum_{i}^{S_{high}}\sum_{j}^{S_{low}}\frac{v_{i}\cdot v_{j}}{∥v_{i}∥∥v_{j}∥} \end{array}$$

Activity state vectors (*v*) were network activity states (x) measured with respect to the cross-condition mean (XCM; yellow circle in Fig 6B; see also Fig 5A). Note that the negative sign results in value +1 for activity conforming to rank-ordered collinearity. This index was measured at two time points: at the first timestep of delay (early delay) and at the last timestep of the delay (late delay). The change over the delay (change) was defined as late index—early index. Similar results were obtained when index values were quantified by averaging activity over time windows (i.e. first and last quarters of the delay).

#### Mean angle

To generalize the change in ordered collinearity (see above section) to other possible angular arrangements of neural activity, we measured mean angle change (schematic of mean angle in Fig 6C), defined as

$$\begin{array}{c} Index=\frac{1}{\left(\begin{array}{c} S\\ 2 \end{array}\right)}\sum_{i=1}^{S}\sum_{j\neq i}^{S}\left[\frac{v_{i,late}\cdot v_{j,late}}{∥v_{i,late}∥∥v_{j,late}∥}-\frac{v_{i,early}\cdot v_{j,early}}{∥v_{i,early}∥∥v_{j,early}∥}\right] \end{array}$$

Activity state vectors (*v*) were network activity states (x) measured with respect to the cross-condition mean (XCM), and calculated for the first timestep of the delay (early) and at the last timestep of the delay (late). Similar results were obtained when index values were quantified by averaging activity over time windows (i.e. first and last quarters of the delay).

#### Mean distance

To help distinguish between angular vs. non-angular rearrangements of activity states in activity space (during the delay period), we measured mean distance change (schematic of mean distance in Fig 6C), defined as

$$\begin{array}{c} Index=\frac{1}{\left(\begin{array}{c} S\\ 2 \end{array}\right)}\sum_{i=1}^{S}\sum_{j\neq i}^{S}[\left(x_{i,late}-x_{j,late}\right)-\left(x_{i,early}-x_{j,early}\right)] \end{array}$$

The index was calculated for network activity states (x) at the first timestep of the delay (early) and at the last timestep of the delay (late). Similar results were obtained when index values were quantified by averaging activity over time windows (i.e. first and last quarters of the delay).

#### Axis angles

To quantify angular relationships between activity patterns (S7 Fig), we calculated the relevant cosine angles (dot product magnitude) between activity axes (XCM axis, choice axis, readout axis; see above, Activity axes), and between activity axes and the plane defined by the putative comparison oscillation (see above, Oscillation of transitive comparison). The oscillation plane was defined by two vectors (plane vectors), which were the two eigenvectors of the putative comparison oscillation orthogonalized and unit-normalized.

In particular, higher-constraint RNNs performing the basic delay TI task (Fig 5 and S8 Fig) predict four angular relationships: (1) alignment (cosine angle: above random) between the choice axis and the putative comparison oscillation, (2) orthogonality (cosine angle: ∼0 or not above random) between the XCM and the putative comparison oscillation, (3) orthogonality between the XCM and choice axis, and (4) alignment between the choice axis and the readout axis. These predictions are schematized in S7A and S7D Fig.

For (1) (S7B and S7C Fig, column 1), the dot product was calculated between the choice axis and each of the two plane vectors; the cosine angle was defined as the average of the magnitudes of the two dot products.

For (2) (S7B and S7C Fig, column 2), the dot product was calculated between the XCM and each of the two plane vectors; the cosine angle was defined as the average of the magnitudes of the two dot products.

For (3) (S7B and S7C Fig, column 3), the dot product was calculated between the XCM and the choice axis; the cosine angle was defined as the magnitude of this dot product.

For (4) (S7 Fig), the dot product was calculated between the choice axis and the readout axis; the cosine angle was defined as the magnitude of this dot product.

All angles were calculated after first reducing neural activity to the top 10 (or top 2) PCs (PCs calculated from delay period activity); for the readout axis, the weight vector **W**_**1**_ was projected to these top PCs and unit-normalized. For comparison to minimally structured activity, randomly generated vectors (unit-normalized) were substituted for each activity axis and also for each oscillatory vector (subsequently orthogonalized to obtain plane vectors), from which all angles were recalculated; 1000 such randomizations were performed.

### Neural encoding

To clarify how internal activity in RNNs generated specific behaviors (e.g. RT patterns), we examined activity projections (encodings) along two behaviorally relevant activity axes: (i) the readout axis and (ii) choice axis. For (i), the encoding was defined simply as *z*_1_ (the dot product of activity and the readout axis, with a constant bias term added; see Model architecture, RNN); for (ii), the encoding was defined as the dot product of activity with the choice axis, and was calculated in the top 10 PCs of delay period activity across trial types. All activity vectors were taken under noise-free simulations (*η*_*i*_ set to 0).

To evaluate how end items (item A or G) were encoded during the delay period, we defined an index that quantifies this difference (encoding index) as the ratio of the magnitude of projected activity from the end vs. start of the delay (last vs. first time step, respectively), averaged for A and G trials. This index was calculated for activity projected along either the readout axis (presented in Fig 7) or the choice axis (presented in S10C Fig), which yielded similar results.

### Human study data

#### Participants

The human study was approved by the Institutional Review Board (IRB) at Columbia University through Columbia IRB Protocol #AAAI1488. All participants provided written informed consent for their participation in the experiment. A total of 392 Amazon Mechanical Turk participants took part in the experiment. To promote task engagement, subjects were given a bonus for correct responses (up to $5; availability of bonus stated in advance). As clarified below, a subset of subjects were not included in analyses because they failed to meet pre-established criteria for online studies; subjects were additionally restricted those based in the US within the age range of 18–36 and with an approval rate of above 90%.

#### Materials

The item panel consisted of 7 images of fractals, assigned to items A through G randomly for each subject (Fig 8A). The images were 250 × 250 pixel size and individually presented on top of a white square with a light grey frame (RGB: 200, 200, 200; square size was 265 × 265 pixel size). These stimuli were in turn presented on a grey background (RGB: 128, 128, 128).

#### Task comprehension

Prior to the task, subjects were informed of the basic task format (i.e. presentation of two images, selection of item 1 vs. item 2 images via key presses, ability to choose once item 2 appears and during the choice period) and given a comprehension quiz. Once subjects answered all quiz questions correctly, the task began. Participants were not informed of the underlying relationship between items, or of any underlying difference in trials as the task progressed, specified as follows.

#### Trial structure and responses

Following the trial structure in neural models (S1 Fig), each trial consisted of three periods: rest (1 sec), delay (1 sec), and choice (up to 2 sec) (a schematic of trial structure in Fig 8B). The trial began with the presentation of item 1 for 250 ms. A fixation cross (“+”) was then presented for 2 sec, followed by the presentation of item 2 for 250 ms. Next, a choice screen was shown wherein the phrase “make a choice” was presented for up to 750 ms. Participants were instructed to choose one of the items by pressing either the “D” or “K” keys (randomly assigned for each participant to item 1 and 2, or *vice versa*). Participants were instructed in advance that they could respond (make a choice) from the onset of the second item until the end of the choice screen. If participants made a choice during the presentation of item 2, item 2 was nonetheless shown for its full duration (250 ms), but the choice screen was not subsequently shown in that trial. A response made during the choice screen terminated the choice period/screen; immediately thereafter, a screen indicating the outcome of their choice (green check: correct response; red cross: incorrect response) for 1 sec. If participants did not respond during the allotted time, they were shown a screen displaying “too slow” for 1 sec, and were not shown the outcome screen; these trials were treated as incorrect trials in calculating performance (% correct of all trials). In trials in which subjects responded, the response time (RT) was measured as the time elapsed from the onset of presentation of item 2. The next trial automatically began after a rest period of 1 sec.

#### Task phases and trial blocks

The task consisted of two phases, the training phase and testing phase. The training phase consisted of 144 trials divided into 3 blocks of 48 trials each, with these trials consisting of 4 repetitions of the 12 training trial types (A vs. B, B. vs. C, etc.), presented in a subject-specific random order in each block. The testing phase consisted of 252 trials divided into 6 blocks of 42 trials each, with these trials consisting of all training trial types (12 trials) and all testing trial types (30 trials), presented in a subject-specific randomized order in each block. Upon completion of testing, subjects were asked by questionnaire to describe how they performed the task (“How did you decide which item to choose?” and “What strategy (if any) did you use to learn the task?”). The entire duration of the task was ∼40 min on average. It is also worth noting that the degree of training is relatively low in the present paradigm, particularly in comparison to TI task paradigms involving thousands of training trials.

#### Inclusion criteria

To ensure that subjects were attending to the task, the following inclusion criteria were imposed: (i) fewer than 20 trials in which no response was given; (ii) fewer than 20 events where participants were browsing a different window in any experimental phase (blur-focus events detected using jsPsych library [81]), and (iii) fewer than 10 failed attempts to pass the comprehension quiz. Further, to ensure that subjects had the minimal degree of task proficiency prerequisite to testing generalization, a criterion of (iv) above chance performance (>50%) on training trials (i.e. A vs. B, B vs. C, etc.) in the final training block (block 3, Fig 8C and 8D was imposed. In an initial evaluation of inference in the delay TI paradigm, in place of (iv) a more stringent criterion of >80% performance on training trials in the final training block was imposed, similar to previous studies that initially establish TI task paradigms [82, 83]. In all main analyses (Fig 8 and S5 Fig, only subjects meeting criteria (i)-(iv) were included.

### Statistical tests

All statistical tests were non-parametric and two-sided.

## Supporting information

S1 Fig Delay TI: Transitive inference (TI) with a requirement for working memory. **A**, Trial structure. Each trial consists of three periods: rest, delay, and choice. The duration of the delay was 2*τ* to 6*τ* and either of fixed or variable length. Note that subjects respond on the basis of item order: if the correct response in trial type X vs. Y (item 1: X, item 2: Y) is choice 1, then the correct response in trial type Y vs. X (item 1: Y, item 2: X) is choice 2. **B**, Target values of RNNs output units (*z*_*i*_(*t*, *m*), where t is time and m is trial type; see Methods).
(TIF)

S2 Fig Traditional TI in feedforward models. **A**, Schematic of feedforward model architecture (see Methods). **B**, Example LR and MLP model instances that perform traditional TI (i.e. no explicit delay between items, with choice made on basis of position (left vs. right); Fig 1B). **C**, Schematic of behavior patterns. **D**, Behavior of feedforward models (n = 100 instances / model). All plots show average performance (proportion correct, averaged across 500 simulations of every trial type). Column 1: Averages across model instances by trial type. Columns 2–4: Averages across trials for each model instance by trial type. Trial types follow that defined for each behavioral pattern in panel C (column 2: symbolic distance; column 3: end item), in addition to distinguishing between choice 1 vs. choice 2 trial types (red vs. blue, respectively; diagramed in panel C, transitivity). **E** and **F**, feedforward models express a ‘subtractive’ solution to TI. **E**, Analysis of an example LR. At left, activation of readout ‘units’ (see Methods) as a function of input position (y-axis) and rank (x-axis). At right, relationship between position of inputs and readout unit activation. Note that activations by item position (left vs. right) were sign-inverted versions of each other. **F**, Analysis of an example MLP. At left, activation of hidden units as a function of input position (y-axis) and rank (x-axis). At right, relationship between position of inputs and unit activation, plotted for all hidden units (N = 100 tanh units). Note that activations by item position (left vs. right) were approximately sign-inverted versions of each other, akin to the LR model.
(TIF)

S3 FigDelay TI in RNNs: Additional examples and results.**A**, Six example RNNs that responded correctly in training trials, but failed to generalize. Plotted are network outputs by trial type (compare to Fig 2, top row, plotting conventions shared). **B**, PCA cumulative % variance explained across RNNs. Mean ± s.d. (n = 65–200 instances / variant; see Table 2 for numbers of instances; only instances that fully generalized were included). **C**, Performance (proportion correct) as a function of delay length. RNNs were trained on three delay variants: basic, extended, and variable (see Methods, Model input). Performance was measured when trials were shortened relative to the fixed (basic and extended) or maximal (variable) delay length, and performance was measured separately for training (purple) vs. test (grey) trial types. Plots show averages (dark traces) ± s.d. (shaded regions) across model instances (see S1 Table for model counts). **D**, Output activity in example RNNs (same RNNs in Fig 2). The delay and choice periods correspond to times 0 to 1 and 1 to 2, respectively. Plotted is activity of the output and saturating output corresponding to choice 1 (*z*_1_ and ${\tilde{z}}_{1}$, respectively; see Methods) under noiseless conditions. In plots of saturating output, the response threshold (85%) is indicated as a dotted grey line. Trial types are indicated by color (rank of item 1: green shade (A (dark green) to G (light grey)); choice 1 vs. 2: red vs. blue, respectively; symbolic distance: light to dark shading (1 to 6)).(TIF)

S4 Fig Delay TI in RNNs: Additional RNN variants. **A**, Behavioral patterns in highest-constraint r-RNNs across delay variants. Plotting conventions are the same as in Fig 3. **B**, End order behavior across delay variants (see Methods). Plotting conventions are the same as in Fig 4, with x-axis range (-1 to +1) made equal across plots to aid comparison. **C**, Neural activity in an example f-RNN performing the extended delay TI task. The network was trained in the highest constraint regime. Plotting conventions follow that of Fig 5A and 5B. **D**, Neural activity in an example f-RNN performing the variable delay TI task. The network was trained in the high constraint regime. Plotting conventions follow that of Fig 5A and 5B. Note the expression of an oscillation of frequency ∼0.5 cycles / delay in either network.
(TIF)

S5 Fig RNNs show TI behavior similar to that of living subjects. Comparison of behavior across trial types: living subjects and models. The behavioral data plotted are similar to that of Fig 3, but here more explicitly show differences across trial types. **A**, Monkey performance in traditional TI (items presented simultaneously, Fig 1C) by trial type. Trial types defined solely by rank of items and not order (by symbolic distance, 1: AB, BC, CD, DE, EF, FG; 2: AC, BD, CE, DF, EG; 3: AD, BE, CF, DG; 4: AE, BF, CG; 5: AF, BG; 6: AG). Originally reported in [51, 52]. **B-F**, Human and RNN performance and response times (RTs) in delay TI by trial type (n = 292 human subjects; see S1 Table for numbers of RNN instances). In delay TI, trial types depend additionally on order (AB, BA, BC, CB, etc). Plotted are average performance (top row) and RTs (bottom row; not available in feedforward models); trial types in each plot, from left to right for each symbolic distance, are at the bottom of panel B, with the distinction of choice 1 vs. 2 trials (red and blue, respectively). Highlighted in each plot are ‘critical’ trial types (testing trials that do not contain end items (A or G); yellow zones). **C**, Feedforward models (LR and MLP; n = 100 instances / model type). **D-F**, RNNs (**D**, **E**, and **F** corresponding to three delay variants: basic delay, extended delay, and variable delay; columns: f-RNN/r-RNN and highest/lowest (constraint) regimes). Error bars are ± 1 s.e.m. (monkey and human subjects) and ± 2 s.e.m. (all models).
(TIF)

S6 Fig RNNs performing delay TI: Linear dynamics. RNN activity during the delay period was fit to a linear dynamics model (least-squares). Rows show results for RNN variants differing by learnable connectivity (f-RNN: fully-trainable RNN (all weights trainable), r-RNN: recurrent-trainable RNN (only recurrent weights trainable), ff-RNN: feedforward-trainable RNN (only feedforward weights trainable), r-RNN with trainable output weights). Column 1: *R*^2^ values of the fit. Constraint regime variants plotted by color. Columns 2–6: eigenvalue spectra (grey points; calculated for each RNN instance using top 10 PCs; numbers of instances reported at bottom right), with each column corresponding to a different RNN variant (higher to lower constraint regime, indicated by color). **A**, RNNs trained on basic delay TI. Note that the spectra shown in Fig 5C corresponds to two of the spectra here combined (f-RNN highest (black, column 1) and high (blue, column 2)), and that spectra shown in S8C Fig is the same as that shown in row 3, column 1. **B**, RNNs trained on extended and variable delay TI.
(TIF)

S7 Fig RNNs performing delay TI: Geometric alignments. **A**, Schematic of putative activity geometry expressed in RNNs (compare to Figs 5A and S8A; see text for definition of activity axes). The oscillation plane refers to the putative oscillatory mode associated with transitive comparison. Note that higher-constraint RNNs trained on basic delay format (Figs 5 and S8) make three predictions: (1) the oscillation plane should be aligned with the choice axis (cosine angle: above random), (2) the oscillation plane should be orthogonal to the XCM axis (cosine angle: ∼0 or not above random), and (3) the choice axis should be orthogonal to the XCM axis (cosine angle: ∼0 or not above random). All measures were calculated from neural activity under noiseless conditions. XCM: cross-condition mean. **B**, Quantification of geometric alignment (cos angle: 1 (fully aligned), 0 (orthogonal)) in RNNs trained on basic delay TI. For comparison, values obtained between random activity vectors are shown (light grey). The quantification clarifies the predictions schematized in panel A; for summary of predictions, see Table 3. Rows: connectivity variants (f-RNN: fully-trainable RNNs, r-RNN: recurrent-trainable RNNs); columns: activity angle. **C**, Same quantification as panel B, for RNNs trained on extended- and variable-delay TI. **D**, Schematic (left) and quantification (right) of alignment of the readout axis with the choice axis. Note that the axes are consistently aligned (cos angle: above random) across all RNN variants (rows: connectivity variant; columns: delay variants). We also observed that for nearly all RNNs performing TI (98% of all instances), output units showed full separation between choice 1 vs. 2 (across all trial types) within the first ∼10% of the choice period (equivalent to ∼10% of the delay period); see S3D Fig for example output activity. Numbers of instances for each RNN variant are reported in S1 Table.
(TIF)

S8 Fig A single oscillation can enable TI in r-RNNs. **A**, Population activity trajectories in an RNN (highest-constraint r-RNN) that performs TI. Top and bottom plots show two different views. Shown are trajectories from all 42 trial types (Fig 1B). To clarify the operation of the network, three trial times are highlighted as follows: (i) presentation of item 1 (green circles; shade indicating item rank: A (dark green) to G (white)), (ii) the last time point of the delay period (green stars; same color convention), (iii) last time point of the trial (red/blue symbols; red: choice 1 trials, blue: choice 2 trials, light to dark shading indicating symbolic distance (1 to 6); diamonds: training trials, triangles: test trials). Also shown: cross-condition mean (XCM; the average trajectory across all trial types) (yellow line) and fixed point (FP) (orange cross). The FP was located near trajectories during the delay period (‘early-trial’ FP, compare to Fig 5A). Note the oscillatory evolution of trajectories in the delay period (circles to stars) despite the absence of a linearly arranged rank-ordered activity upon presentation of item 1 (green circles; compare to Fig 6A). **B**, Linear dynamics of RNN in panel A. Two eigenvalue spectra of the RNN are plotted: first, the spectrum calculated from delay-period neural activity (black points; inferred via least-squares linear fit, *R*^2^ = 0.78) and second, the spectrum from linearization of the network with respect to the early-trial FP (orange circles; FP shown as orange cross in panel A). **C**, Linear dynamics of higher-constraint f-RNNs (n = 200 instances, highest regime). Eigenvalue spectra of delay-period neural activity (grey translucent points; inferred via least-squares linear fit, *R*^2^ ∼ 0.8 across the 200 instances, see S6A Fig, row 3). Note the density of oscillatory modes with frequency ∼0.5 cycles / delay (filled arrowhead; compare to Fig 5C). **D**, Activity trajectories in the oscillatory mode of the linearized RNN. The oscillatory mode is that of the linearization of the early-trial FP (open arrowheads in panel B). Two sets of trajectories are plotted (rows): one where all activity (initial conditions and inputs) has been reduced to the 2 dimensions of the oscillatory mode (top row, reduced) and one where the full dimensionality (N-D; here N = 100) of initial conditions and inputs have been retained (bottom row, full). In both cases, the dynamics are governed solely by the oscillatory mode (see Methods), though only in the reduced (2D) case do trajectories strictly follow the flow field vectors. To clarify the progression of activity, trajectories are plotted in two stages of the task trial (left and right columns; schematic of the stages at the bottom of each panel): early trial (left) and late trial (right). To clarify how the activity evolves, specific trial times are highlighted as follows: (i) presentation of item 1 (green circles; shade corresponding to item rank: A (dark green) to G (white)), (ii) the last time point of the delay period (green stars; same color convention), (iii) a quarter of the time period following presentation of item 2 (i.e. choice period, see S1 Fig; red/blue symbols; red: choice 1 trials, blue: choice 2 trials; diamonds: training trials, triangles: test trials). Note that separation (linear separability) of choice 1 vs. 2 trials (red vs. blue symbols) does not occur in the reduced-activity case, but does occur in the full-activity case.
(TIF)

S9 Fig RNNs performing delay TI: Activity geometry during the delay. **A,** Delay period population activity in 12 additional example RNNs. Plotting conventions follow that of Fig 6A. In instances of lowest-constraint RNNs, a “V” activity geometry was expressed by the end of the delay period (late delay; highlighted with red box). In the instances shown, the mean angle change index values were 0.04 (top row, f-RNN lowest), 0.02 (bottom row, f-RNN lowest), and 0.05 (middle row, r-RNN lowest). **B**, Histograms of geometric index values across RNNs. Plotting conventions follow those of Fig 6B and 6C, with the addition of ordered collinearity (see Methods) and with the same analysis carried out in the top 2 PCs (at bottom). All plots show histograms of instances for each RNN variant (n = 65–200 instances / variant; see Table 2), in addition to randomly generated data (open grey histograms). **C**, Two geometric indices for four example RNNs. Each example (column sections) is from panel A (f-RNN highest, f-RNN lowest, f-RNN lowest, r-RNN lowest). At top, the delay period population activity (PC1 and PC2; early delay (left) and late delay (right)) is shown. At bottom, geometric index values are shown, calculated in the top 10 and top 2 PCs. Note that the second through fourth examples show “V” shaped geometry in late delay, and further have positive mean angle change values. **D**, Collinearity over the course of the delay period in eight example RNNs (two examples / variant; variant indicated above). In each plot, two measures are plotted: the collinearity index (black lines; schematized in Fig 6B) and individual cosine angles between trial types (grey lines; e.g. A vs. B, B vs. C activity states). The collinearity index is the average across cosine angles.
(TIF)

S10 Fig RNNs performing delay TI: Activity geometry and encoding strategy predict behavior. **A**, End order behavior vs. activity geometry across RNN variants. Behavior (y-axis) is the end order pattern (Figs 3 and 4; quantified by the end order index; see Methods), for which RNNs show alternative versions (1st vs. 2nd-faster; >0 and <0 index values, respectively). Activity geometry (x-axis) correspond to the patterns schematized and quantified in Fig 6. **B**, End order behavior vs. activity geometry across all RNNs in the present study. Each row corresponds to a different delay variant (see Methods for details). Plots contain the same data as in panel A, but do not show constraint regime. Note that for mean angle change, alternative behaviors (index values <0 vs. >0) correspond to qualitatively different geometries (>0 vs. <0). **C**, Alternative encoding strategies in RNNs. Example networks (upper rows) and plotting conventions are the same as in Fig 7, with the difference that activity projections were on the choice axis (rather than readout axis).
(TIF)

S11 Fig End order effect: RNNs vs. human data. To compare model behavior to that of human subjects, the Wasserstein distance (earth mover’s distance) was calculated between the end-order index values across network instances for each RNN variant (Figs 4B and S4B) to end-order index values across human subjects (Fig 8G). Several RNN variants (variable delay r-RNN in intermediate, low, and lowest constraint regimes) are omitted due to insufficient number of network instances.
(TIF)

S12 Fig Word count summary of human subject responses. Word count summary (generated using wordcounter.ai) of typed responses to a question (“How did you decide which item to choose?”) in debriefing questionnaire in the human behavioral study of the delay TI task.
(TIF)

S1 Appendix The “subtractive” solution to delay TI. Diagrams presenting the solution in greater detail (compare to Fig 5). Top, diagram of each of the population-level components comprising the solution (top: the specific form of the component; bottom: the network implementation). Bottom, activity trajectories across trial periods (columns; diagram of each period at bottom) and across different trial types (rows; top row: all trials; single trial types in rows below). Trajectories were generated by simulating a 2D linear dynamical system defined by an oscillation of frequency ∼0.5 cycles / delay, with initial condition at the origin and input vectors encoding task items (A, B, C, etc.) in ordered collinear arrangement in state space. Trial-based input (item 1—delay—item 2, see S1A Fig) was applied to the system.
(TIF)

S1 Table Number of RNNs that fully generalized out of 200 trained instances. Each entry corresponds to a particular input format (basic, extended, and variable delay) and RNN variant (learned connectivity: f-RNN, r-RNN, ff-RNN; constraint regime: higher to lower, see Table 1).
(PDF)

S2 Table Responses to debriefing question. Examples of typed responses to a question (“How did you decide which item to choose?”) in a debriefing questionnaire given to subjects after completing all trials in the delay TI task. Example responses are sorted by performance on test trial types averaged over the last three blocks of trials. See S12 Fig for word count summary. Subjects performing at high levels tended to use words indicating understanding of the transitive relationship (e.g. “hierarchy”, “order”, “higher”, “ranked”). Lower-performing subjects appeared to use these words less often, rather using phrases like “random”, “tried to remember”, or referring to other strategies or a lack thereof (e.g. “didn’t have a specific strategy”, “the more attractive image”, or strategies based on a single item, “choose one and if it was right I would keep choosing the same one”). Words denoting a comparative relationship between items were commonly used (e.g. “beat”, “win”, “lost”). A number of subjects at various performance levels mentioned that some items were always correct.
(PDF)
