# Supplementary figures and images for: Emergent neural dynamics and geometry for generalization in a transitive inference task
Source: PLoS Comput Biol. 2024 Apr 25;20(4):e1011954. doi: 10.1371/journal.pcbi.1011954 (PMC11125559; doi:10.1371/journal.pcbi.1011954)

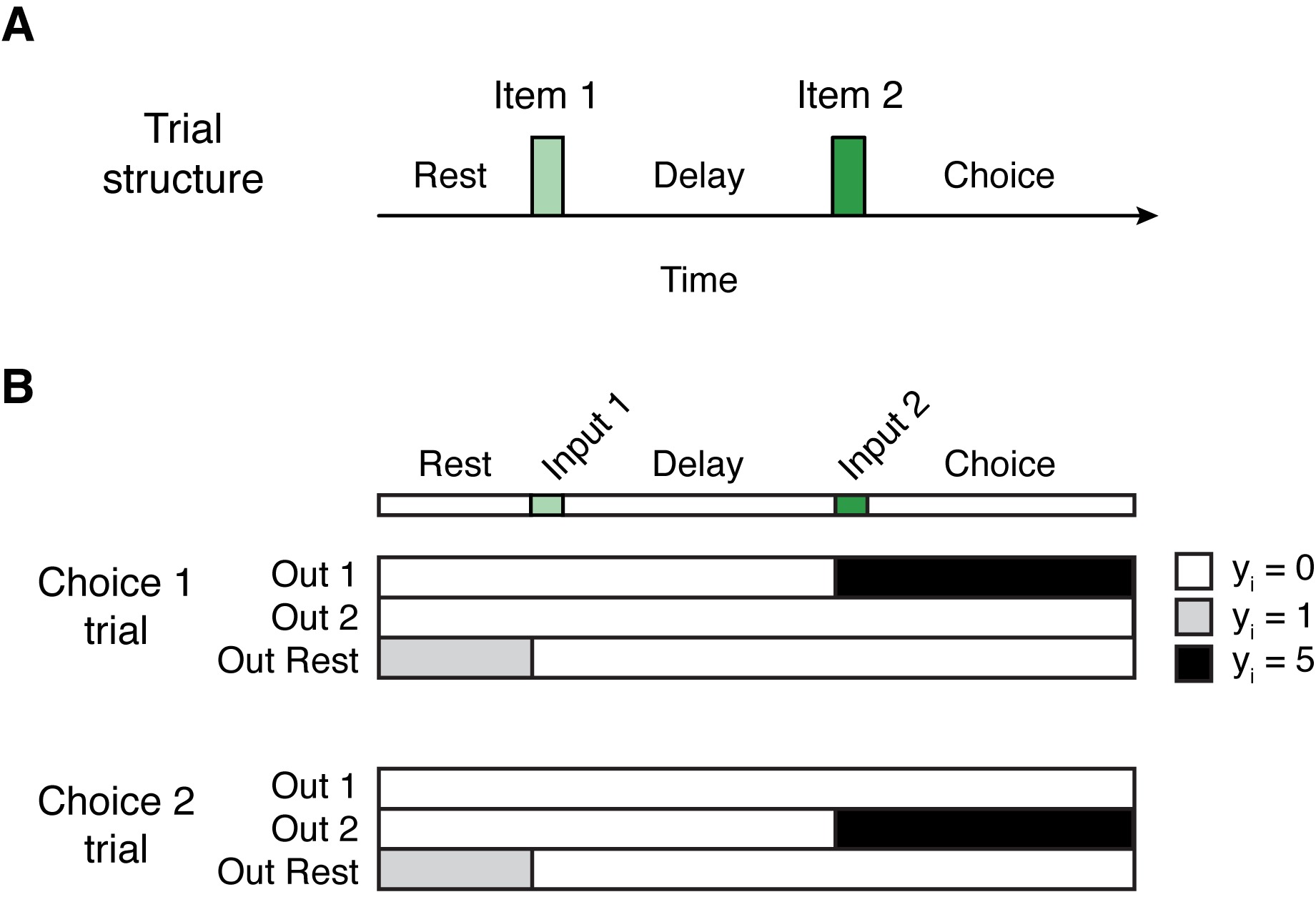

Supplement: S1 Fig — A, Trial structure. Each trial consists of three periods: rest, delay, and choice. The duration of the delay was 2τ to 6τ and either of fixed or variable length. Note that subjects respond on the basis of item order: if the correct response in trial type X vs. Y (item 1: X, item 2: Y) is choice 1, then the correct response in trial type Y vs. X (item 1: Y, item 2: X) is choice 2. B, Target values of RNNs output units (zi(t, m), where t is time and m is trial type; see Methods). (TIF) [file pcbi.1011954.s001.tif]

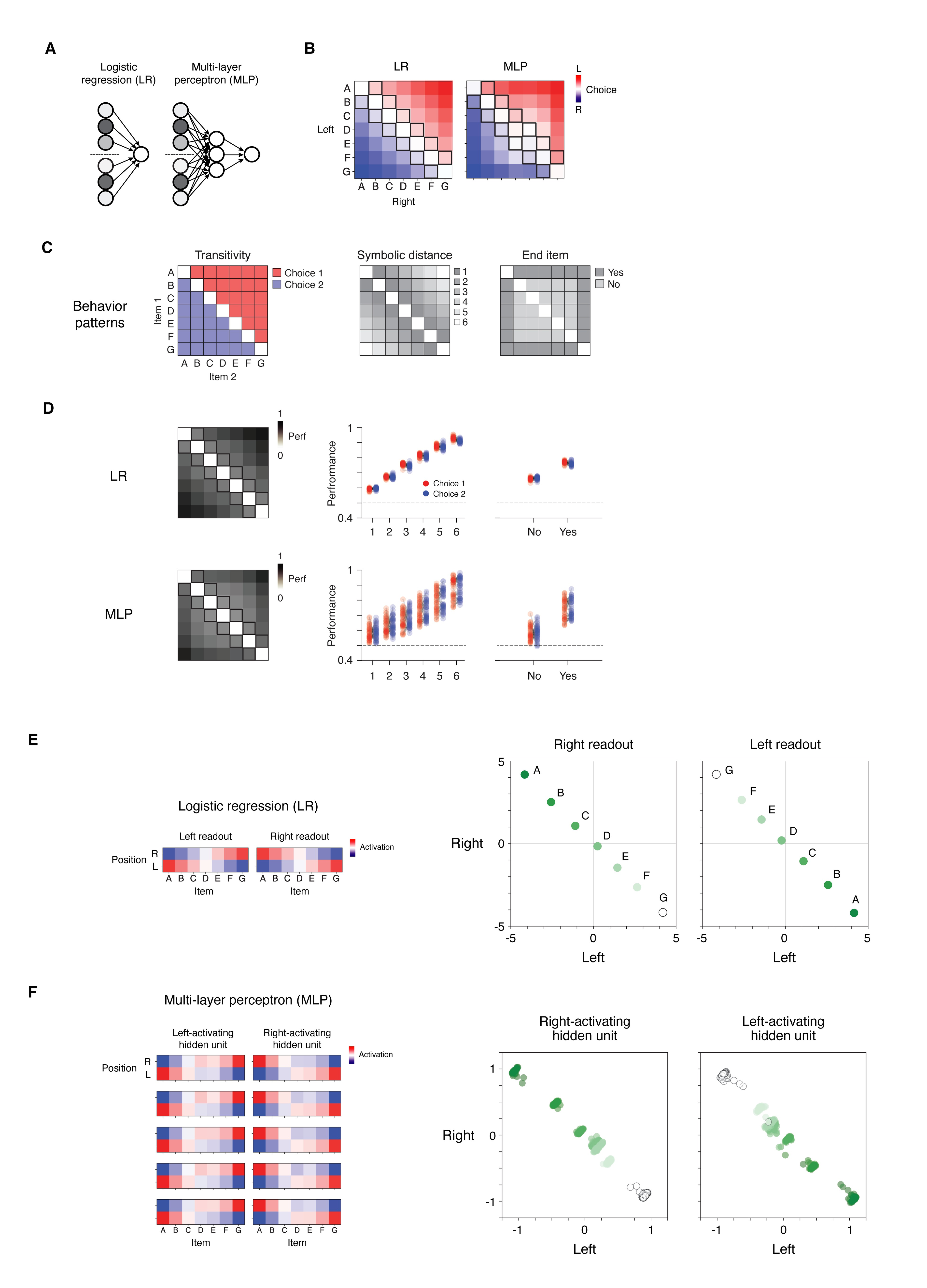

Supplement: S2 Fig — A, Schematic of feedforward model architecture (see Methods). B, Example LR and MLP model instances that perform traditional TI (i.e. no explicit delay between items, with choice made on basis of position (left vs. right); Fig 1B). C, Schematic of behavior patterns. D, Behavior of feedforward models (n = 100 instances / model). All plots show average performance (proportion correct, averaged across 500 simulations of every trial type). Column 1: Averages across model instances by trial type. Columns 2–4: Averages across trials for each model instance by trial type. Trial types follow that defined for each behavioral pattern in panel C (column 2: symbolic distance; column 3: end item), in addition to distinguishing between choice 1 vs. choice 2 trial types (red vs. blue, respectively; diagramed in panel C, transitivity). E and F, feedforward models express a ‘subtractive’ solution to TI. E, Analysis of an example LR. At left, activation of readout ‘units’ (see Methods) as a function of input position (y-axis) and rank (x-axis). At right, relationship between position of inputs and readout unit activation. Note that activations by item position (left vs. right) were sign-inverted versions of each other. F, Analysis of an example MLP. At left, activation of hidden units as a function of input position (y-axis) and rank (x-axis). At right, relationship between position of inputs and unit activation, plotted for all hidden units (N = 100 tanh units). Note that activations by item position (left vs. right) were approximately sign-inverted versions of each other, akin to the LR model. (TIF) [file pcbi.1011954.s002.tif]

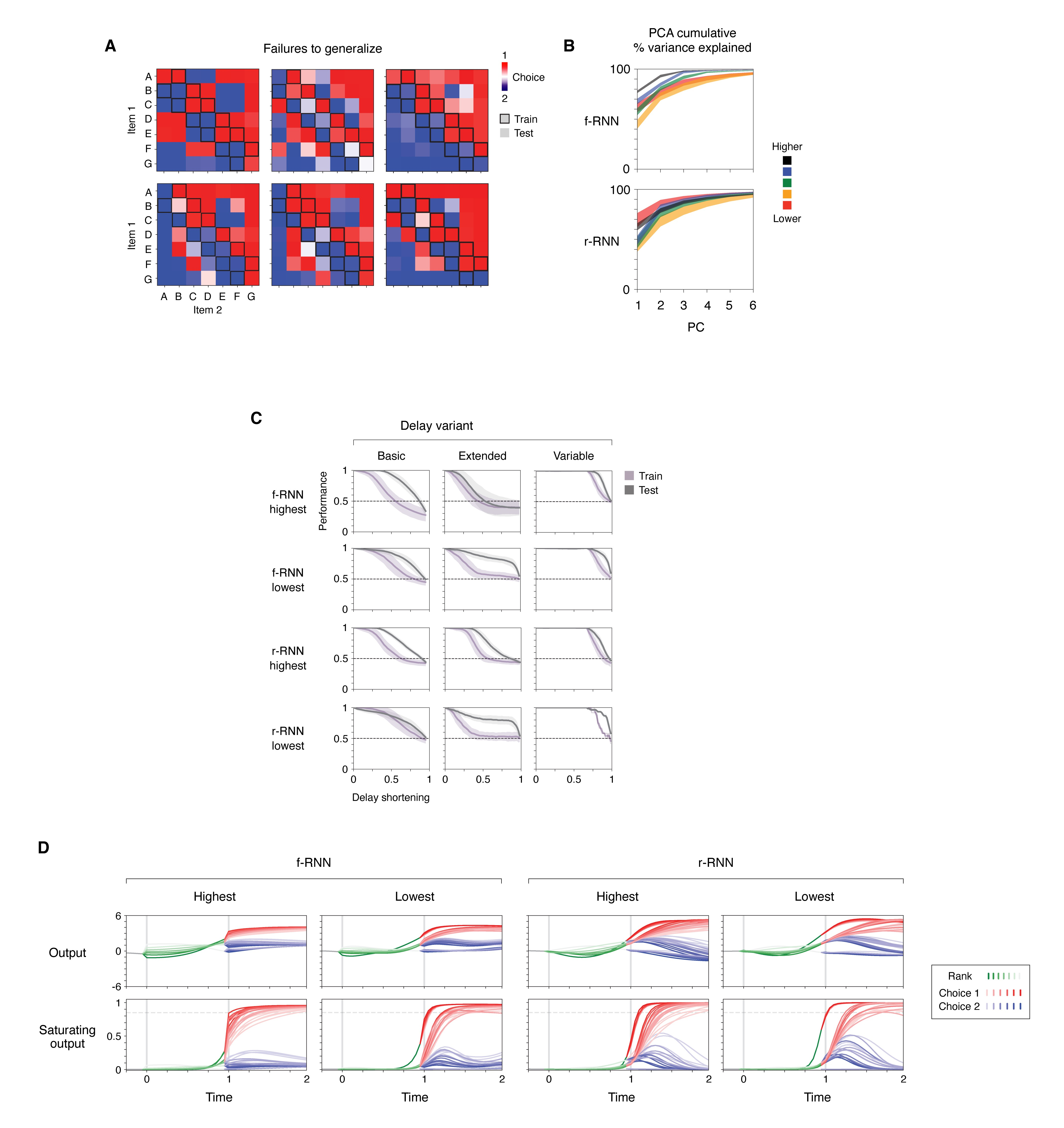

Supplement: S3 Fig — A, Six example RNNs that responded correctly in training trials, but failed to generalize. Plotted are network outputs by trial type (compare to Fig 2, top row, plotting conventions shared). B, PCA cumulative % variance explained across RNNs. Mean ± s.d. (n = 65–200 instances / variant; see Table 2 for numbers of instances; only instances that fully generalized were included). C, Performance (proportion correct) as a function of delay length. RNNs were trained on three delay variants: basic, extended, and variable (see Methods, Model input). Performance was measured when trials were shortened relative to the fixed (basic and extended) or maximal (variable) delay length, and performance was measured separately for training (purple) vs. test (grey) trial types. Plots show averages (dark traces) ± s.d. (shaded regions) across model instances (see S1 Table for model counts). D, Output activity in example RNNs (same RNNs in Fig 2). The delay and choice periods correspond to times 0 to 1 and 1 to 2, respectively. Plotted is activity of the output and saturating output corresponding to choice 1 (z1 and z˜1, respectively; see Methods) under noiseless conditions. In plots of saturating output, the response threshold (85%) is indicated as a dotted grey line. Trial types are indicated by color (rank of item 1: green shade (A (dark green) to G (light grey)); choice 1 vs. 2: red vs. blue, respectively; symbolic distance: light to dark shading (1 to 6)). (TIF) [file pcbi.1011954.s003.tif]

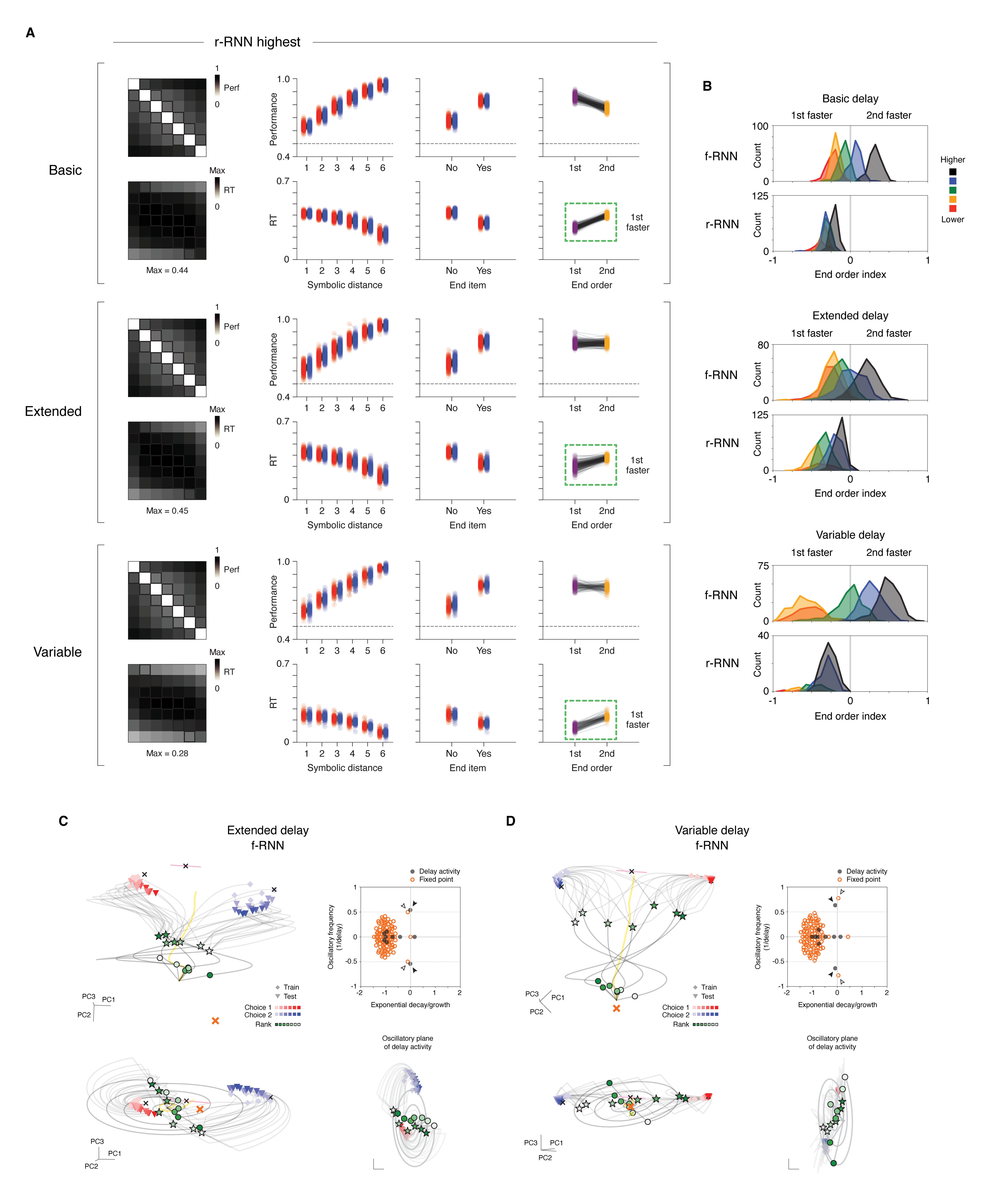

Supplement: S4 Fig — A, Behavioral patterns in highest-constraint r-RNNs across delay variants. Plotting conventions are the same as in Fig 3. B, End order behavior across delay variants (see Methods). Plotting conventions are the same as in Fig 4, with x-axis range (-1 to +1) made equal across plots to aid comparison. C, Neural activity in an example f-RNN performing the extended delay TI task. The network was trained in the highest constraint regime. Plotting conventions follow that of Fig 5A and 5B. D, Neural activity in an example f-RNN performing the variable delay TI task. The network was trained in the high constraint regime. Plotting conventions follow that of Fig 5A and 5B. Note the expression of an oscillation of frequency ∼0.5 cycles / delay in either network. (TIF) [file pcbi.1011954.s004.tif]

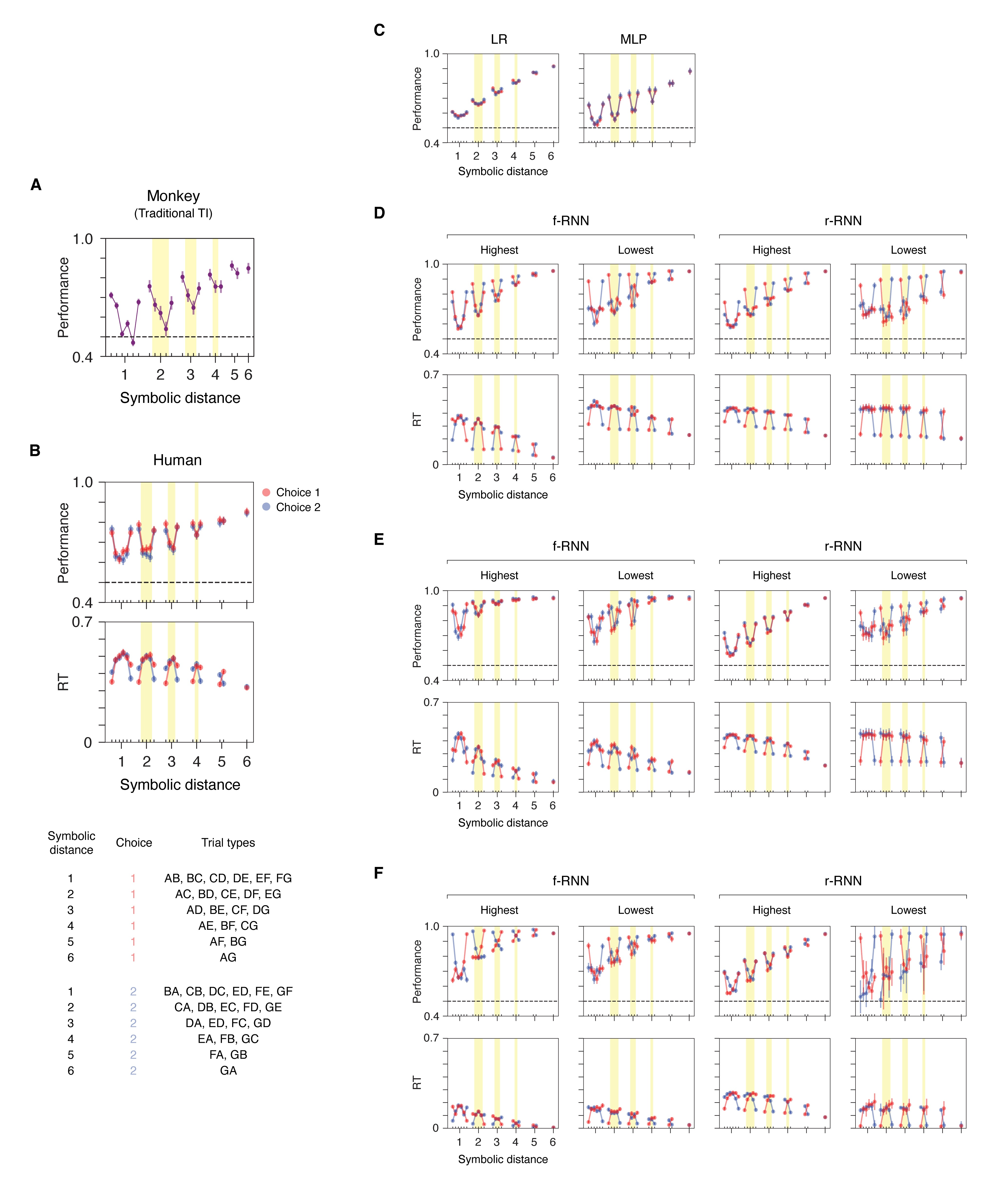

Supplement: S5 Fig — Comparison of behavior across trial types: living subjects and models. The behavioral data plotted are similar to that of Fig 3, but here more explicitly show differences across trial types. A, Monkey performance in traditional TI (items presented simultaneously, Fig 1C) by trial type. Trial types defined solely by rank of items and not order (by symbolic distance, 1: AB, BC, CD, DE, EF, FG; 2: AC, BD, CE, DF, EG; 3: AD, BE, CF, DG; 4: AE, BF, CG; 5: AF, BG; 6: AG). Originally reported in [51, 52]. B-F, Human and RNN performance and response times (RTs) in delay TI by trial type (n = 292 human subjects; see S1 Table for numbers of RNN instances). In delay TI, trial types depend additionally on order (AB, BA, BC, CB, etc). Plotted are average performance (top row) and RTs (bottom row; not available in feedforward models); trial types in each plot, from left to right for each symbolic distance, are at the bottom of panel B, with the distinction of choice 1 vs. 2 trials (red and blue, respectively). Highlighted in each plot are ‘critical’ trial types (testing trials that do not contain end items (A or G); yellow zones). C, Feedforward models (LR and MLP; n = 100 instances / model type). D-F, RNNs (D, E, and F corresponding to three delay variants: basic delay, extended delay, and variable delay; columns: f-RNN/r-RNN and highest/lowest (constraint) regimes). Error bars are ± 1 s.e.m. (monkey and human subjects) and ± 2 s.e.m. (all models). (TIF) [file pcbi.1011954.s005.tif]

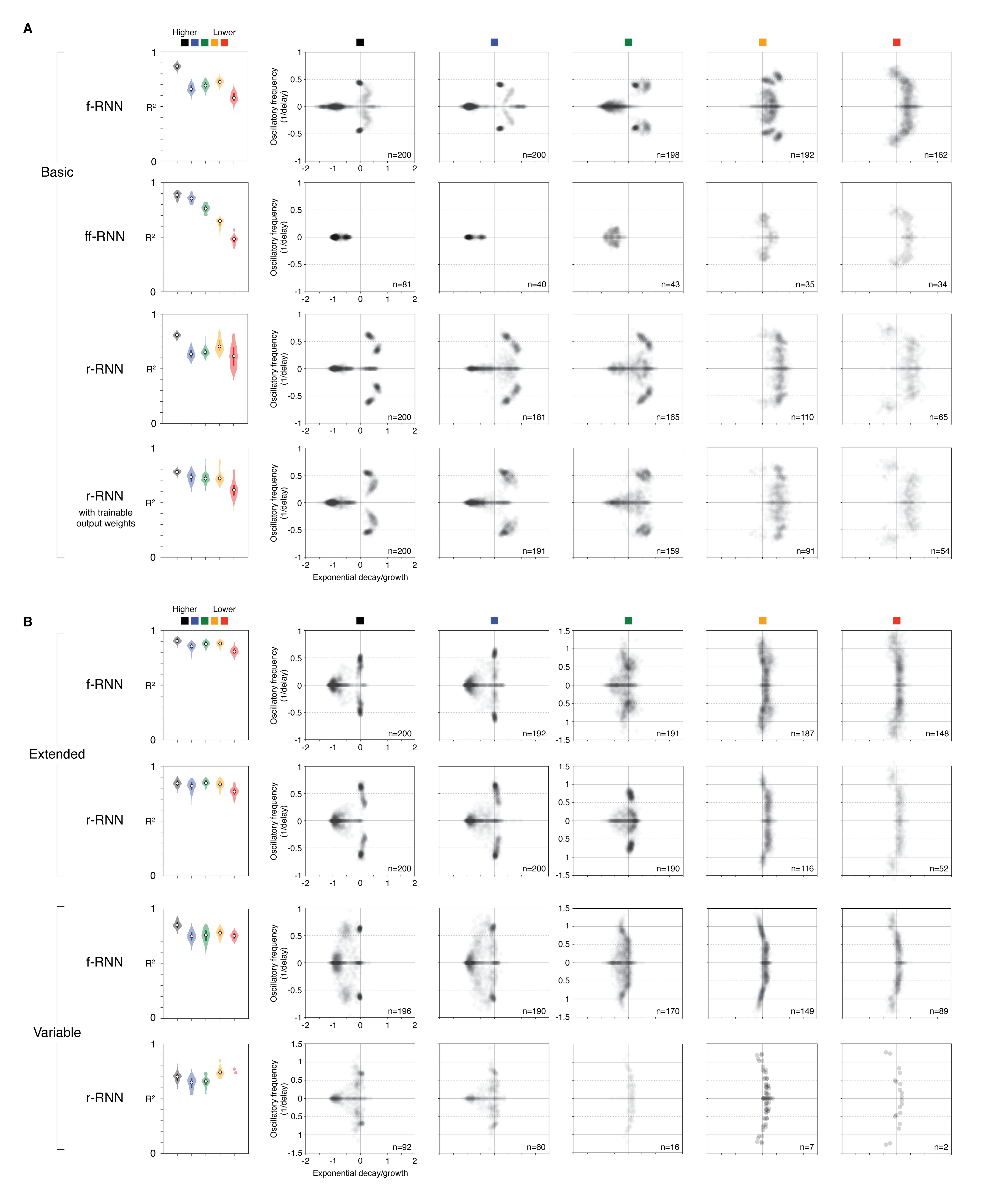

Supplement: S6 Fig — RNN activity during the delay period was fit to a linear dynamics model (least-squares). Rows show results for RNN variants differing by learnable connectivity (f-RNN: fully-trainable RNN (all weights trainable), r-RNN: recurrent-trainable RNN (only recurrent weights trainable), ff-RNN: feedforward-trainable RNN (only feedforward weights trainable), r-RNN with trainable output weights). Column 1: R2 values of the fit. Constraint regime variants plotted by color. Columns 2–6: eigenvalue spectra (grey points; calculated for each RNN instance using top 10 PCs; numbers of instances reported at bottom right), with each column corresponding to a different RNN variant (higher to lower constraint regime, indicated by color). A, RNNs trained on basic delay TI. Note that the spectra shown in Fig 5C corresponds to two of the spectra here combined (f-RNN highest (black, column 1) and high (blue, column 2)), and that spectra shown in S8C Fig is the same as that shown in row 3, column 1. B, RNNs trained on extended and variable delay TI. (TIF) [file pcbi.1011954.s006.tif]

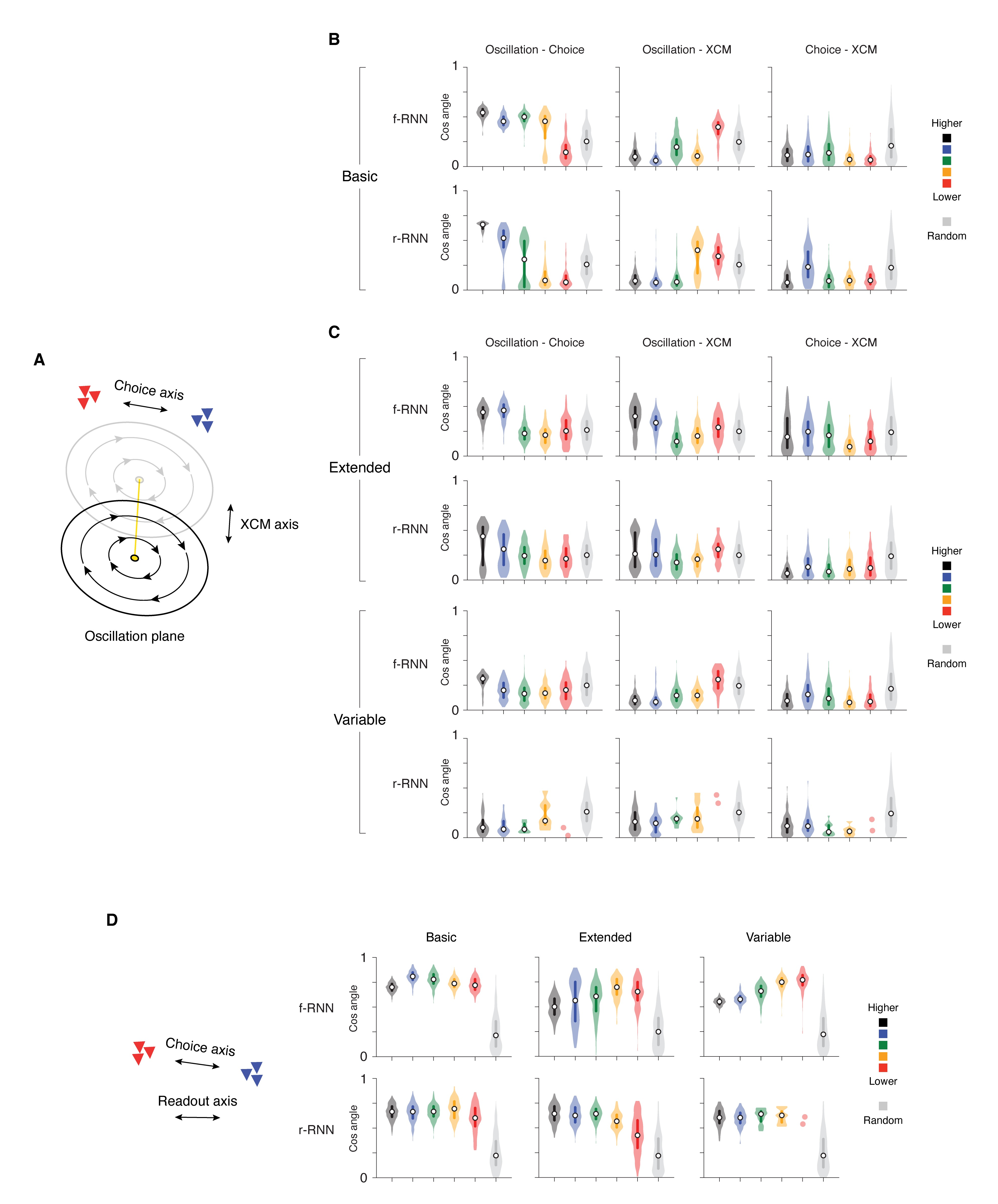

Supplement: S7 Fig — A, Schematic of putative activity geometry expressed in RNNs (compare to Figs 5A and S8A; see text for definition of activity axes). The oscillation plane refers to the putative oscillatory mode associated with transitive comparison. Note that higher-constraint RNNs trained on basic delay format (Figs 5 and S8) make three predictions: (1) the oscillation plane should be aligned with the choice axis (cosine angle: above random), (2) the oscillation plane should be orthogonal to the XCM axis (cosine angle: ∼0 or not above random), and (3) the choice axis should be orthogonal to the XCM axis (cosine angle: ∼0 or not above random). All measures were calculated from neural activity under noiseless conditions. XCM: cross-condition mean. B, Quantification of geometric alignment (cos angle: 1 (fully aligned), 0 (orthogonal)) in RNNs trained on basic delay TI. For comparison, values obtained between random activity vectors are shown (light grey). The quantification clarifies the predictions schematized in panel A; for summary of predictions, see Table 3. Rows: connectivity variants (f-RNN: fully-trainable RNNs, r-RNN: recurrent-trainable RNNs); columns: activity angle. C, Same quantification as panel B, for RNNs trained on extended- and variable-delay TI. D, Schematic (left) and quantification (right) of alignment of the readout axis with the choice axis. Note that the axes are consistently aligned (cos angle: above random) across all RNN variants (rows: connectivity variant; columns: delay variants). We also observed that for nearly all RNNs performing TI (98% of all instances), output units showed full separation between choice 1 vs. 2 (across all trial types) within the first ∼10% of the choice period (equivalent to ∼10% of the delay period); see S3D Fig for example output activity. Numbers of instances for each RNN variant are reported in S1 Table. (TIF) [file pcbi.1011954.s007.tif]

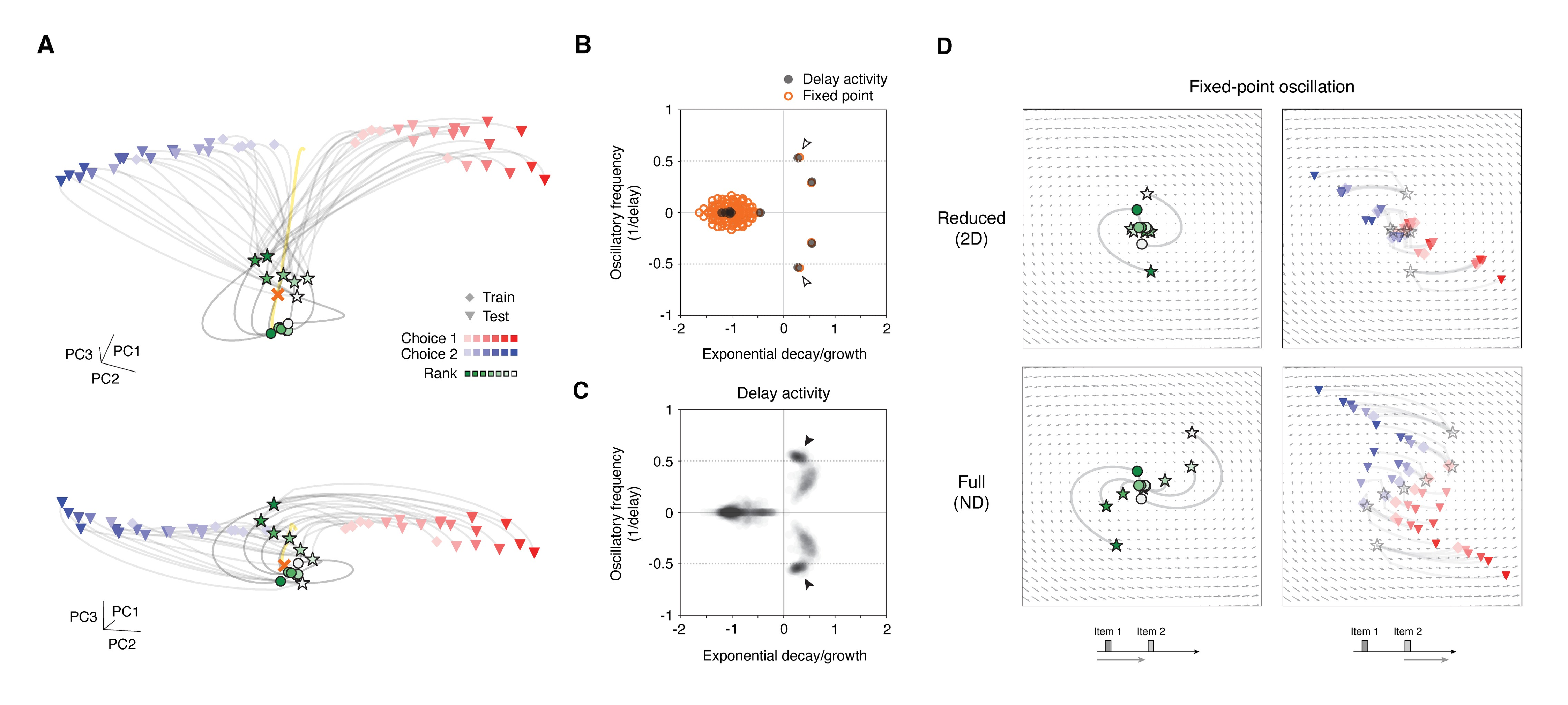

Supplement: S8 Fig — A, Population activity trajectories in an RNN (highest-constraint r-RNN) that performs TI. Top and bottom plots show two different views. Shown are trajectories from all 42 trial types (Fig 1B). To clarify the operation of the network, three trial times are highlighted as follows: (i) presentation of item 1 (green circles; shade indicating item rank: A (dark green) to G (white)), (ii) the last time point of the delay period (green stars; same color convention), (iii) last time point of the trial (red/blue symbols; red: choice 1 trials, blue: choice 2 trials, light to dark shading indicating symbolic distance (1 to 6); diamonds: training trials, triangles: test trials). Also shown: cross-condition mean (XCM; the average trajectory across all trial types) (yellow line) and fixed point (FP) (orange cross). The FP was located near trajectories during the delay period (‘early-trial’ FP, compare to Fig 5A). Note the oscillatory evolution of trajectories in the delay period (circles to stars) despite the absence of a linearly arranged rank-ordered activity upon presentation of item 1 (green circles; compare to Fig 6A). B, Linear dynamics of RNN in panel A. Two eigenvalue spectra of the RNN are plotted: first, the spectrum calculated from delay-period neural activity (black points; inferred via least-squares linear fit, R2 = 0.78) and second, the spectrum from linearization of the network with respect to the early-trial FP (orange circles; FP shown as orange cross in panel A). C, Linear dynamics of higher-constraint f-RNNs (n = 200 instances, highest regime). Eigenvalue spectra of delay-period neural activity (grey translucent points; inferred via least-squares linear fit, R2 ∼ 0.8 across the 200 instances, see S6A Fig, row 3). Note the density of oscillatory modes with frequency ∼0.5 cycles / delay (filled arrowhead; compare to Fig 5C). D, Activity trajectories in the oscillatory mode of the linearized RNN. The oscillatory mode is that of the linearization of the early-tri [file pcbi.1011954.s008.tif]

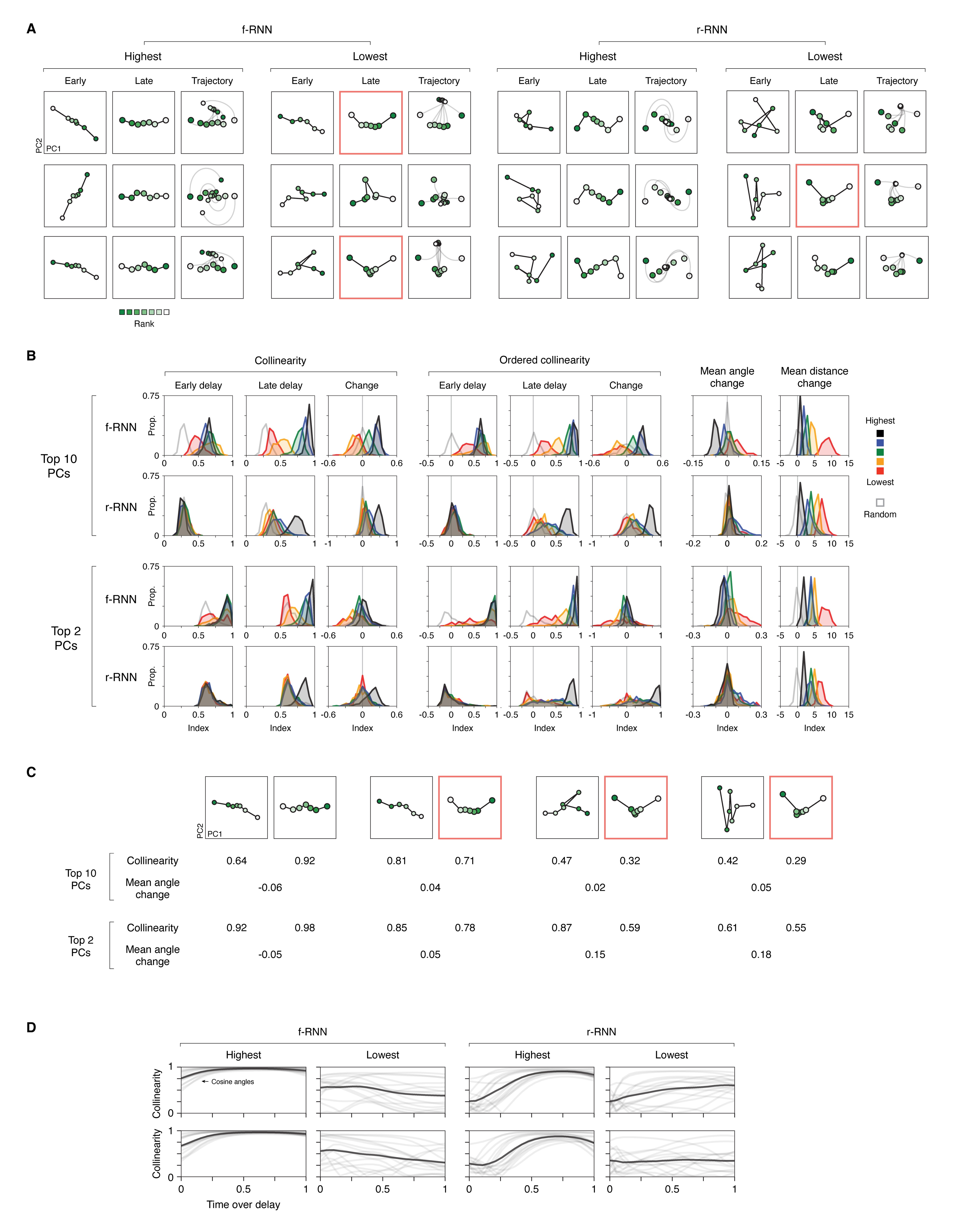

Supplement: S9 Fig — A, Delay period population activity in 12 additional example RNNs. Plotting conventions follow that of Fig 6A. In instances of lowest-constraint RNNs, a “V” activity geometry was expressed by the end of the delay period (late delay; highlighted with red box). In the instances shown, the mean angle change index values were 0.04 (top row, f-RNN lowest), 0.02 (bottom row, f-RNN lowest), and 0.05 (middle row, r-RNN lowest). B, Histograms of geometric index values across RNNs. Plotting conventions follow those of Fig 6B and 6C, with the addition of ordered collinearity (see Methods) and with the same analysis carried out in the top 2 PCs (at bottom). All plots show histograms of instances for each RNN variant (n = 65–200 instances / variant; see Table 2), in addition to randomly generated data (open grey histograms). C, Two geometric indices for four example RNNs. Each example (column sections) is from panel A (f-RNN highest, f-RNN lowest, f-RNN lowest, r-RNN lowest). At top, the delay period population activity (PC1 and PC2; early delay (left) and late delay (right)) is shown. At bottom, geometric index values are shown, calculated in the top 10 and top 2 PCs. Note that the second through fourth examples show “V” shaped geometry in late delay, and further have positive mean angle change values. D, Collinearity over the course of the delay period in eight example RNNs (two examples / variant; variant indicated above). In each plot, two measures are plotted: the collinearity index (black lines; schematized in Fig 6B) and individual cosine angles between trial types (grey lines; e.g. A vs. B, B vs. C activity states). The collinearity index is the average across cosine angles. (TIF) [file pcbi.1011954.s009.tif]

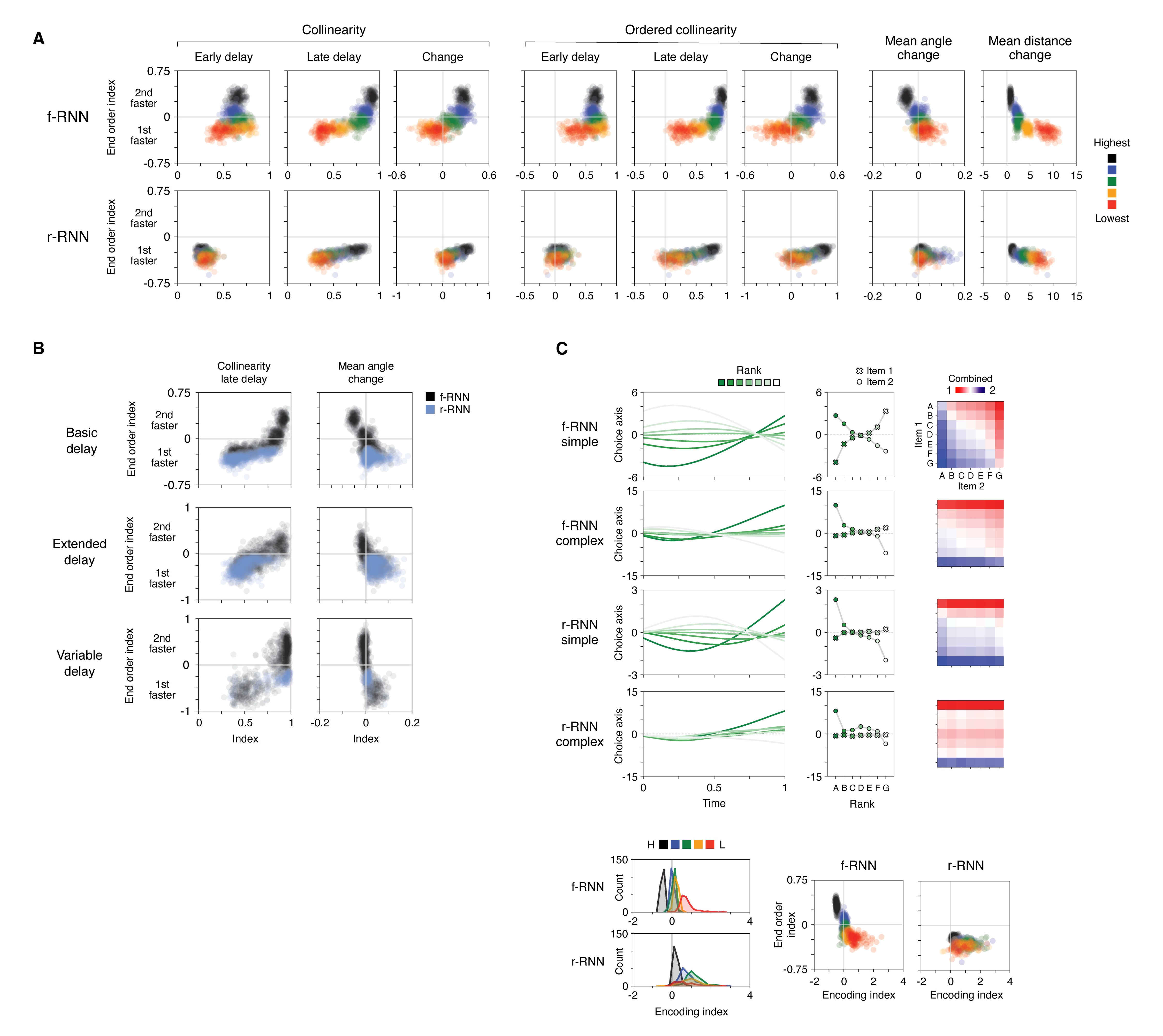

Supplement: S10 Fig — A, End order behavior vs. activity geometry across RNN variants. Behavior (y-axis) is the end order pattern (Figs 3 and 4; quantified by the end order index; see Methods), for which RNNs show alternative versions (1st vs. 2nd-faster; >0 and <0 index values, respectively). Activity geometry (x-axis) correspond to the patterns schematized and quantified in Fig 6. B, End order behavior vs. activity geometry across all RNNs in the present study. Each row corresponds to a different delay variant (see Methods for details). Plots contain the same data as in panel A, but do not show constraint regime. Note that for mean angle change, alternative behaviors (index values <0 vs. >0) correspond to qualitatively different geometries (>0 vs. <0). C, Alternative encoding strategies in RNNs. Example networks (upper rows) and plotting conventions are the same as in Fig 7, with the difference that activity projections were on the choice axis (rather than readout axis). (TIF) [file pcbi.1011954.s010.tif]

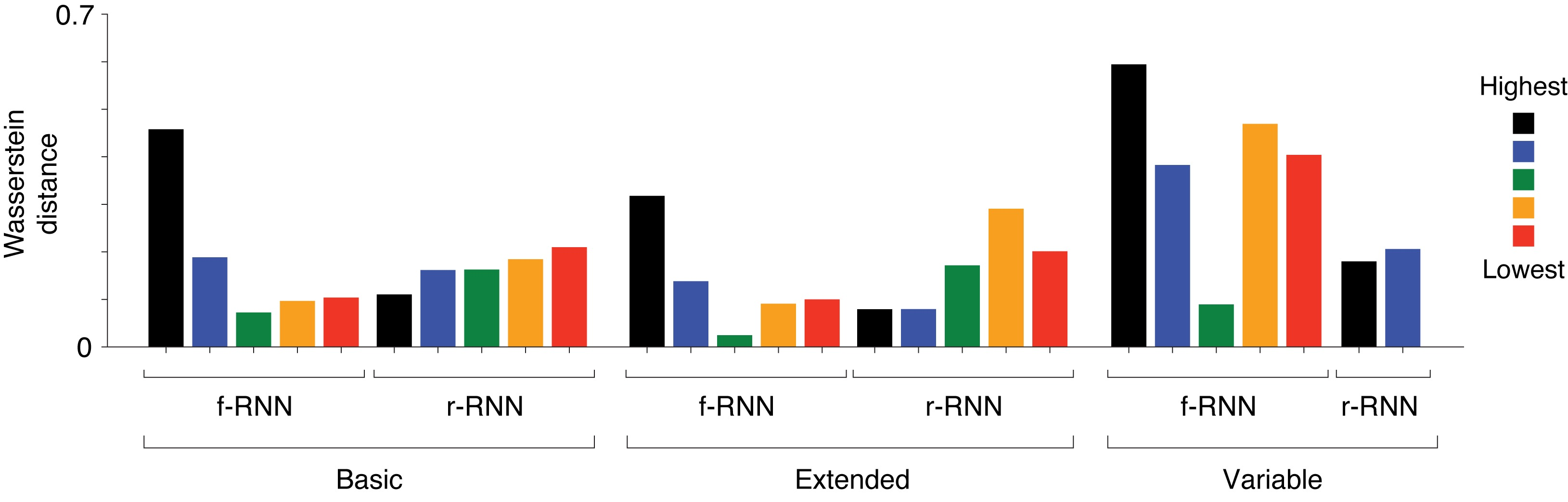

Supplement: S11 Fig — To compare model behavior to that of human subjects, the Wasserstein distance (earth mover’s distance) was calculated between the end-order index values across network instances for each RNN variant (Figs 4B and S4B) to end-order index values across human subjects (Fig 8G). Several RNN variants (variable delay r-RNN in intermediate, low, and lowest constraint regimes) are omitted due to insufficient number of network instances. (TIF) [file pcbi.1011954.s011.tif]

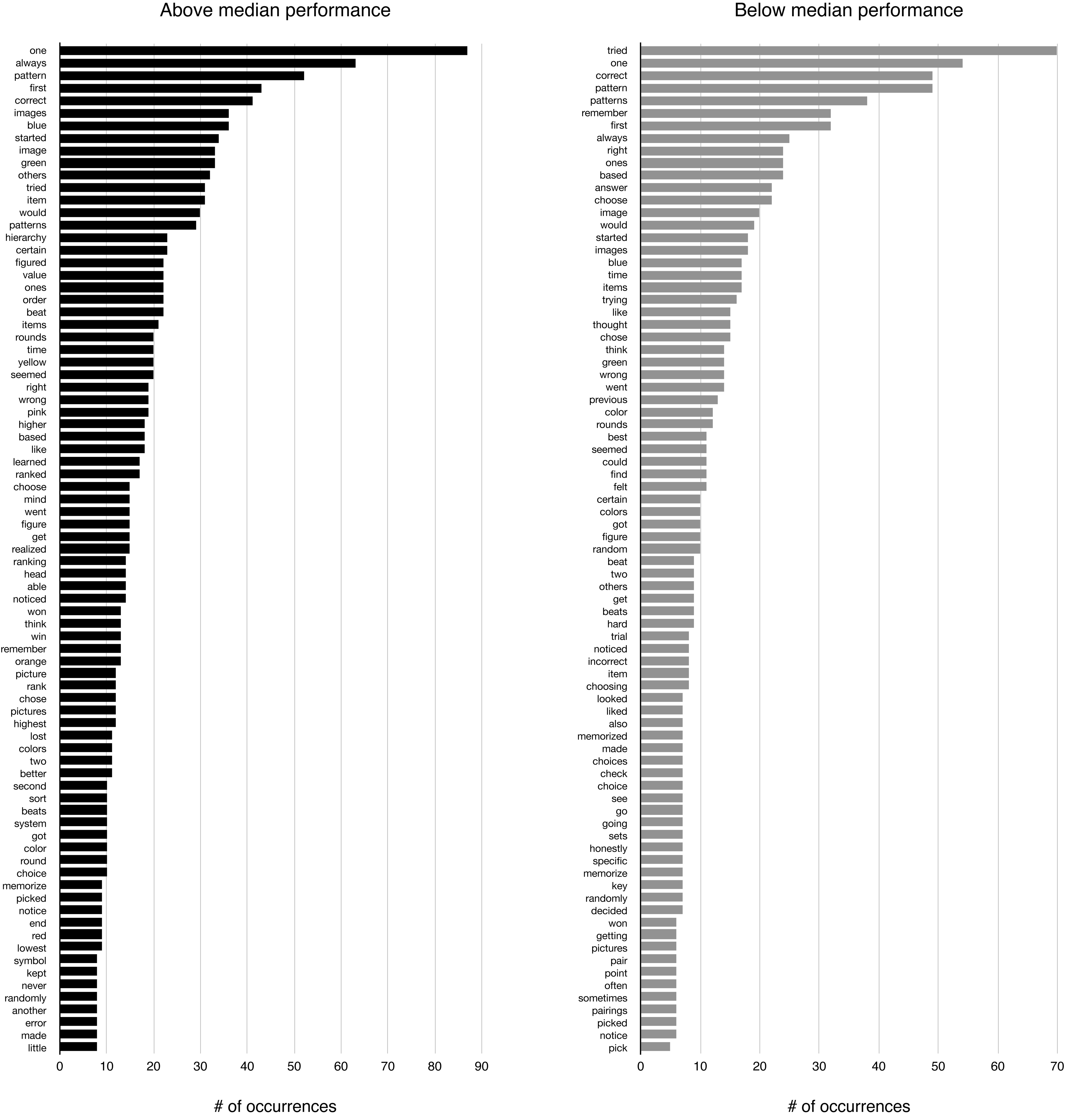

Supplement: S12 Fig — Word count summary (generated using wordcounter.ai) of typed responses to a question (“How did you decide which item to choose?”) in debriefing questionnaire in the human behavioral study of the delay TI task. (TIF) [file pcbi.1011954.s012.tif]

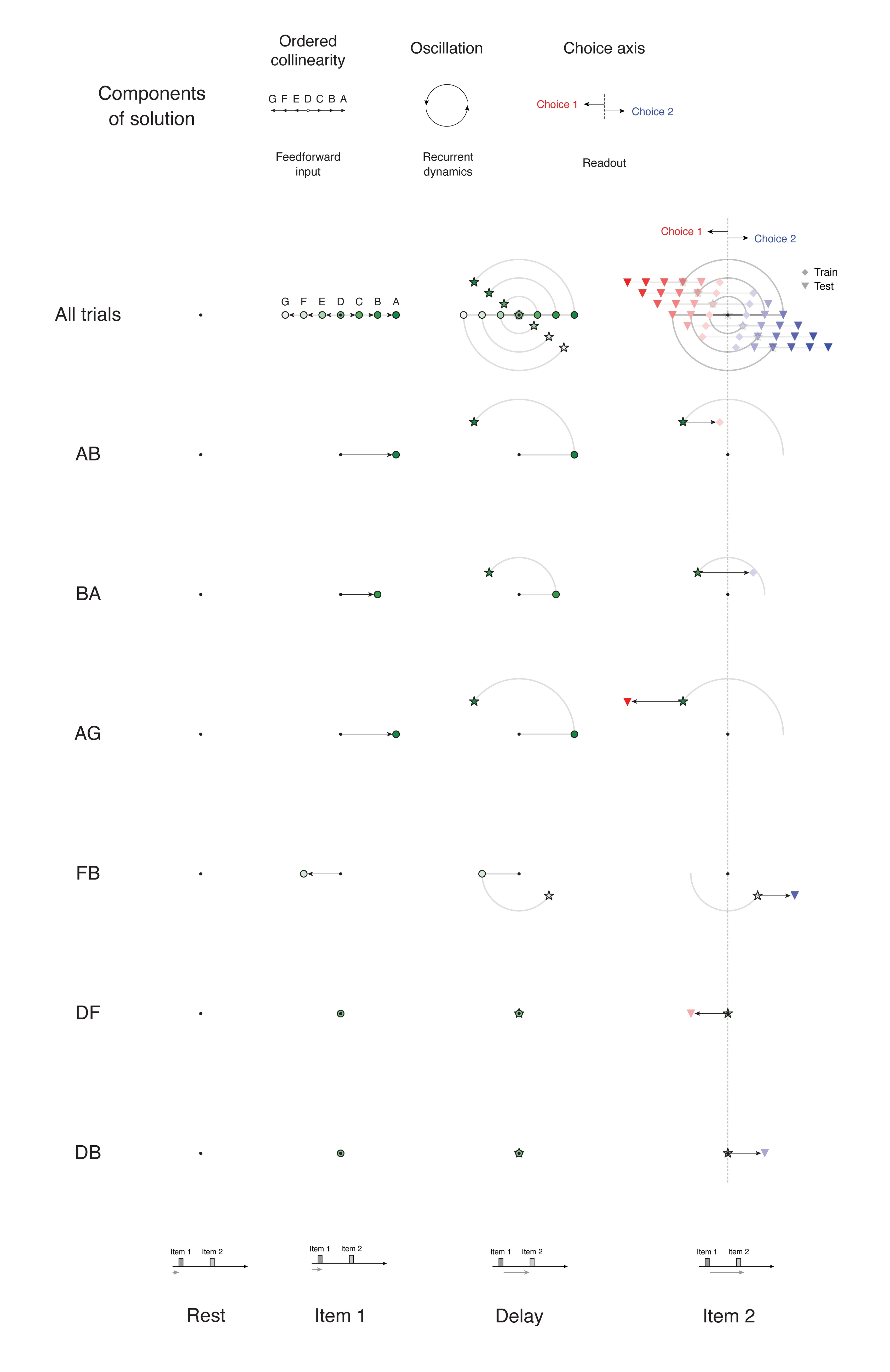

Supplement: S1 Appendix — Diagrams presenting the solution in greater detail (compare to Fig 5). Top, diagram of each of the population-level components comprising the solution (top: the specific form of the component; bottom: the network implementation). Bottom, activity trajectories across trial periods (columns; diagram of each period at bottom) and across different trial types (rows; top row: all trials; single trial types in rows below). Trajectories were generated by simulating a 2D linear dynamical system defined by an oscillation of frequency ∼0.5 cycles / delay, with initial condition at the origin and input vectors encoding task items (A, B, C, etc.) in ordered collinear arrangement in state space. Trial-based input (item 1—delay—item 2, see S1A Fig) was applied to the system. (TIF) [file pcbi.1011954.s013.tif]
