# Supplementary material for: Emergent neural dynamics and geometry for generalization in a transitive inference task
Source: PLoS Comput Biol. 2024 Apr 25;20(4):e1011954. doi: 10.1371/journal.pcbi.1011954 (PMC11125559; doi:10.1371/journal.pcbi.1011954)
Supplement: S1 Table — Each entry corresponds to a particular input format (basic, extended, and variable delay) and RNN variant (learned connectivity: f-RNN, r-RNN, ff-RNN; constraint regime: higher to lower, see Table 1). (PDF) [file pcbi.1011954.s014.pdf]

| | Basic delay | | | Extended delay | | | Variable delay | | |
|---|---|---|---|---|---|---|---|---|---|
| | f-RNN | r-RNN | ff-RNN | f-RNN | r-RNN | ff-RNN | f-RNN | r-RNN | ff-RNN |
| Highest | 200 | 200 | 81 | 200 | 200 | 0 | 196 | 92 | 0 |
| High | 200 | 181 | 40 | 192 | 200 | 0 | 190 | 60 | 0 |
| Intermediate | 198 | 165 | 43 | 191 | 190 | 171 | 170 | 16 | 0 |
| Low | 192 | 110 | 35 | 187 | 116 | 100 | 149 | 7 | 5 |
| Lowest | 162 | 65 | 34 | 148 | 52 | 31 | 89 | 2 | 5 |
