# Supplementary material for: Emergent neural dynamics and geometry for generalization in a transitive inference task
Source: PLoS Comput Biol. 2024 Apr 25;20(4):e1011954. doi: 10.1371/journal.pcbi.1011954 (PMC11125559; doi:10.1371/journal.pcbi.1011954)
Supplement: S2 Table — Examples of typed responses to a question (“How did you decide which item to choose?”) in a debriefing questionnaire given to subjects after completing all trials in the delay TI task. Example responses are sorted by performance on test trial types averaged over the last three blocks of trials. See S12 Fig for word count summary. Subjects performing at high levels tended to use words indicating understanding of the transitive relationship (e.g. “hierarchy”, “order”, “higher”, “ranked”). Lower-performing subjects appeared to use these words less often, rather using phrases like “random”, “tried to remember”, or referring to other strategies or a lack thereof (e.g. “didn’t have a specific strategy”, “the more attractive image”, or strategies based on a single item, “choose one and if it was right I would keep choosing the same one”). Words denoting a comparative relationship between items were commonly used (e.g. “beat”, “win”, “lost”). A number of subjects at various performance levels mentioned that some items were always correct. (PDF) [file pcbi.1011954.s015.pdf]

Test accuracy %	Reported choice strategy
100	I learned the pattern and ranking of images and tried to memorize it
100	I figured out that each picture has a rank After a few rounds I memorized which picture has which rank
100	I assigned a name to each design in my head then I guessed at random until the hierarchy became apparent to me
100	I learned which item won against the other There was also one item that was always right and one that was always wrong to choose
100	I started by randomly picking items Then I started to notice patterns such as the teal green symbol was always correct and the tiedye circle was always incorrect Over time I was able to build up the relational value between the different items so that I could choose with intention rather than guessing
100	I ranked the items by their strength based on the matchups they won
100	At first I guessed until I learned a pattern Once I learned which pictures were winners over others it became easy
100	At first I figured out the best and worst ones After that I think I just deduced the correct order of value through trial and error
100	I slowly compiled a ranked list in my head gave them all short names and sort of made a little game out of it Like playing War with a deck of cards
100	I gradually learned which colorsitems never won and those which seemed to always win Then I started testing the frequent winners against the others until I ironed in a hierarchy of quality between each item That way I knew the which one was the best 2nd best 3rd and so on
100	At first it was obviously guessing but they didnt change value between rounds so I eventually learned the order The images were ranked and clicked on the higher rank one always ended up being correct
100	I assigned each image a color and tried to have a hierarchy of what beat what For example the orange tiger eye always seemed to win and the pink one always seemed to lose Between the blues they seemed to be the same except against each other and I just remembered which one beat which Sometimes it wouldnt work but it might have been user error pressing wrong key From there it was just trying to remember what beat what consistently
100	By the end I found there was a hierarchal structure for the symbols Once I memorized the hierarchy I picked whichever one was higher
100	I learned over time which image beat which other image in each pair and then mentally aligned them in a hierarchy Over the course of a few attempts I learned which image was at the top of the stack and which was at the bottom and sorted them in order of value Orange blue with purple yellow splash green blue with yellow pink orange blotch for example The higher positions on the list beat all the images below it
99	I started to notice that certain patterns were always correct against other patterns Such as one pattern was always incorrect and one was always correct
99	After a few rounds it became obvious that the different images had different values almost like cards in a deck of playing cards Once I was aware of which images had a higher value relative to the other images I always chose the image with the highest value and as long as I didnt flub the key press it always worked
99	I initially was guessing based on what I liked Then I started to notice the pattern one always winning one always losing and some depends on which it was paired with
99	First I believed there to be a pattern in first and second choice Then I found that certain images would beat others and believed some were weak to others and stronger than other ones Finally I realized they were ordered in a hierarchy and discovered the order from strongest to weakest shortly after

98	I wasnt sure for the first couple rounds then I figured out there was a hierarchy of the symbols
98	Whether or not I thought I was going to get a green checkmark or a red x
97	i noticed a pattern ofter the first 3 rounds
97	The first few rounds I didnt think I was going to be able to figure any kind of pattern out Over time it seemed that I started mentally attributing a value to each design Some were higher than others A certain blue image seemed to be a higher value than all the rest and a certain green seemed to be the lowest By the end of the trials this seemed to work for me
97	I ranked them mentally
97	By trial and error you eventually learn the ranking of the items and which one beats which It became apparent what the hierarchy was over time
97	At first I picked the ones I liked better I then realized there was a sort of rank between the images and I tried to remember the rank when I picked
97	I tried to pay attention to each pair and identify which choice was the correct one based on the feedback I received
96	Trial by elimination at the start until I learned the values and then just followed what I knew worked
96	It was random at first until I started noticing a pattern then I narrowed it down little by little until I mostly had the order
96	I tried to find patterns in the pairings One initial relationship I was able to figure out and remember throughout all the trials the green vs yellow Then eventually I realized that no matter what one of the colors always lost would always be the wrong choice and another would always win be the right choice no matter who they are paired with I will call them the ABSOLUTES Then I realized there was another that would mostly lose and one other that mostly win that is this remained true as long as they werent paired with an Absolute From there it got easier I did struggle with the pinkcolored patterns because I couldnt keep track of their relationships with each other as there were three of them
94	Which one had the most interesting pattern Then after a while I kind of memorized the hierarchy they have
94	randomly at first then i started to learn the relationship between the pictures Barring some mistypes I decided based on what I understood of the relationship between pictures
93	I suppose I eventually learned the hierarchy of items although it took me longer than it probably should have
93	At first it was hard Finally I picked up on the pattern In fact I could probably successfully put them in order while theyre still on my mind
92	I tried to pick up on the pattern It seemed like certain squares went with other squares and certain combos were correct when others were incorrect I tried to memorize the combos
92	I just paid attention to the patterns of wins and losses and tried to remember what beats what I realized there was one that never lost and one that either always lost or lost to most options and kept track of what lost and won when in between
92	I tried to create a hierarchy in my mind
91	I built up a mental model Some symbols were never chosen while others were always chosen For the others you had to learn the relative value of each symbol
91	I started off just randomly choosing an item But afterwards I noticed a pattern and started to try and memorize which item would be correct based on which other item it was up against

91	I managed to figure out which ones were better than others in terms of which ones were correct more often I eventually figured out one was always correct and one was always wrong
91	I found a pattern and went with it
90	I memorized the colors that would give me the correct answer and just trial and error
90	At first I made by choices based off what I liked I then started to choose based on what got the green check mark
90	It seemed like certain patterns were repeating so I choose the opposite option the next time if I failed the pattern initially or kept my answer if I choose the right answer initially I tried to remember as many as I could Also there were two options that had definitive answers regardless of the counter option
89	They seemed to have a ranking where 1 is correct over the other and I just tried to remember from the last time that they flashed on the screen which picture was the higher ranking of the two I kept getting it backwards on a couple of the pictures though
89	I tried to remember which one won the pair last time it was shown
88	At first I had literally no clue what was going on but I was getting a few correct After round one I put my brain on auto pilot and let my subconscious process what was happening I didnt think I just observed the pictures and let my brain figure it out It wasnt till round 4 or 5 when my conscious brain realized what I think was going on Its truly amazing what our subconscious brain figures out before we do What I think was going on with the images was if Im correct that they were numbered or ranked So I started asking myself Which is greater At first the plus sign confused me but I realized it was to be ignored and I just used it as a focal point for the image flashes If I was given those images and asked to stack them in order of importance I bet I could do it with some measure of accuracy Probably not perfect but still For example there was this monochromatic blue image with grid lines I think that one was the highest rank and there was a brown one that looked like animal print I think that one was lowest
88	They were repeated so many times throughout the rounds that eventually it became natural I memorized the ones that I thought were nice looking and went from there
84	At first I picked which ones were the most appealing to me As the task moved on I noticed a pattern between which ones were correct and which ones were not so I tried my best to remember which ones were correct over specific patterns
82	I went with my best judgement on which I thought would be correct and tried to take a mental note of which side certain colors seemed to be correct on
82	I chose which image looked better than the other There was a pattern 2 pairs of images always went with one another So I tried to decide which image was correct out the pair
81	At first I just chose which item I liked better then I started trying to notice patterns of items that always beat other items
80	At first it seemed like I was suppose to be choosing at random As I kept going I began to realize the pattern of what to click and what not to click as well
79	I gave each item a nickname and tried to rank them in my head so as to remember which items beat which
78	Honestly I chose randomly on some trials but Ive noticed that there were a couple of images that were always correct the golden 8 in the purple image and I think one of the blue images It was hard to keep track of everything though but I tried my best to do so
77	Some patterns were always win and some were always lose Some were in between where it was more of a toss up

71	I tried to remember patterns and which image led to the correct choice previously
68	It was sheer guesswork at first then I memorized SOME of the ones that seemed to work over others However it was still at least 67 a complete shot in the dark
68	I eventually began recalling the pairings from previous questions of the two same patterns I started to memorize which one was the correct one of the two designs with more frequent practice
66	Some of the graphics seemed to be correct more often than othersso I tried to choose those ones most of the time
66	Based on whether or not my previous choices with a certain item was incorrect
60	I would choose one and if it was right I would keep choosing the same one If it was wrong I would switch to the other one and keep choosing that one until it was wrong
57	I started with choosing randomly and tried to figure out patterns from there I was not always able to remember the specific correct choice for each pair but I figured out that there was one and I did my best to choose that one each time
56	I didnt think to much about the choices at first but then I tried to choose based on the previous round to see if there was a pattern to go by
54	I tried to find patterns or relations between the images
50	Just went on with flow and the more attractive image
50	I tried to remember which answer was correct when a repeating set of pictures was used I basically just tried to figure out remember what the correct answer was for each set
50	Some rounds I tried to notice patterns but most of the times it was completely random
49	Honestly it felt random At first I thought it was based on the patterns but it felt really random
42	I was trying to figure out which one was the correct one when shown with a different picture Its a lot harder than I thought and I think I did pretty terrible I tried but still could not figure this out
42	The item was most familiar but it felt so random
41	I went for the color I either liked most or made me the least uncomfortable
39	i didnt have a specific strategy to choose
38	Just tried to remember the combinations as I go
